# Novel influenza A viruses in pigs with zoonotic potential, Chile

**DOI:** 10.1128/spectrum.02181-23

**Published:** 2024-03-07

**Authors:** Rodrigo Tapia, Bárbara Brito, Marco Saavedra, Juan Mena, Tamara García-Salum, Raveen Rathnasinghe, Gonzalo Barriga, Karla Tapia, Victoria García, Sergio Bucarey, Yunho Jang, David Wentworth, Montserrat Torremorell, Víctor Neira, Rafael A. Medina

**Affiliations:** 1 Universidad de Chile, Santiago, Chile; 2 Department of Pediatric Infectious Diseases and Immunology, School of Medicine, Pontificia Universidad Católica de Chile, Santiago, Chile; 3 University of Technology Sydney, Sydney, New South Wales, Australia; 4 Centers for Disease Control and Prevention (CDC), Atlanta, Georgia, USA; 5 University of Minnesota, St. Paul, Minnesota, USA; 6 Department of Pathology and Experimental Medicine, School of Medicine, Emory University, Atlanta, Georgia, USA; 7 Department of Microbiology, Icahn School of Medicine at Mount Sinai, New York, New York, USA; Xinxiang Medical University, Xinxiang, China

**Keywords:** influenza A virus, pigs, zoonosis

## Abstract

Novel H1N2 and H3N2 swine influenza A viruses (IAVs) have recently been identified in Chile. The objective of this study was to evaluate their zoonotic potential. We perform phylogenetic analyses to determine the genetic origin and evolution of these viruses, and a serological analysis to determine the level of cross-protective antibodies in the human population. Eight genotypes were identified, all with pandemic H1N1 2009-like internal genes. H1N1 and H1N2 were the subtypes more commonly detected. Swine H1N2 and H3N2 IAVs had hemagglutinin and neuraminidase lineages genetically divergent from IAVs reported worldwide, including human vaccine strains. These genes originated from human seasonal viruses were introduced into the swine population since the mid-1980s. Serological data indicate that the general population is susceptible to the H3N2 virus and that elderly and young children also lack protective antibodies against the H1N2 strains, suggesting that these viruses could be potential zoonotic threats. Continuous IAV surveillance and monitoring of the swine and human populations is strongly recommended.

**IMPORTANCE:**

In the global context, where swine serve as crucial intermediate hosts for influenza A viruses (IAVs), this study addresses the pressing concern of the zoonotic potential of novel reassortant strains. Conducted on a large scale in Chile, it presents a comprehensive account of swine influenza A virus diversity, covering 93.8% of the country’s industrialized swine farms. The findings reveal eight distinct swine IAV genotypes, all carrying a complete internal gene cassette of pandemic H1N1 2009 origin, emphasizing potential increased replication and transmission fitness. Genetic divergence of H1N2 and H3N2 IAVs from globally reported strains raises alarms, with evidence suggesting introductions from human seasonal viruses since the mid-1980s. A detailed serological analysis underscores the zoonotic threat, indicating susceptibility in the general population to swine H3N2 and a lack of protective antibodies in vulnerable demographics. These data highlight the importance of continuous surveillance, providing crucial insights for global health organizations.

## INTRODUCTION

Influenza A virus (IAV) circulates endemically in nature in hosts, such as birds, dogs, horses, swine, and humans, representing a constant concern to both animal and public health worldwide (1). Swine have an important role in the ecology and evolution of IAV since they are susceptible to both avian and human strains. They have been proposed as an intermediate host for interspecies transmission and a “mixing vessel” for the generation of novel reassortant strains, combining virus genes adapted to different host species. These reassortant strains could have zoonotic and pandemic potential, representing a public health concern (2). A clear example occurred during the 2009 pandemic, when a novel reassortant H1N1 IAV emerged, designated as A(H1N1)pdm09, which shared gene segments from swine, avian, and human IAV genes (3, 4).

IAVs in swine are genetically and antigenically diverse, and several lineages have been reported (5, 6). Antigenically novel IAVs can be generated when the segments encoding the surface glycoproteins hemagglutinin (HA) and neuraminidase (NA) are reassorted, a process known as “antigenic shift.” IAV genetic and antigenic diversity also arises through the high mutation rate inherent to the replication of this virus. The accumulation of non-synonymous mutations in the glycoproteins because of immune selection pressure is known as “antigenic drift.” Mutations in the HA can cause major changes in the antigenicity of the virus, which may reduce the effectiveness of vaccines administered to pigs or humans, and might potentially result in increased zoonotic risk. Therefore, it is essential to carry out swine IAV surveillance worldwide and to evaluate the zoonotic potential of the novel strains.

Novel H1N2 and H3N2 swine IAVs have recently been identified in Chile. The HA genes of these viruses were likely introduced from humans in the past and now are genetically distant from other IAVs identified in swine and humans globally, including commercial vaccine strains used in pigs. These viruses replicated and were shed, without prior adaptation, in the upper respiratory tract of guinea pigs, which were used to evaluate their infection dynamics (7–9). These findings suggest that these viruses could have public health importance, emphasizing the need to carry out further studies to evaluate the zoonotic potential of these viruses.

Here, we performed a systematic swine and human molecular epidemiological study, analyzing all gene segments to determine the genetic diversity, predominant lineages, and the origins and evolution of swine IAVs circulating in commercial farms in Chile. We also performed a serological analysis to evaluate the presence of cross-reactive antibodies against these swine viruses in the Chilean human population.

## MATERIALS AND METHODS

### Swine study design and sample collection

All animal procedures were approved by the Institutional Animal Care and Use Committees of both, Universidad de Chile (protocol numbers 20-2015 and 02-2016) and Pontificia Universidad Católica de Chile (PUC, protocol number 13-001).

Commercial swine farms were invited to participate in an active IAV surveillance program. Enrolled herds were representative of modern swine production systems and included breeding herds, nurseries, finishers, and wean-to-finish farms. From December 2013 to January 2015, samples were obtained during a total of 55 visits to 39 swine farms (24 companies) comprising 93.8% of the commercial farms in Chile. Farms were in the central regions of Chile (Metropolitana, Valparaíso, O’Higgins, Maule, Biobío, and Araucanía Regions), where ~99% of the intensive pig production is found (10). Due to confidentiality agreements, the geographical locations of the farms are not disclosed. During each visit, nasal swabs and oral fluids were collected from pigs 6–14 weeks of age, which is the age when there is an increased probability of influenza detection (11). Samples were obtained from pigs with influenza-like clinical signs, which included coughing, sneezing, fever, and/or lethargy. If no animals were found with influenza-like signs during the visit, samples were obtained randomly. For nasal swab collection, 30 animals were selected and manually restrained, and nylon-flocked swabs were inserted into both nostrils before being placed in tubes with 1 mL of universal viral transport media (12). The sample size per visit was estimated to detect at least one IAV-positive sample assuming ≥10% prevalence of IAV in the farm and considering 95% confidence of viral detection. Additionally, approximately six oral fluid samples were obtained from swine of the same age using 1 m of twisted cotton rope hanging for 20 min in each pen. Ropes were squeezed into a sterile plastic bag, and the oral fluids were deposited into a sample tube (9). Additional samples were also submitted for diagnostics (including lung tissue) when suspected cases of IAV were observed. Samples collected were refrigerated and immediately transported on ice to the laboratory for processing.

### Diagnostic tests

To detect antibodies to any influenza A subtype, 50-µL serum samples were tested using the Influenza A Ab Test Kit (IDDEX, Westbrook, Maine, USA). Oral fluids were centrifuged at 5,000 rpm for 20 min, and the supernatant was stored in aliquots at ­80°C until further processing. Both nasal swabs and oral fluids were tested for IAV using real-time RT-PCR (rtRT-PCR) and/or virus isolation. For rtRT-PCR, viral RNA was first extracted using the TRIzol LS Reagent (Invitrogen, Carlsbad, CA, USA) according to the manufacturer’s instructions and then followed by amplification of the matrix gene using a previously described protocol for IAV diagnosis (13). rtRT-PCR results with cycle threshold (Ct) <35 were considered positive.

Virus isolation was attempted in rtRT-PCR-positive samples. This test was carried out in Madin-Darby Canine Kidney (MDCK) cells using minimum essential medium (MEM) supplemented with 10% fetal calf serum (FCS) and 1% antibiotic-antimycotic solution. Briefly, the MDCK monolayers were washed twice to remove FCS using PBS containing 1 µg/mL of trypsin treated with N-tosyl-L-phenylalanyl chloromethyl ketone (TPCK) (Sigma-Aldrich, St. Louis, Mo, USA), inoculated with each sample, and incubated for virus absorption for 1 h at 37°C. Subsequently, cells were rinsed with PBS to eliminate unbound virus, and IAV growth medium (MEM supplemented with 1 µg/mL of TPCK trypsin, 0.3% bovine serum albumin, and 1% antibiotic-antimycotic solution) was added. The monolayers were incubated at 37°C. Each sample was observed for cytopathic effect (CPE) daily for up to 5 days. Plates without CPE were passaged again and observed for another 5 days. Samples with no CPE after the second passage were considered negative for IAV. CPE-positive samples were tested by hemagglutination assay using turkey erythrocytes (14) and rtRT-PCR to confirm the presence of IAV.

### Viral sequencing

Fifty-two IAV isolates were sequenced by whole-genome sequencing, and 18 IAV isolates were by Sanger (HA gene). For whole-genome sequencing, the samples were prepared using a multisegment RT-PCR (mRT-PCR), following the protocol described by Zhou et al. (15). Eluted RNA from each positive sample was used as template to synthesize cDNA using primer MBTuni-12 (5′-ACG CGT GAT CAG CRA AAG CAG G-3′) and Superscript III First Strand Synthesis SuperMix (Invitrogen, Carlsbad, CA, USA), following the manufacturer’s instructions. Then the cDNA was amplified by five cycles (94°C for 30 s, 45°C for 30 s, and 68°C for 3 min) and then 31 cycles (94°C for 30 s, 57°C for 30 s, and 68°C for 3 min). The total volume for the RT-PCR reaction was 50 µL using a High-Fidelity DNA Polymerase (Agilent, Santa Clara, USA), with MBtuni-12 and MBtuni-13 (5′-ACG CGT GAT CAG TAG AAA CAA GG-3′) primers (buffer: 5 µL, 10 mM dNTPs: 1 µL, 1.5 µL of each primer, Picomax Enzyme: 1 µL, water: 35 µL, and 5 µL of cDNA). PCR products were run in a 1.5% agarose gel by electrophoresis and purified using QIAquick Spin Kit (QIAGEN, Valencia, CA, USA). Purified PCR products with ≥25 ng/µL of DNA concentration were submitted to the Center for Research on Influenza Pathogenesis Sequencing Core at the Icahn School of Medicine at Mount Sinai, for library preparation and sequencing using next-generation sequencing (NGS) technologies (Illumina HiSeq2000). Illumina reads were mapped to an IAV reference genome using Bowtie2, and consensus sequences were extracted using SAMtool. The HA Sanger sequences were obtained from IAV isolates submitted for diagnosis to the Veterinary Diagnostic Laboratory at the University of Minnesota (VDL-UMN) in 2012.

### Human sample collection and A(H1N1)pdm09 virus sequencing

All the patient and volunteer samples used in this study were collected after written informed consent was obtained under protocols 11-116 and 09-203, which were reviewed and approved by the Scientific Ethics Committee of the School of Medicine at PUC before the start of sample collection. Viral isolates prior to 2009–2011 were obtained anonymously through the Clinical Diagnostic Laboratory of the UC-Christus Health Network as part of their seasonal epidemiological surveillance.

To elucidate the diversity and time of introduction of the A(H1N1)pdm09-like viruses, we sequenced the full genome of 114 A(H1N1)pdm09 viruses obtained from infected individuals during 2009–2013 in Chile. Viral RNA was purified using a ZR 96 Viral RNA Kit (Zymo Research). The IAV genomic RNA segments were simultaneously amplified from 3 µL of purified RNA using the mRT-PCR described above (15, 16). The influenza genome amplicons were barcoded and amplified using an optimized sequence-independent single primer amplification (SISPA) protocol (17, 18). Subsequently, the SISPA amplicons were purified, pooled, and size selected (∼800 or ∼200 bp), and the pools were used for library construction, and the complete coding genomes of the IAVs were then sequenced using a high-throughput NGS pipeline at the J. Craig Venter Institute using the 454/Roche GS-FLX platform and Titanium chemistry or the Illumina HiSeq 2000 platform. The sequence reads were sorted by barcode, trimmed, and searched by TBLASTX against custom nucleotide databases of full-length IAV segments downloaded from GenBank to filter out both chimeric influenza sequences and non-influenza sequences amplified during the random hexamer-primed amplification. The reads were binned by segment, and the 454/Roche GS-FLX reads were *de novo* assembled using the clc_novo_assemble program (CLC Bio). The resulting contigs were searched against the corresponding custom full-length influenza segment nucleotide database to find the closest reference sequence for each segment. Both, the 454/Roche GS-FLX and Illumina HiSeq 2000 reads were then mapped to the selected reference IAV segments using the clc_ref_assemble_long program (CLC Bio). At loci where both 454/Roche GS-FLX and Illumina HiSeq 2000 sequence data agreed on a variation (as compared with the reference sequence), the reference sequence was updated to reflect the difference. A final mapping of all next-generation sequences to the updated reference sequences was then performed. Any regions of the viral genomes that were poorly covered or ambiguous after next-generation sequencing were amplified and sequenced using the standard Sanger sequencing approach.

All gene sequences obtained from this study were submitted to GenBank under accession numbers (Table S1) and were considered for the phylogenetic analyses.

### Phylogenetic analysis

Reference human and swine sequences from different known influenza lineages, and the most similar sequences available in GenBank obtained using the BLAST tool from NCBI (http://blast.ncbi.nlm.nih.gov/Blast.cgi), were used to reconstruct the genetic phylogeny of the Chilean swine viruses. Sequences of each of the segments were aligned using MUSCLE, and then a Bayesian evolutionary analysis was performed by sampling three methods using the BEAST 1.8.2 software (19). We used the Partition finder software to select the substitution model (20) and codon partition with the lowest Bayesian information criteria and used the uncorrelated exponential clock model and a coalescent Bayesian skyline tree, prior to running the analyses. Each segment phylogeny was run for 500 million generations, sampling every 50,000 trees. Convergence and mixing of the simulations were assessed using Tracer, and we then used Figtree v1.4.2 (21) to visualize the maximum clade credibility tree (MCC). The time to the most recent common ancestor (TMRCA) and substitution rates for specific branches were obtained from the MCC. Trees were illustrated highlighting Chilean swine, Chilean human, South American swine, and other reference sequences from human and swine as shown. For the phylogenetic analyses, we used an alignment of 296 sequences of the H1pdm09 viruses, 156 H1 sequences with viruses from the delta swine cluster and human seasonal strains, and 261 H3 sequences, including the viruses sequenced in this study. Alignments of sequences obtained in this study and reference sequences used to reconstruct the phylogeny of internal genes comprised: 189 sequences for PB2, 188 sequences for PB1, 198 sequences for PA, 163 sequences for NS, 204 sequences for NP, 210 sequences for NA (N2), 259 sequences for NA (N1pdm09), and 205 sequences for M.

To determine the phylogenetic relationship of the novel Chilean swine viruses, seasonal human viruses and human vaccine stains were used with a total of 185 H1 and 173 H3 HA sequences from GISAID/GenBank databases to make backbone trees for each H1 and H3 swine lineages. In addition to the backbone trees, 41 H1 and 2 H3 swine Chilean sequences were added to the tree for phylogenetic analysis. Data were aligned via MUSCLE (22), and sequences were trimmed to the beginning of mature H1 HA protein gene sequence using BioEdit v7.0. Approximate maximum likelihood trees (Juke-Cantor model) were constructed using the Mega 7.0 software package (23).

### Analysis of human antibody cross-protection against swine IAVs

Hemagglutinin inhibition (HI) assay was performed to determine the cross-reactive antibody titers in the general population against the ChH1N2, H1N1, and H3N2 swine IAVs. Between July 2009 and December 2015, a total of 237 blood samples were collected from individuals diagnosed with IAV in Santiago, Chile. Serum samples were obtained by centrifugation at 3,000 rpm for 5 min and were then inactivated by trypsin-heat-periodate treatment. Antibody titers against the Chilean swine viral strains were determined by the HI assay as previously described (24).

## RESULTS

### Swine IAVs circulate endemically in commercial swine farms in Chile

To obtain representative samples of the swine IAV circulating in commercial swine farms in Chile, we sampled 39 farms representing 93.8% of the industrialized farms in the country. We collected a total of 1,237 samples, including 1,046 (84.6%) nasal swabs, 188 (15.2%) oral fluids, and 3 lungs (0.2%). Of these, 235 (22.5%) nasal swabs, 68 (36.2%) oral fluids, and 3 lungs (100%) were tested positive for IAV by real-time-RT-PCR, which resulted in a total of 306 (24.7%) positive samples. Twenty eight out of 39 (72%) farms tested positive for IAV at least once during the study. Positive IAV farms were found in all geographical regions sampled.

### Novel swine IAVs of human origin circulating in Chile

**Fig 1:**
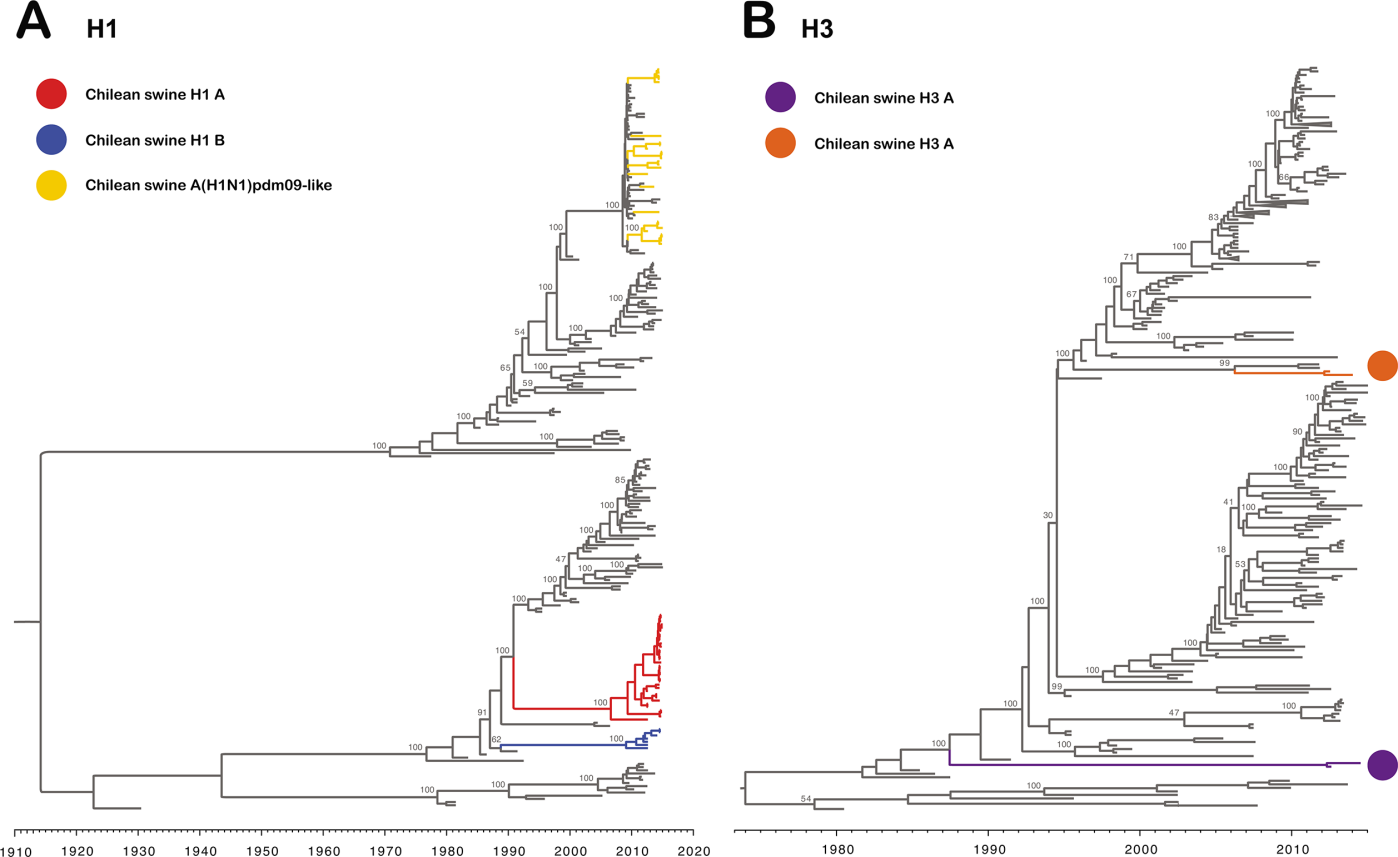
Novel H1 and H3 swine influenza viruses identified in Chile. Maximum clade credibility trees depicting TMRCA estimates, reconstructed using HA gene sequences of human and swine IAVs reported globally. (**A**) Tree reconstructed using H1 subtype sequences. Branches of the tree containing the sequences from novel Chilean H1 swine IAVs are highlighted in red, whereas branches with sequences from the Chilean swine A(H1N1)pdm09-like IAVs are colored in brown. (**B**) Tree reconstructed using H3 subtype sequences. Branches with novel Chilean H3 swine IAVs are highlighted in yellow.

**Fig 2:**
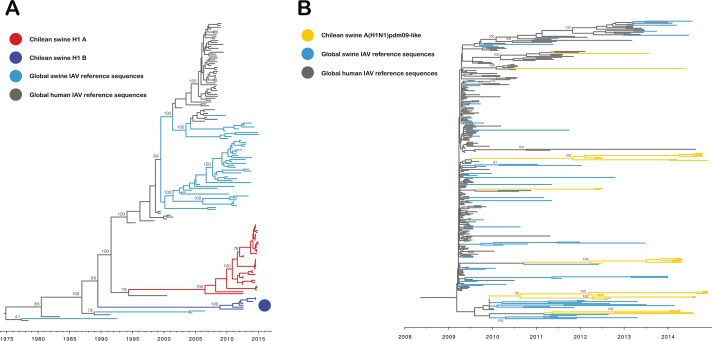
Two divergent H1N2 swine IAVs derived from old human seasonal viruses co-circulate with A(H1N1)pdm09-like strains. Maximum clade credibility tree constructed depicting TMRCA estimates of HA gene sequences of the H1 subtype. (**A**) Novel Chilean H1 swine IAVs are grouped into two phylogenetic clusters (ChH1A and ChH1B), highlighted in red. The closest reference sequences, from the human seasonal and swine delta clusters, were used. Chilean human IAV sequences are highlighted in blue. (**B**) H1 maximum clade credibility tree constructed using only sequences belonging to the A(H1N1)pdm09 IAV lineage. Chilean swine sequences are highlighted in brown and are interleaved with human reference sequences, including Chilean human sequences, suggesting at least seven different introductions from human to swine (●).

To gain a comprehensive understanding of the IAV diversity in the country, we sequenced a total of 70 IAV isolates (52 whole-genome sequences and 18 HA sequences) from 26 farms. Of the 70 HA sequences analyzed, 68 belonged to the H1 subtype and 2 to the H3 subtype. Forty-one H1 sequences were grouped in two major monophyletic clusters, which are genetically distant from other IAVs described worldwide (Fig. 1A and 2A). These clusters were highly divergent between them, with up to 14% nucleotide differences, and were named Chilean H1A (ChH1A; 34/41) and Chilean H1B (ChH1B; 7/41), as previously proposed by Tapia et al. (8). These clusters were classified as “Other-Human-1B.2,” according to the new global clade designation (6). The ChH1A lineage appears to have spread more efficiently in pigs since it was found in five farms that were epidemiologically related, while ChH1B was only found in two farms. The most closely related virus to the cluster ChH1A was a human IAV from 2000 [A/Chile/4795/2000(H1N1)]. The time to the most recent common ancestor (TMRCA) to the cluster ChH1A was estimated in August 2006 (95% highest posterior density (HPD) November 2000–September 2009). The TMRCA for cluster ChH1B was estimated in February 2009 (95% HPD March 2004–November 2010) (Fig. 2A). Overall, the phylogeny suggests that both clusters are of human origin, which have been derived from viruses that were introduced into pigs previous the A(H1N1)pdm09 pandemic and that became endemic within the Chilean swine population during those years.

All other H1 sequences (27/68) were classified into an A(H1N1)pdm09-like cluster (Fig. 1A and 2B). To elucidate the diversity and time of introduction of the swine A(H1N1)pdm09-like viruses, we isolated and obtained the full-genome sequences of 114 A(H1N1)pdm09 viruses from humans infected between 2009 and 2013 in Chile, which were used as reference sequences in the phylogeny. The phylogeny of the HA gene showed an interleaved pattern of A(H1N1)pdm09-like viruses collected from both species, suggesting at least seven independent introductions of these viruses into swine populations after the 2009 H1N1 human pandemic (Fig. 2B). In fact, two swine A(H1N1)pdm09-like viruses were closely clustered with human A(H1N1)pdm09 viruses from Chile, which were obtained a year earlier. This reveals a dynamic human-swine transmission of A(H1N1)pdm09-like IAVs, which are highly disseminated in the swine population, as they were found in 12 different farms that were not geographically related.

The H3 sequences were related to two human-like H3 IAV singletons reported recently. These Chilean H3 sequences are not related to other swine H3 sequences reported globally, including the North American swine clusters (I-IV). The TMRCA of one H3 sequence was estimated in April 2006 (95% HPD September 1998–January 2009). The other H3 sequence was highly divergent from any sequence published elsewhere and had an estimated TMRCA in June 1987 (95% HPD June 1984–May 1990) (Fig. 1B). Each H3 virus was found in a single farm, which suggests that they were introduced and maintained in these farms during this time.

**Fig 3:**
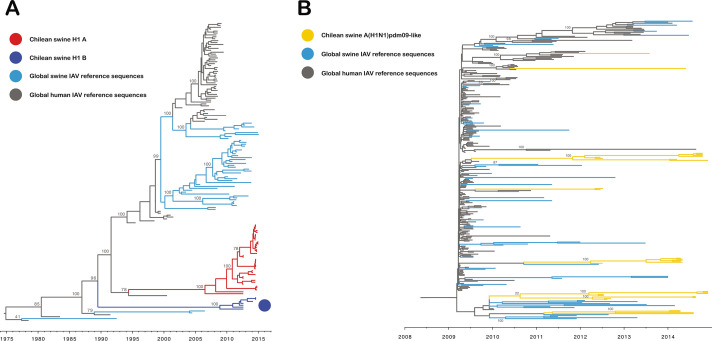
All N1 segments belong to the A(H1N1)pdm09-like lineage, and most of the N2 genes belong to a novel N2 cluster named Chilean N2. Maximum clade credibility trees depicting TMRCA estimates of NA gene segment of human and swine IAVs. (**A**) Four N1 lineages are shown in the tree: human seasonal, North American classic swine, Eurasian avian like, and N1 A(H1N1)pdm09 like. All Chilean swine N1 sequences belong to the A(H1N1)pdm09-like lineage and are colored according to the original HA clustering (Fig. 1 and 2). (**B**) N2 sequences are colored according to the original HA clustering (Fig. 1 and 2). Most of the N2 sequences are grouped in a major monophyletic cluster, named Chilean, and are associated to H1 genes from the Chilean H1 clusters (ChH1A and ChH1B).

In the case of the NA genes, they belonged to either the N1 (24/52) or N2 (28/52) subtypes. All N1 segments were derived from the human A(H1N1)pdm09-like N1 cluster (Fig. 3A). The phylogeny shows that N1 sequences are grouped with other human and swine-origin A(H1N1)pdm09-like sequences, also suggesting at least seven independent introductions into the swine population, like the phylogeny based on the HA gene (Fig. 3A). Most of the N2 sequences (89%, 25 out of 28) were grouped into a monophyletic cluster, suggesting a single introduction of this segment that became widespread via reassortment and evolved within the swine population (Fig. 3B). These N2 sequences were clustered separately and are divergent from other N2 sequences reported in swine elsewhere; hence, we propose to name this cluster Chilean N2 (ChN2). The TMRCA for this main cluster of N2 was September 2007 (95% HPD January 2002–November 2010). These ChN2 sequences are associated to H1 sequences from the Chilean H1 clusters (ChH1A and ChH1B), but they were related to N2 segments present in human H3N2 seasonal viruses from the 1990s. There were three additional N2 sequences, which were not part of the major cluster and were not related among them. Two of these N2 sequences corresponded to two singletons that are associated with H3 sequences (Fig. 3B). One of these N2 sequences shared an early common ancestor with the ChN2 sequences. The TMRCA of this sequence was February 1989 (95% HPD July 1983–September 1992). The TMRCA of the other N2 singleton was February 2001 (95% HPD March 1997–December 2005).

### Complete human-derived A(H1N1)pdm09 internal gene cassette is present in all Chilean swine IAVs

**Fig 4:**
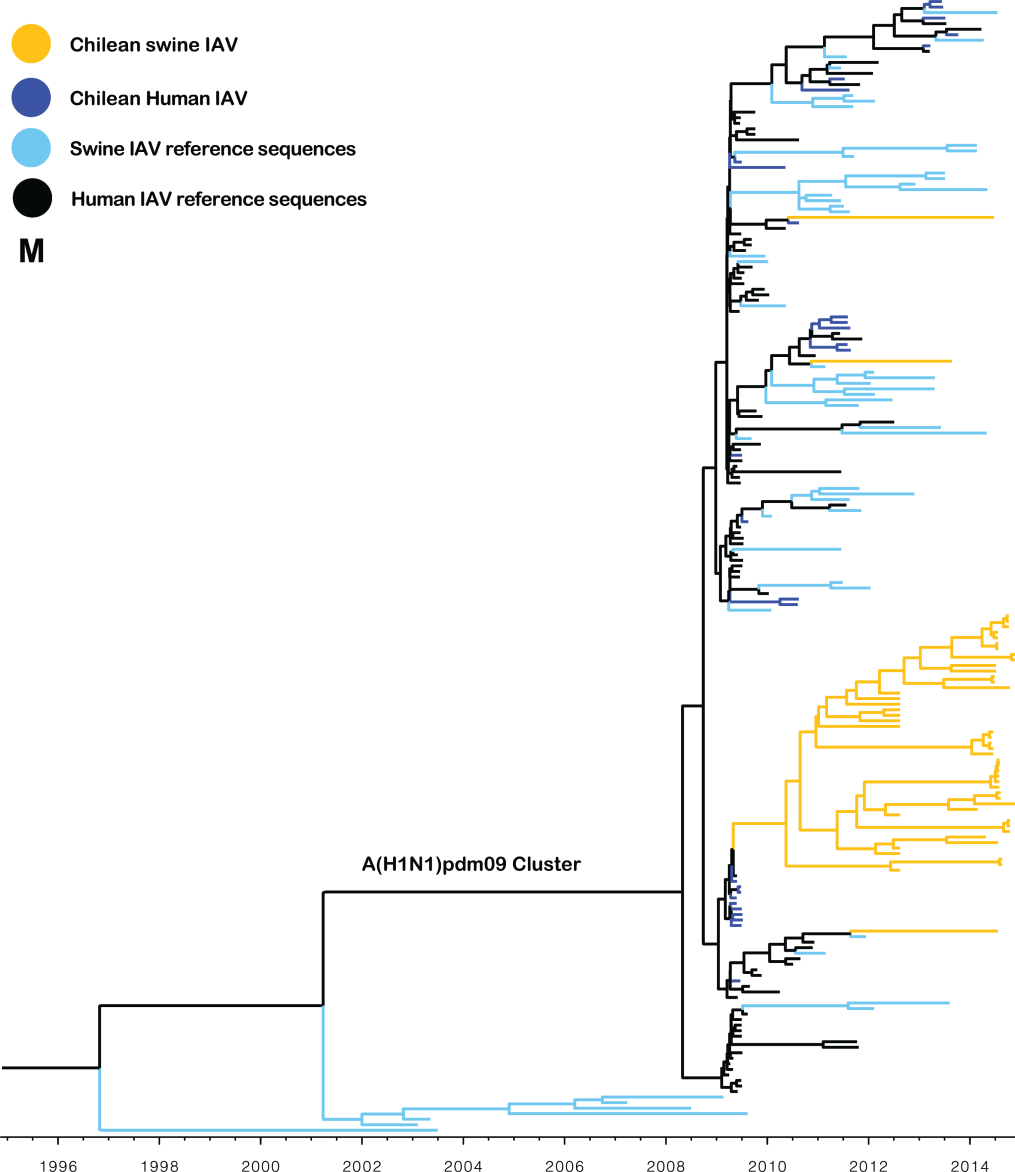
The M gene segment of Chilean swine IAVs is derived from early A(H1N1)pdm09 human strains. Maximum clade credibility tree depicting TMRCA estimates of M gene segment of human and swine IAVs. All Chilean swine M sequences belong to the A(H1N1)pdm09-like lineage. Most Chilean swine viruses were grouped within the same cluster and had a most recent common ancestor with A(H1N1)pdm09 human strains from 2009. The M gene segment from three swine A(H1N1)pdm09-like strains and from swine H3 strains was grouped with other A(H1N1)pdm09-like viruses.

**Fig 5:**
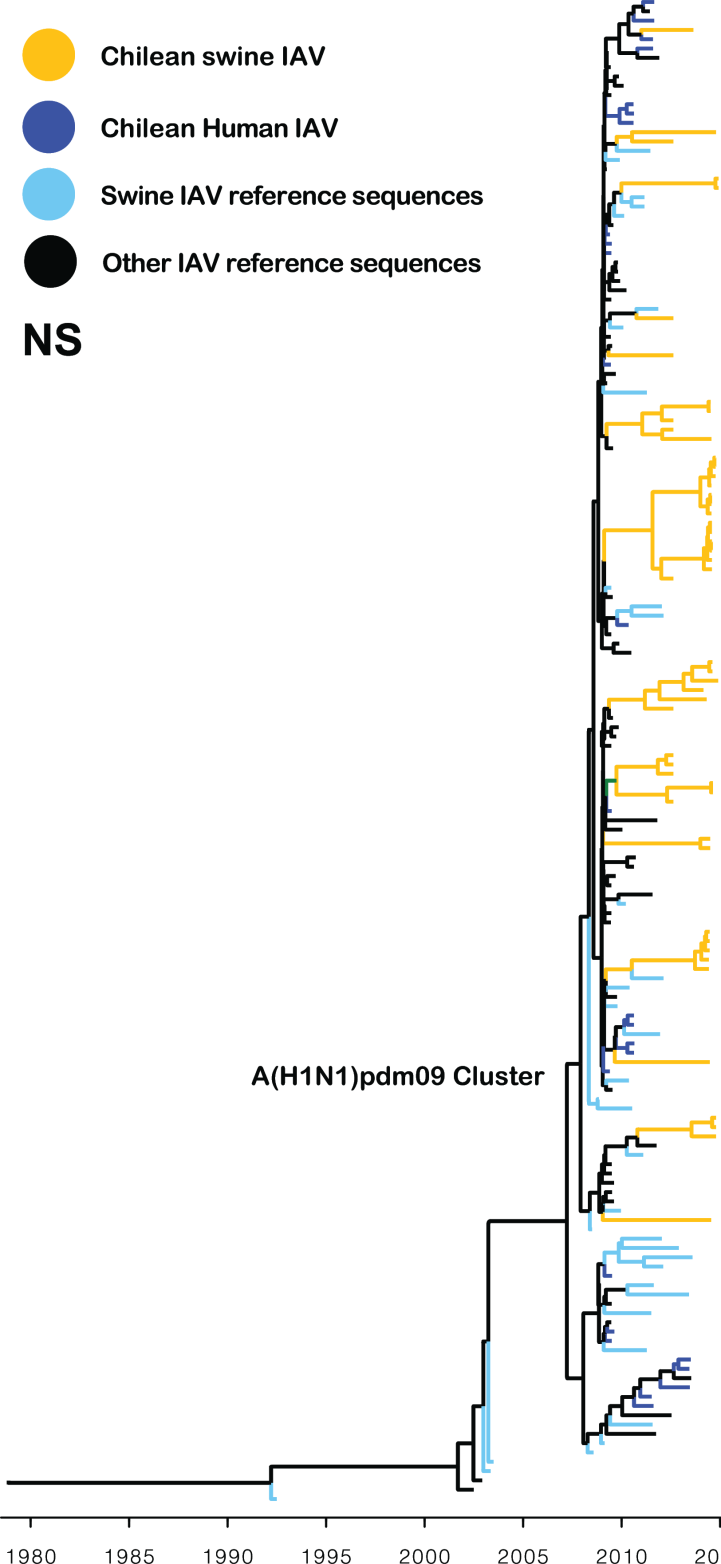
The NS A(H1N1)pdm09 gene has been introduced multiple times in the swine population. Maximum clade credibility tree depicting TMRCA estimates of NS gene segment of human and swine IAVs. All Chilean swine NS sequences belong to the A(H1N1)pdm09-like lineage. Multiple introductions of the NS pandemic gene have been introduced into the swine population since the 2009 H1N1 pandemic.

All the IAV isolates contained internal segments derived from the A(H1N1)pdm09 virus. Interestingly, 49 out of 52 matrix (M) gene sequences were grouped into a monophyletic cluster (TMRCA May 2010, 95% HPD April 2009–March 2011), which is related to early human A(H1N1)pdm09 strains isolated in Chile in 2009 (Fig. 4). Similarly, the phylogeny of the PA and NP genes showed that most of the sequences were grouped into one to three major clusters, which are related to human A(H1N1)pdm09 strains from 2009 and 2010 (Fig. S1). This suggests that highly fitted M, PA, and NP segments were introduced into the swine population early after the 2009 pandemic outbreak in Chile and selectively maintained in the swine population since then. In contrast, the phylogeny of the NS, PB1, and PB2 genes showed a higher diversity and multiple introductions (Fig. 5; Fig. S1), with no clear advantage of a particular viral introduction.

### Multiple reassortant swine IAVs circulating in Chile

Overall, eight swine IAV genotypes were identified from the 52 whole-genome sequences. As mentioned above, all genotypes have A(H1N1)pdm09-like internal genes, differing in the lineages of the genes encoding HA and NA. The most prevalent subtypes were H1N2 (26/52) and H1N1 (24/52). In the case of the subtype H1N2, the most prevalent genotypes were those with the association of the lineages ChH1A with ChN2 (20/26) and ChH1B with ChN2 (3/26), which were named ChH1N2A and ChH1N2B, respectively. In the case of subtype H1N1, the most prevalent genotype was the A(H1N1)pdm09 like (18/24). Only two IAV isolates belonged to the subtype H3N2. The HA and NA lineages of ChH1N2A, ChH1N2B, and H3N2 swine IAVs are divergent from all other known IAVs, therefore these genotypes are unique in the world.

### Chilean swine IAVs are genetically distant from contemporary human viruses, and the general population has limited cross-protective antibodies against them

We performed phylogenetic analyses to determine the relationship of newly identified swine IAVs with contemporary human seasonal viruses and with the human vaccine strains recommended by the World Health Organization. We used an extensive list of human viruses isolated from 1918 to 2015 and the vaccine strains of the last 40 years. These analyses confirmed that the lineages ChH1A, ChH1B, swine H3, and ChN2 are genetically distant from other human and swine IAVs described worldwide. Importantly, these novel swine H1N2 and H3N2 IAVs were clustered distantly from all previously recommended human vaccine strains (Fig. S2A through C).

**Fig 6:**
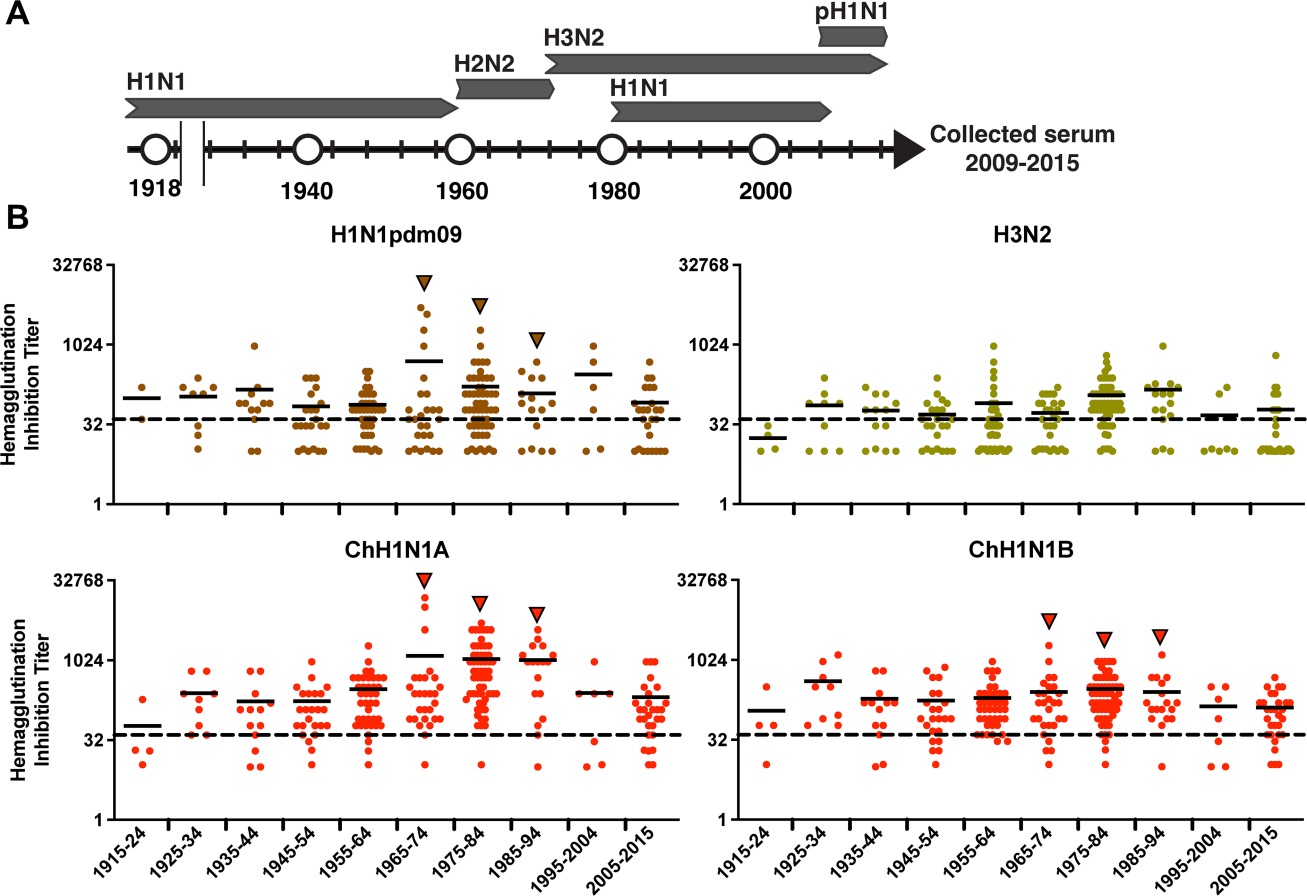
Serological protection of humans of different ages against the Chilean swine IAVs. (**A**) Influenza A strains that circulated in human populations since 1918. H1N1: 1918–1957 and 1977–2009; H2N2: 1957–1968; H3N2: 1968–to date: A(H1N1)pdm09: 2009–to date. (**B**) 237 human sera collected from 2009 to 2015 were evaluated against the Chilean swine viruses by hemagglutination inhibition assay. Serum titers against the A(H1N1)pdm09, H3N2, ChH1N2A, and ChH1N2B swine viruses are grouped and shown according to individual’s date of birth.

Then, we evaluated if humans had cross-reactive antibodies against these novel swine IAVs. Proper evaluation of cross-protective immunity involves the assessment of the local populations that could be exposed to these viruses. Hence, we used 237 human sera collected from an influenza clinical cohort study carried out in Chile between 2009 and 2015. These individuals were born between 1915 and 2015, covering the entire time frame of potential exposure to human influenza viruses of the last 100 years (Fig. 6). We observed a moderate to high level of neutralizing antibodies against the ChH1N2A, ChH1N2B, and swine A(H1N1)pdm09-like viruses in middle-aged individuals (date of birth: 1965–94). In contrast, lower reactivity was observed in the youngest and oldest individuals, born in 1995–2015 and 1915–1944, respectively (individuals <20 and >70 years of age). This pattern of “age-related protection” was not observed for the oldest swine H3N2 virus, where similar low to mid-levels of antibody reactivity were seen in individuals of all ages, suggesting that this virus may pose a higher zoonotic potential risk (Fig. 6).

## DISCUSSION

This is the first in-depth study on the genetic diversity of novel swine IAVs recently identified in Chile, determining their likely origin, introduction times, and evolution. In addition, we evaluated the zoonotic potential of these viruses, determining the level of cross-protective antibodies in the Chilean human population.

Our results indicate that swine IAVs in Chile have a gene constellation originating from several introductions of human IAVs into the swine population and multiple reassortment events over a period of ~30 years. The introduction of the human HA and NA genes took place as early as the 1990s. Today, these swine H1N2 and H3N2 IAVs are genetically distant from all other IAVs described worldwide, including human and swine vaccine strains (9). The phylogenies of both gene segments showed long branch lengths (Fig. 1 to 3) without any genetically related intermediate viruses, which is likely due to a lack of swine IAV genomic surveillance data prior to 2012. These results suggest that these viruses have undergone substantial evolution and drift in swine populations (they are not derived from more recent human introductions).

Due to the lack of IAV surveillance in swine in Chile before 2012, it is not possible to determine the genotypes and HA and NA lineages that prevailed in the swine population in the past. However, based on our analyses, it is plausible that human seasonal H1N1 and H3N2 IAVs were introduced in swine between the late 1980s and early 1990s, generating the current H1N2 lineages through reassortment rather than the direct introduction of human H1N2 viruses.

The internal gene segments of all swine IAV genotypes identified in this study belonged to the A(H1N1)pdm09-like lineage. Reassortment of internal A(H1N1)pdm09-like genes resulting in IAVs with either a mixture of pandemic or endemic glycoproteins has also been reported in other countries (25–29). A prevalent reassortant Eurasian avian-like H1N1 virus was recently identified in China, which contains some A(H1N1)pdm09-like internal genes (PB2, PB1, PA, NP, and M) that have increased its human infectivity (30). Nonetheless, the complete displacement of previous internal genes by A(H1N1)pdm09-like genes in all IAV genotypes detected in a surveillance study is highly unusual and has not been reported in other regions. For example, in North America, Europe, and Asia, classical or avian-like lineages that existed prior to 2009 are still prevalent (27, 28, 31–33). Interestingly, in contrast to other internal genes that show evidence of multiple A(H1N1)pdm09 IAV introductions, the sequences of the M segment were grouped in a major monophyletic cluster (with the exception of three sequences), regardless of the genotype to which it belonged. Previous studies have suggested that the A(H1N1)pdm09 M segment increases the transmissibility of IAVs (34, 35). Hence, the predominance of this specific M gene, derived from an early A(H1N1)pdm09 human virus, suggests that this gene could have increased the replication and transmission fitness of IAV in swine. This is highly relevant from a public health perspective since swine H3N2 variants containing the A(H1N1)pdm09 M segment have been responsible for zoonotic transmission in the USA (36, 37).

**Fig 7:**
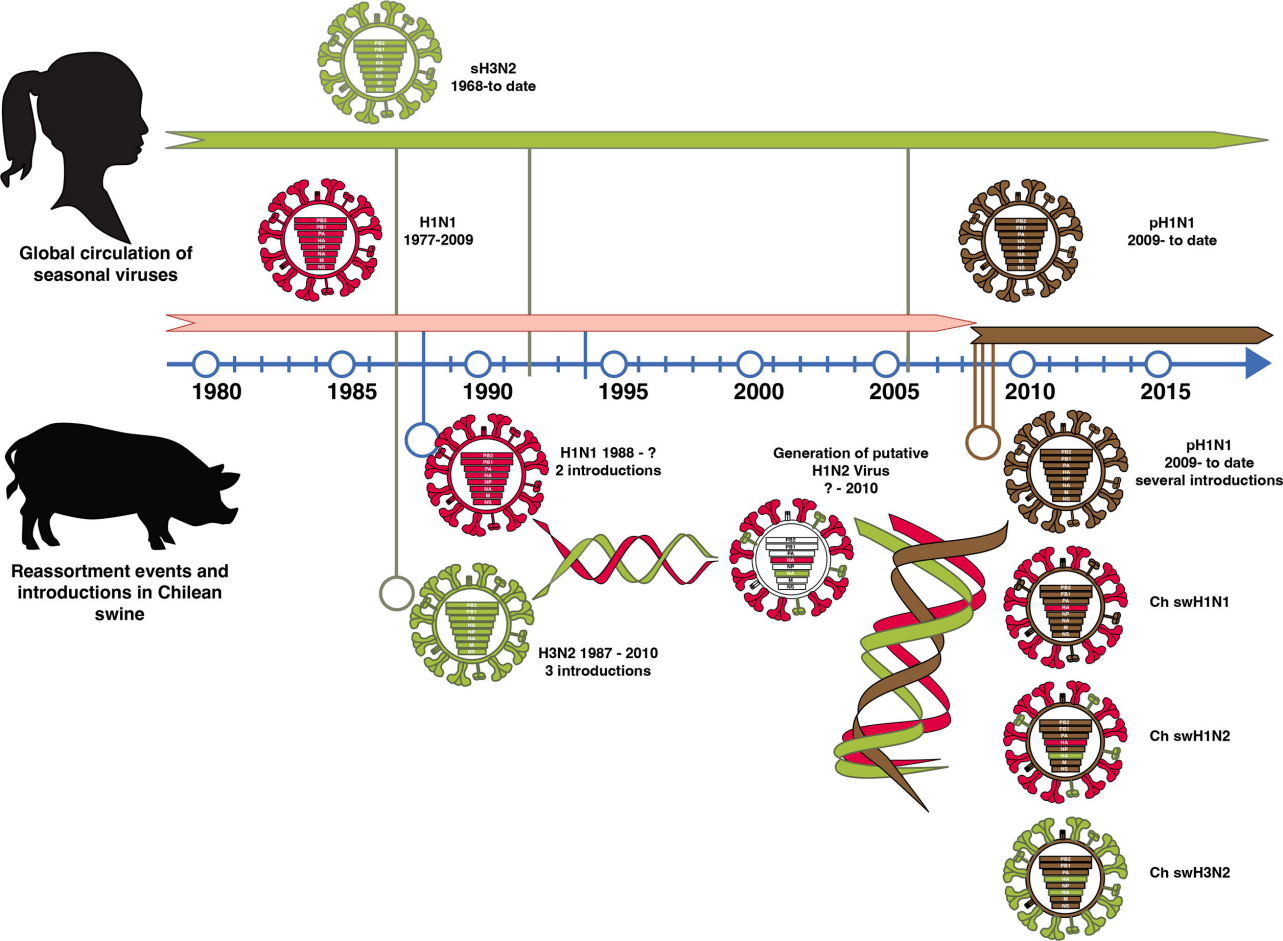
Model of the introduction of human influenza viruses into swine in Chile leading to the generation of novel swine IAVs. Human H1N1 and H3N2 seasonal viruses were introduced into swine as early as the mid to late 1980s. The H1N1 were introduced at two time points during this decade, whereas the H3N2 subtype was introduced in the early 1990s and then again sometime after 2005. This likely generated double reassortment events that gave rise to the ChH1N2A and ChH1N2B lineages and to the rare H3N2 viruses found in Chile. Upon the emergence of the 2009 H1N1 human pandemic, this strain was rapidly introduced into the Chilean swine population early during the pandemic and thereafter in multiple occasions generating multiple additional reassortment events that gave rise to the increased diversity of genotypes found in the country. In all cases, all the viruses have six internal genes belonging to the A(H1N1)pdm09 strain, which replaced the previously exiting internal genes (currently of unknown origin).

Based on these phylogenetic analyses and their results, we proposed a model of the introduction of human influenza viruses into the swine population in Chile leading to the generation of novel swine IAVs, considering potential introduction times and evolution through multiple reassortment events (Fig. 7).

All these findings prompted us to conduct a serological analysis to determine the level of cross-protective antibodies in the Chilean human population. This serological analysis indicated that an important proportion of the population is likely to be susceptible to the swine H3N2 strain and that there is variable protection against the ChH1N2A, ChH1N2B, and swine A(H1N1)pdm09-like viruses, depending on age, where young and old people would be more susceptible. In general, individuals showing the highest antibody titers were born during the period of circulation of the human IAV strains from which these swine IAVs are derived (Fig. 6). This supports the concept that the first encounter with the virus has a strong effect on the antibody imprinting of individuals (38) and highlights the notion that, with the passing of time, new naïve individuals become susceptible to these IAVs maintained in swine. In addition, a previous study reported that these Chilean swine H1 IAVs have unique glycosylation site patterns, not detected in human IAV strains, and identified the emergence of some antigenic variants within the clusters CH1A and A(H1N1)pdm09 like, which may lead to immune evasion (9).

In summary, we showed that Chilean swine H1N2 and H3N2 viruses likely originated from human seasonal viruses introduced to the swine population multiple times since the mid-1980s. Our results also show that the introduction of human A(H1N1)pdm09 viruses into swine populations generated a series of additional reassortment events that further diversified the swine IAV genotypes and completely displaced the internal genes of the pre-pandemic swine IAV endemic stains. Overall, three to four putative reassortment patterns generated eight distinct swine IAV genotypes that circulate in the Chilean swine population today (Fig. 7), which differ from other swine and human IAVs previously reported worldwide. Importantly, our results indicate that the general population is susceptible to the swine H3N2 viruses and that the elderly and young children (<10 years of age) also lack protective antibodies against the swine H1N2 strains, suggesting that these viruses could be potential zoonotic threats.

To date, this is the largest molecular epidemiological study of swine IAV in South America, contributing to global IAV studies in both humans and animals. Continuous surveillance of IAVs circulating in Chilean swine populations and monitoring of workers in the swine industry are necessary and strongly recommended.

## Data Availability

The genome sequence data for all strains determined in this study have been deposited in the GenBank database and are publicly available. The accession numbers are incorporated in the supplemental table.

## SUPPLEMENTAL MATERIAL

Supplemental material spectrum.02181-23-s0001.docx Supplemental figures and table.

ASM does not own the copyrights to Supplemental Material that may be linked to, or accessed through, an article. The authors have granted ASM a non-exclusive, world-wide license to publish the Supplemental Material files. Please contact the corresponding author directly for reuse.
