# Supplementary material for: Novel influenza A viruses in pigs with zoonotic potential, Chile
Source: Microbiol Spectr. 2024 Mar 7;12(4):e02181-23. doi: 10.1128/spectrum.02181-23 (PMC10986610; doi:10.1128/spectrum.02181-23)
Supplement: Supplemental material — Supplemental figures and table. [file spectrum.02181-23-s0001.docx]

**Supplemental Figure 1.** Internal genes revealed multiple introductions of A(H1N1)pdm09 IAV into the Chilean swine population. Phylogenetic relationships of the PB2, PB1, PA, and NP gene segments. Maximum clade credibility tree reconstructed with influenza viruses collected from Chilean humans and swine. The bottom axis denotes years as dated in the analyses.

**
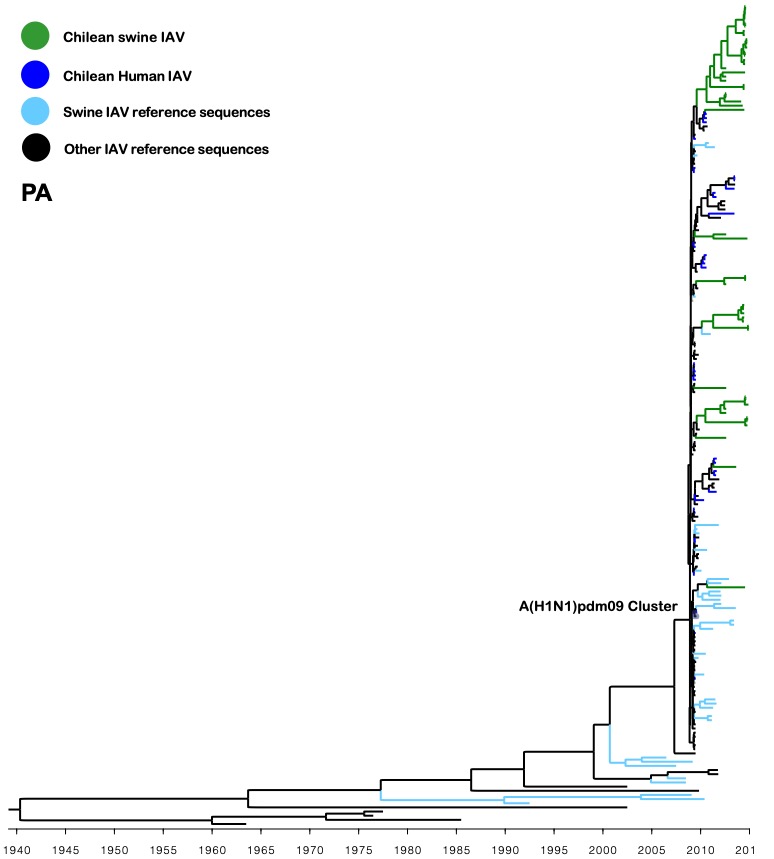
**

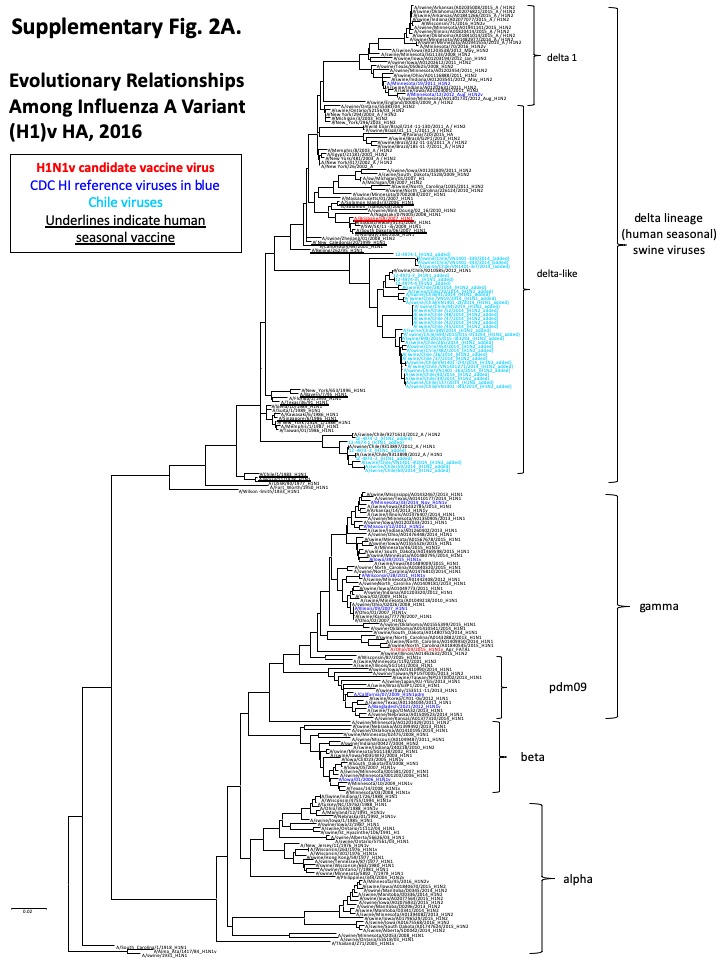
Supplementary Figure 2. Evolutionary distant relationships among Chilean swine IAV variants and human vaccine strains. Maximum likelihood tree of the HA gene of H1 (A), H3 (B), and N2 (C) subtypes. Chilean swine IAVs are in cyan, variant candidate vaccine viruses are in red, and reference viruses for hemagglutinin inhibition assay by CDC are in blue. Human seasonal vaccine strains are underlined. The scale bar represents nucleotide substitutions per site.

**
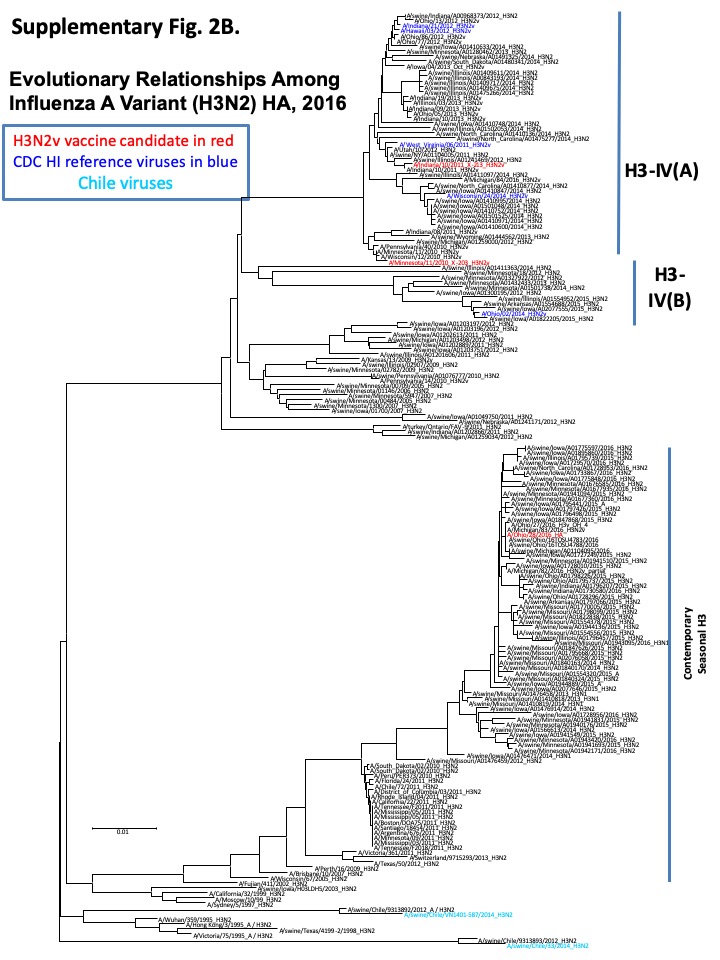
**

**
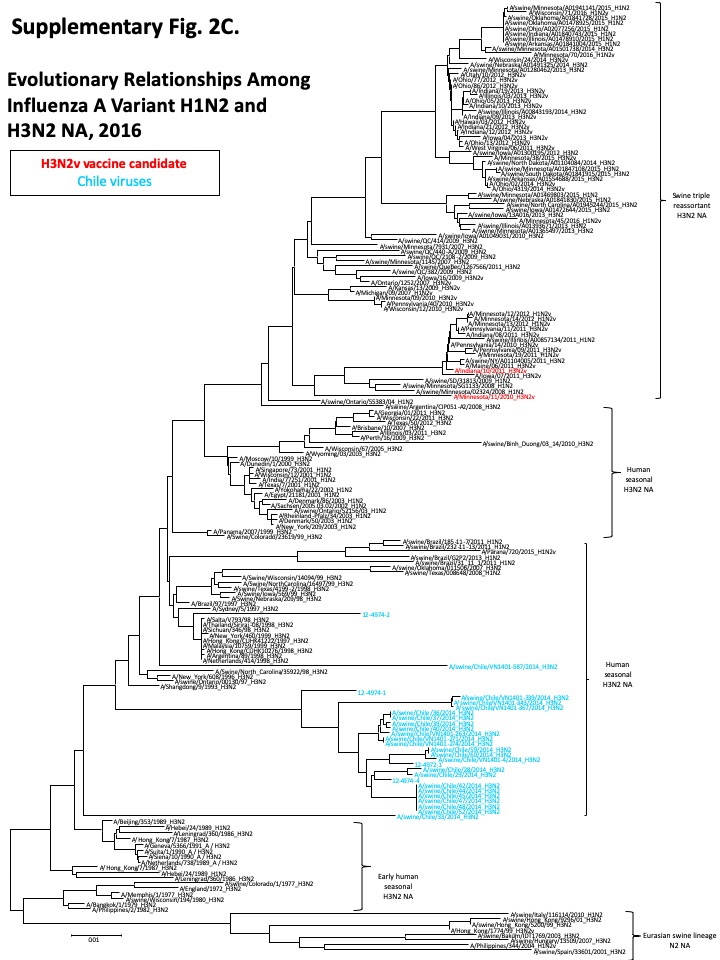
**

Supplementary Table. Accession numbers

| Accession | Segment | Subtype | Date | Name strain |
| --- | --- | --- | --- | --- |
| MF099003 | 6 (NA) | H1N1 | 19-06-14 | (A/swine/Chile/10/2014(H1N1)) |
| MF099017 | 5 (NP) | H1N1 | 19-06-14 | (A/swine/Chile/10/2014(H1N1)) |
| MF099108 | 3 (PA) | H1N1 | 19-06-14 | (A/swine/Chile/10/2014(H1N1)) |
| MF099131 | 2 (PB1) | H1N1 | 19-06-14 | (A/swine/Chile/10/2014(H1N1)) |
| MF099148 | 4 (HA) | H1N1 | 19-06-14 | (A/swine/Chile/10/2014(H1N1)) |
| MF099243 | 8 (NS) | H1N1 | 19-06-14 | (A/swine/Chile/10/2014(H1N1)) |
| MF099273 | 1 (PB2) | H1N1 | 19-06-14 | (A/swine/Chile/10/2014(H1N1)) |
| MF099354 | 7 (MP) | H1N1 | 19-06-14 | (A/swine/Chile/10/2014(H1N1)) |
| MF098868 | 8 (NS) | H1N1 | 19-06-14 | (A/swine/Chile/12/2014(H1N1)) |
| MF098882 | 5 (NP) | H1N1 | 19-06-14 | (A/swine/Chile/12/2014(H1N1)) |
| MF098885 | 4 (HA) | H1N1 | 19-06-14 | (A/swine/Chile/12/2014(H1N1)) |
| MF098926 | 1 (PB2) | H1N1 | 19-06-14 | (A/swine/Chile/12/2014(H1N1)) |
| MF098945 | 6 (NA) | H1N1 | 19-06-14 | (A/swine/Chile/12/2014(H1N1)) |
| MF099186 | 3 (PA) | H1N1 | 19-06-14 | (A/swine/Chile/12/2014(H1N1)) |
| MF099237 | 7 (MP) | H1N1 | 19-06-14 | (A/swine/Chile/12/2014(H1N1)) |
| MF099290 | 2 (PB1) | H1N1 | 19-06-14 | (A/swine/Chile/12/2014(H1N1)) |
| MF098871 | 5 (NP) | H1N1 | 19-06-14 | (A/swine/Chile/14/2014(H1N1)) |
| MF098967 | 4 (HA) | H1N1 | 19-06-14 | (A/swine/Chile/14/2014(H1N1)) |
| MF098973 | 2 (PB1) | H1N1 | 19-06-14 | (A/swine/Chile/14/2014(H1N1)) |
| MF099018 | 6 (NA) | H1N1 | 19-06-14 | (A/swine/Chile/14/2014(H1N1)) |
| MF099104 | 8 (NS) | H1N1 | 19-06-14 | (A/swine/Chile/14/2014(H1N1)) |
| MF099150 | 7 (MP) | H1N1 | 19-06-14 | (A/swine/Chile/14/2014(H1N1)) |
| MF099278 | 1 (PB2) | H1N1 | 19-06-14 | (A/swine/Chile/14/2014(H1N1)) |
| MF099349 | 3 (PA) | H1N1 | 19-06-14 | (A/swine/Chile/14/2014(H1N1)) |
| MF098800 | 7 (MP) | H1N1 | 23-06-14 | (A/swine/Chile/15/2014(H1N1)) |
| MF098983 | 8 (NS) | H1N1 | 23-06-14 | (A/swine/Chile/15/2014(H1N1)) |
| MF098994 | 3 (PA) | H1N1 | 23-06-14 | (A/swine/Chile/15/2014(H1N1)) |
| MF099196 | 2 (PB1) | H1N1 | 23-06-14 | (A/swine/Chile/15/2014(H1N1)) |
| MF099275 | 1 (PB2) | H1N1 | 23-06-14 | (A/swine/Chile/15/2014(H1N1)) |
| MF099299 | 5 (NP) | H1N1 | 23-06-14 | (A/swine/Chile/15/2014(H1N1)) |
| MF099346 | 6 (NA) | H1N1 | 23-06-14 | (A/swine/Chile/15/2014(H1N1)) |
| MF099397 | 4 (HA) | H1N1 | 23-06-14 | (A/swine/Chile/15/2014(H1N1)) |
| MF098933 | 3 (PA) | H1N1 | 29-06-14 | (A/swine/Chile/18/2014(H1N1)) |
| MF099036 | 7 (MP) | H1N1 | 29-06-14 | (A/swine/Chile/18/2014(H1N1)) |
| MF099126 | 6 (NA) | H1N1 | 29-06-14 | (A/swine/Chile/18/2014(H1N1)) |
| MF099250 | 4 (HA) | H1N1 | 29-06-14 | (A/swine/Chile/18/2014(H1N1)) |
| MF099267 | 5 (NP) | H1N1 | 29-06-14 | (A/swine/Chile/18/2014(H1N1)) |
| MF099292 | 1 (PB2) | H1N1 | 29-06-14 | (A/swine/Chile/18/2014(H1N1)) |
| MF099333 | 2 (PB1) | H1N1 | 29-06-14 | (A/swine/Chile/18/2014(H1N1)) |
| MF099398 | 8 (NS) | H1N1 | 29-06-14 | (A/swine/Chile/18/2014(H1N1)) |
| MF098828 | 5 (NP) | H1N1 | 23-08-14 | (A/swine/Chile/2-7/2014(H1N1)) |
| MF098845 | 1 (PB2) | H1N1 | 23-08-14 | (A/swine/Chile/2-7/2014(H1N1)) |
| MF098853 | 3 (PA) | H1N1 | 23-08-14 | (A/swine/Chile/2-7/2014(H1N1)) |
| MF098990 | 8 (NS) | H1N1 | 23-08-14 | (A/swine/Chile/2-7/2014(H1N1)) |
| MF099061 | 2 (PB1) | H1N1 | 23-08-14 | (A/swine/Chile/2-7/2014(H1N1)) |
| MF099099 | 4 (HA) | H1N1 | 23-08-14 | (A/swine/Chile/2-7/2014(H1N1)) |
| MF099100 | 7 (MP) | H1N1 | 23-08-14 | (A/swine/Chile/2-7/2014(H1N1)) |
| MF099188 | 6 (NA) | H1N1 | 23-08-14 | (A/swine/Chile/2-7/2014(H1N1)) |
| MF098832 | 1 (PB2) | H1N1 | 29-06-14 | (A/swine/Chile/20/2014(H1N1)) |
| MF098883 | 2 (PB1) | H1N1 | 29-06-14 | (A/swine/Chile/20/2014(H1N1)) |
| MF098931 | 6 (NA) | H1N1 | 29-06-14 | (A/swine/Chile/20/2014(H1N1)) |
| MF098943 | 7 (MP) | H1N1 | 29-06-14 | (A/swine/Chile/20/2014(H1N1)) |
| MF099041 | 8 (NS) | H1N1 | 29-06-14 | (A/swine/Chile/20/2014(H1N1)) |
| MF099064 | 4 (HA) | H1N1 | 29-06-14 | (A/swine/Chile/20/2014(H1N1)) |
| MF099076 | 5 (NP) | H1N1 | 29-06-14 | (A/swine/Chile/20/2014(H1N1)) |
| MF099170 | 3 (PA) | H1N1 | 29-06-14 | (A/swine/Chile/20/2014(H1N1)) |
| MF098979 | 3 (PA) | H1N2 | 08-07-14 | (A/swine/Chile/28/2014(H1N2)) |
| MF098998 | 6 (NA) | H1N2 | 08-07-14 | (A/swine/Chile/28/2014(H1N2)) |
| MF099014 | 1 (PB2) | H1N2 | 08-07-14 | (A/swine/Chile/28/2014(H1N2)) |
| MF099049 | 2 (PB1) | H1N2 | 08-07-14 | (A/swine/Chile/28/2014(H1N2)) |
| MF099083 | 4 (HA) | H1N2 | 08-07-14 | (A/swine/Chile/28/2014(H1N2)) |
| MF099256 | 7 (MP) | H1N2 | 08-07-14 | (A/swine/Chile/28/2014(H1N2)) |
| MF099334 | 5 (NP) | H1N2 | 08-07-14 | (A/swine/Chile/28/2014(H1N2)) |
| MF099404 | 8 (NS) | H1N2 | 08-07-14 | (A/swine/Chile/28/2014(H1N2)) |
| MF098815 | 7 (MP) | H1N2 | 08-07-14 | (A/swine/Chile/29/2014(H1N2)) |
| MF098835 | 1 (PB2) | H1N2 | 08-07-14 | (A/swine/Chile/29/2014(H1N2)) |
| MF098935 | 6 (NA) | H1N2 | 08-07-14 | (A/swine/Chile/29/2014(H1N2)) |
| MF099039 | 2 (PB1) | H1N2 | 08-07-14 | (A/swine/Chile/29/2014(H1N2)) |
| MF099071 | 4 (HA) | H1N2 | 08-07-14 | (A/swine/Chile/29/2014(H1N2)) |
| MF099163 | 8 (NS) | H1N2 | 08-07-14 | (A/swine/Chile/29/2014(H1N2)) |
| MF099184 | 3 (PA) | H1N2 | 08-07-14 | (A/swine/Chile/29/2014(H1N2)) |
| MF099342 | 5 (NP) | H1N2 | 08-07-14 | (A/swine/Chile/29/2014(H1N2)) |
| MF098837 | 6 (NA) | H3N2 | 21-07-14 | (A/swine/Chile/33/2014(H3N2)) |
| MF099010 | 3 (PA) | H3N2 | 21-07-14 | (A/swine/Chile/33/2014(H3N2)) |
| MF099023 | 1 (PB2) | H3N2 | 21-07-14 | (A/swine/Chile/33/2014(H3N2)) |
| MF099048 | 2 (PB1) | H3N2 | 21-07-14 | (A/swine/Chile/33/2014(H3N2)) |
| MF099075 | 4 (HA) | H3N2 | 21-07-14 | (A/swine/Chile/33/2014(H3N2)) |
| MF099103 | 7 (MP) | H3N2 | 21-07-14 | (A/swine/Chile/33/2014(H3N2)) |
| MF099137 | 8 (NS) | H3N2 | 21-07-14 | (A/swine/Chile/33/2014(H3N2)) |
| MF099339 | 5 (NP) | H3N2 | 21-07-14 | (A/swine/Chile/33/2014(H3N2)) |
| MF098870 | 8 (NS) | H1N2 | 22-07-14 | (A/swine/Chile/36/2014(H1N2)) |
| MF098907 | 2 (PB1) | H1N2 | 22-07-14 | (A/swine/Chile/36/2014(H1N2)) |
| MF098923 | 1 (PB2) | H1N2 | 22-07-14 | (A/swine/Chile/36/2014(H1N2)) |
| MF098982 | 7 (MP) | H1N2 | 22-07-14 | (A/swine/Chile/36/2014(H1N2)) |
| MF099121 | 4 (HA) | H1N2 | 22-07-14 | (A/swine/Chile/36/2014(H1N2)) |
| MF099144 | 5 (NP) | H1N2 | 22-07-14 | (A/swine/Chile/36/2014(H1N2)) |
| MF099180 | 3 (PA) | H1N2 | 22-07-14 | (A/swine/Chile/36/2014(H1N2)) |
| MF099338 | 6 (NA) | H1N2 | 22-07-14 | (A/swine/Chile/36/2014(H1N2)) |
| MF098803 | 7 (MP) | H1N2 | 22-07-14 | (A/swine/Chile/37/2014(H1N2)) |
| MF098817 | 3 (PA) | H1N2 | 22-07-14 | (A/swine/Chile/37/2014(H1N2)) |
| MF098859 | 2 (PB1) | H1N2 | 22-07-14 | (A/swine/Chile/37/2014(H1N2)) |
| MF098864 | 8 (NS) | H1N2 | 22-07-14 | (A/swine/Chile/37/2014(H1N2)) |
| MF098963 | 5 (NP) | H1N2 | 22-07-14 | (A/swine/Chile/37/2014(H1N2)) |
| MF099059 | 6 (NA) | H1N2 | 22-07-14 | (A/swine/Chile/37/2014(H1N2)) |
| MF099318 | 4 (HA) | H1N2 | 22-07-14 | (A/swine/Chile/37/2014(H1N2)) |
| MF099387 | 1 (PB2) | H1N2 | 22-07-14 | (A/swine/Chile/37/2014(H1N2)) |
| MF098805 | 7 (MP) | H1N2 | 22-07-14 | (A/swine/Chile/39/2014(H1N2)) |
| MF098808 | 8 (NS) | H1N2 | 22-07-14 | (A/swine/Chile/39/2014(H1N2)) |
| MF098858 | 2 (PB1) | H1N2 | 22-07-14 | (A/swine/Chile/39/2014(H1N2)) |
| MF098996 | 6 (NA) | H1N2 | 22-07-14 | (A/swine/Chile/39/2014(H1N2)) |
| MF099223 | 1 (PB2) | H1N2 | 22-07-14 | (A/swine/Chile/39/2014(H1N2)) |
| MF099335 | 3 (PA) | H1N2 | 22-07-14 | (A/swine/Chile/39/2014(H1N2)) |
| MF099364 | 4 (HA) | H1N2 | 22-07-14 | (A/swine/Chile/39/2014(H1N2)) |
| MF099376 | 5 (NP) | H1N2 | 22-07-14 | (A/swine/Chile/39/2014(H1N2)) |
| MF099106 | 4 (HA) | H1N2 | 22-07-14 | (A/swine/Chile/40/2014(H1N2)) |
| MF099136 | 7 (MP) | H1N2 | 22-07-14 | (A/swine/Chile/40/2014(H1N2)) |
| MF099174 | 1 (PB2) | H1N2 | 22-07-14 | (A/swine/Chile/40/2014(H1N2)) |
| MF099187 | 6 (NA) | H1N2 | 22-07-14 | (A/swine/Chile/40/2014(H1N2)) |
| MF099204 | 2 (PB1) | H1N2 | 22-07-14 | (A/swine/Chile/40/2014(H1N2)) |
| MF099210 | 8 (NS) | H1N2 | 22-07-14 | (A/swine/Chile/40/2014(H1N2)) |
| MF099388 | 5 (NP) | H1N2 | 22-07-14 | (A/swine/Chile/40/2014(H1N2)) |
| MF099401 | 3 (PA) | H1N2 | 22-07-14 | (A/swine/Chile/40/2014(H1N2)) |
| MF098825 | 4 (HA) | H1N1 | 22-07-14 | (A/swine/Chile/41/2014(H1N1)) |
| MF098880 | 8 (NS) | H1N1 | 22-07-14 | (A/swine/Chile/41/2014(H1N1)) |
| MF098903 | 6 (NA) | H1N1 | 22-07-14 | (A/swine/Chile/41/2014(H1N1)) |
| MF098936 | 7 (MP) | H1N1 | 22-07-14 | (A/swine/Chile/41/2014(H1N1)) |
| MF098940 | 1 (PB2) | H1N1 | 22-07-14 | (A/swine/Chile/41/2014(H1N1)) |
| MF099052 | 2 (PB1) | H1N1 | 22-07-14 | (A/swine/Chile/41/2014(H1N1)) |
| MF099088 | 5 (NP) | H1N1 | 22-07-14 | (A/swine/Chile/41/2014(H1N1)) |
| MF099218 | 3 (PA) | H1N1 | 22-07-14 | (A/swine/Chile/41/2014(H1N1)) |
| MF098927 | 6 (NA) | H1N2 | 31-07-14 | (A/swine/Chile/42/2014(H1N2)) |
| MF098952 | 1 (PB2) | H1N2 | 31-07-14 | (A/swine/Chile/42/2014(H1N2)) |
| MF099056 | 8 (NS) | H1N2 | 31-07-14 | (A/swine/Chile/42/2014(H1N2)) |
| MF099102 | 3 (PA) | H1N2 | 31-07-14 | (A/swine/Chile/42/2014(H1N2)) |
| MF099139 | 5 (NP) | H1N2 | 31-07-14 | (A/swine/Chile/42/2014(H1N2)) |
| MF099222 | 7 (MP) | H1N2 | 31-07-14 | (A/swine/Chile/42/2014(H1N2)) |
| MF099244 | 2 (PB1) | H1N2 | 31-07-14 | (A/swine/Chile/42/2014(H1N2)) |
| MF099312 | 4 (HA) | H1N2 | 31-07-14 | (A/swine/Chile/42/2014(H1N2)) |
| MF098867 | 8 (NS) | H1N2 | 31-07-14 | (A/swine/Chile/44/2014(H1N2)) |
| MF098949 | 1 (PB2) | H1N2 | 31-07-14 | (A/swine/Chile/44/2014(H1N2)) |
| MF098961 | 3 (PA) | H1N2 | 31-07-14 | (A/swine/Chile/44/2014(H1N2)) |
| MF099089 | 5 (NP) | H1N2 | 31-07-14 | (A/swine/Chile/44/2014(H1N2)) |
| MF099123 | 2 (PB1) | H1N2 | 31-07-14 | (A/swine/Chile/44/2014(H1N2)) |
| MF099226 | 6 (NA) | H1N2 | 31-07-14 | (A/swine/Chile/44/2014(H1N2)) |
| MF099231 | 7 (MP) | H1N2 | 31-07-14 | (A/swine/Chile/44/2014(H1N2)) |
| MF099265 | 4 (HA) | H1N2 | 31-07-14 | (A/swine/Chile/44/2014(H1N2)) |
| MF098956 | 6 (NA) | H1N2 | 31-07-14 | (A/swine/Chile/45/2014(H1N2)) |
| MF099021 | 5 (NP) | H1N2 | 31-07-14 | (A/swine/Chile/45/2014(H1N2)) |
| MF099030 | 7 (MP) | H1N2 | 31-07-14 | (A/swine/Chile/45/2014(H1N2)) |
| MF099063 | 1 (PB2) | H1N2 | 31-07-14 | (A/swine/Chile/45/2014(H1N2)) |
| MF099086 | 2 (PB1) | H1N2 | 31-07-14 | (A/swine/Chile/45/2014(H1N2)) |
| MF099092 | 4 (HA) | H1N2 | 31-07-14 | (A/swine/Chile/45/2014(H1N2)) |
| MF099285 | 3 (PA) | H1N2 | 31-07-14 | (A/swine/Chile/45/2014(H1N2)) |
| MF099356 | 8 (NS) | H1N2 | 31-07-14 | (A/swine/Chile/45/2014(H1N2)) |
| MF098816 | 5 (NP) | H1N2 | 31-07-14 | (A/swine/Chile/47/2014(H1N2)) |
| MF098965 | 4 (HA) | H1N2 | 31-07-14 | (A/swine/Chile/47/2014(H1N2)) |
| MF098976 | 3 (PA) | H1N2 | 31-07-14 | (A/swine/Chile/47/2014(H1N2)) |
| MF099227 | 1 (PB2) | H1N2 | 31-07-14 | (A/swine/Chile/47/2014(H1N2)) |
| MF099300 | 8 (NS) | H1N2 | 31-07-14 | (A/swine/Chile/47/2014(H1N2)) |
| MF099326 | 6 (NA) | H1N2 | 31-07-14 | (A/swine/Chile/47/2014(H1N2)) |
| MF099353 | 7 (MP) | H1N2 | 31-07-14 | (A/swine/Chile/47/2014(H1N2)) |
| MF099362 | 2 (PB1) | H1N2 | 31-07-14 | (A/swine/Chile/47/2014(H1N2)) |
| MF098860 | 5 (NP) | H1N2 | 31-07-14 | (A/swine/Chile/48/2014(H1N2)) |
| MF098879 | 3 (PA) | H1N2 | 31-07-14 | (A/swine/Chile/48/2014(H1N2)) |
| MF098890 | 2 (PB1) | H1N2 | 31-07-14 | (A/swine/Chile/48/2014(H1N2)) |
| MF099037 | 4 (HA) | H1N2 | 31-07-14 | (A/swine/Chile/48/2014(H1N2)) |
| MF099054 | 6 (NA) | H1N2 | 31-07-14 | (A/swine/Chile/48/2014(H1N2)) |
| MF099154 | 8 (NS) | H1N2 | 31-07-14 | (A/swine/Chile/48/2014(H1N2)) |
| MF099211 | 1 (PB2) | H1N2 | 31-07-14 | (A/swine/Chile/48/2014(H1N2)) |
| MF099215 | 7 (MP) | H1N2 | 31-07-14 | (A/swine/Chile/48/2014(H1N2)) |
| MF098915 | 4 (HA) | H1N2 | 31-07-14 | (A/swine/Chile/52/2014(H1N2)) |
| MF098985 | 1 (PB2) | H1N2 | 31-07-14 | (A/swine/Chile/52/2014(H1N2)) |
| MF099026 | 7 (MP) | H1N2 | 31-07-14 | (A/swine/Chile/52/2014(H1N2)) |
| MF099078 | 2 (PB1) | H1N2 | 31-07-14 | (A/swine/Chile/52/2014(H1N2)) |
| MF099178 | 6 (NA) | H1N2 | 31-07-14 | (A/swine/Chile/52/2014(H1N2)) |
| MF099202 | 3 (PA) | H1N2 | 31-07-14 | (A/swine/Chile/52/2014(H1N2)) |
| MF099283 | 5 (NP) | H1N2 | 31-07-14 | (A/swine/Chile/52/2014(H1N2)) |
| MF099351 | 8 (NS) | H1N2 | 31-07-14 | (A/swine/Chile/52/2014(H1N2)) |
| MF099232 | 2 (PB1) | H1N2 | 10-08-14 | (A/swine/Chile/59/2014(H1N2)) |
| MF099233 | 4 (HA) | H1N2 | 10-08-14 | (A/swine/Chile/59/2014(H1N2)) |
| MF099238 | 8 (NS) | H1N2 | 10-08-14 | (A/swine/Chile/59/2014(H1N2)) |
| MF099249 | 7 (MP) | H1N2 | 10-08-14 | (A/swine/Chile/59/2014(H1N2)) |
| MF099272 | 5 (NP) | H1N2 | 10-08-14 | (A/swine/Chile/59/2014(H1N2)) |
| MF099315 | 1 (PB2) | H1N2 | 10-08-14 | (A/swine/Chile/59/2014(H1N2)) |
| MF099347 | 6 (NA) | H1N2 | 10-08-14 | (A/swine/Chile/59/2014(H1N2)) |
| MF099366 | 3 (PA) | H1N2 | 10-08-14 | (A/swine/Chile/59/2014(H1N2)) |
| MF098918 | 5 (NP) | H1N2 | 10-08-14 | (A/swine/Chile/60/2014(H1N2)) |
| MF098941 | 4 (HA) | H1N2 | 10-08-14 | (A/swine/Chile/60/2014(H1N2)) |
| MF099000 | 7 (MP) | H1N2 | 10-08-14 | (A/swine/Chile/60/2014(H1N2)) |
| MF099034 | 8 (NS) | H1N2 | 10-08-14 | (A/swine/Chile/60/2014(H1N2)) |
| MF099085 | 1 (PB2) | H1N2 | 10-08-14 | (A/swine/Chile/60/2014(H1N2)) |
| MF099115 | 2 (PB1) | H1N2 | 10-08-14 | (A/swine/Chile/60/2014(H1N2)) |
| MF099146 | 6 (NA) | H1N2 | 10-08-14 | (A/swine/Chile/60/2014(H1N2)) |
| MF099369 | 3 (PA) | H1N2 | 10-08-14 | (A/swine/Chile/60/2014(H1N2)) |
| MF098840 | 3 (PA) | H1N1 | 21-08-14 | (A/swine/Chile/65/2014(H1N1)) |
| MF099058 | 7 (MP) | H1N1 | 21-08-14 | (A/swine/Chile/65/2014(H1N1)) |
| MF099162 | 1 (PB2) | H1N1 | 21-08-14 | (A/swine/Chile/65/2014(H1N1)) |
| MF099191 | 4 (HA) | H1N1 | 21-08-14 | (A/swine/Chile/65/2014(H1N1)) |
| MF099214 | 6 (NA) | H1N1 | 21-08-14 | (A/swine/Chile/65/2014(H1N1)) |
| MF099268 | 2 (PB1) | H1N1 | 21-08-14 | (A/swine/Chile/65/2014(H1N1)) |
| MF099287 | 8 (NS) | H1N1 | 21-08-14 | (A/swine/Chile/65/2014(H1N1)) |
| MF099379 | 5 (NP) | H1N1 | 21-08-14 | (A/swine/Chile/65/2014(H1N1)) |
| MF098904 | 7 (MP) | H1N1 | 21-08-14 | (A/swine/Chile/66/2014(H1N1)) |
| MF099120 | 3 (PA) | H1N1 | 21-08-14 | (A/swine/Chile/66/2014(H1N1)) |
| MF099147 | 8 (NS) | H1N1 | 21-08-14 | (A/swine/Chile/66/2014(H1N1)) |
| MF099177 | 5 (NP) | H1N1 | 21-08-14 | (A/swine/Chile/66/2014(H1N1)) |
| MF099195 | 4 (HA) | H1N1 | 21-08-14 | (A/swine/Chile/66/2014(H1N1)) |
| MF099284 | 6 (NA) | H1N1 | 21-08-14 | (A/swine/Chile/66/2014(H1N1)) |
| MF099377 | 2 (PB1) | H1N1 | 21-08-14 | (A/swine/Chile/66/2014(H1N1)) |
| MF099383 | 1 (PB2) | H1N1 | 21-08-14 | (A/swine/Chile/66/2014(H1N1)) |
| KR870293 | 4 (HA) | H1N2 | 25-07-12 | (A/swine/Chile/9210581/2012(H1N2)) |
| KR870294 | 4 (HA) | H1N1 | 25-07-12 | (A/swine/Chile/9210582/2012(H1N1)) |
| KR870295 | 4 (HA) | H1N1 | 25-07-12 | (A/swine/Chile/9210585/2012(H1N1)) |
| KR870296 | 4 (HA) | H1N2 | 23-08-12 | (A/swine/Chile/9271613/2012(H1N2)) |
| KR870297 | 4 (HA) | H1N2 | 23-08-12 | (A/swine/Chile/9271617/2012(H1N2)) |
| KR870298 | 4 (HA) | H1 | 23-08-12 | (A/swine/Chile/9271625/2012(H1)) |
| KR870299 | 4 (HA) | H1N1 | 23-08-12 | (A/swine/Chile/9271633/2012(H1N1)) |
| KR870300 | 4 (HA) | H1N2 | 23-08-12 | (A/swine/Chile/9271642/2012(H1N2)) |
| KR870301 | 4 (HA) | H1 | 23-08-12 | (A/swine/Chile/9271644/2012(H1)) |
| KR870302 | 4 (HA) | H1N1 | 13-09-12 | (A/swine/Chile/9313891/2012(H1N1)) |
| KR870303 | 4 (HA) | H3N2 | 10-07-12 | (A/swine/Chile/9313892/2012(H3N2)) |
| KR870304 | 4 (HA) | H3N2 | 06-08-12 | (A/swine/Chile/9313893/2012(H3N2)) |
| KR870305 | 4 (HA) | H1N1 | 10-07-12 | (A/swine/Chile/9313894/2012(H1N1)) |
| KR870306 | 4 (HA) | H1 | 08-08-12 | (A/swine/Chile/9313895/2012(H1)) |
| KR870307 | 4 (HA) | H1 | 10-07-12 | (A/swine/Chile/9313896/2012(H1)) |
| KR870308 | 4 (HA) | H1N1 | 08-08-12 | (A/swine/Chile/9313897/2012(H1N1)) |
| KR870309 | 4 (HA) | H1N1 | 08-08-12 | (A/swine/Chile/9313898/2012(H1N1)) |
| KR870310 | 4 (HA) | H1N1 | 08-08-12 | (A/swine/Chile/9313899/2012(H1N1)) |
| MN857641 | 4 (HA) | H1N2 | 23-07-14 | (A/swine/Chile/VN1401-137/2014) |
| MN857642 | 4 (HA) | H1N2 | 23-07-14 | (A/swine/Chile/VN1401-144/2014) |
| MF099002 | 7 (MP) | H1N2 | 08-10-14 | (A/swine/Chile/VN1401-263/2014(H1N2)) |
| MF099114 | 3 (PA) | H1N2 | 08-10-14 | (A/swine/Chile/VN1401-263/2014(H1N2)) |
| MF099158 | 4 (HA) | H1N2 | 08-10-14 | (A/swine/Chile/VN1401-263/2014(H1N2)) |
| MF099166 | 1 (PB2) | H1N2 | 08-10-14 | (A/swine/Chile/VN1401-263/2014(H1N2)) |
| MF099182 | 6 (NA) | H1N2 | 08-10-14 | (A/swine/Chile/VN1401-263/2014(H1N2)) |
| MF099197 | 5 (NP) | H1N2 | 08-10-14 | (A/swine/Chile/VN1401-263/2014(H1N2)) |
| MF099229 | 2 (PB1) | H1N2 | 08-10-14 | (A/swine/Chile/VN1401-263/2014(H1N2)) |
| MF099350 | 8 (NS) | H1N2 | 08-10-14 | (A/swine/Chile/VN1401-263/2014(H1N2)) |
| MF098877 | 1 (PB2) | H1N2 | 08-10-14 | (A/swine/Chile/VN1401-271/2014(H1N2)) |
| MF098908 | 4 (HA) | H1N2 | 08-10-14 | (A/swine/Chile/VN1401-271/2014(H1N2)) |
| MF098958 | 6 (NA) | H1N2 | 08-10-14 | (A/swine/Chile/VN1401-271/2014(H1N2)) |
| MF098987 | 7 (MP) | H1N2 | 08-10-14 | (A/swine/Chile/VN1401-271/2014(H1N2)) |
| MF099140 | 3 (PA) | H1N2 | 08-10-14 | (A/swine/Chile/VN1401-271/2014(H1N2)) |
| MF099236 | 8 (NS) | H1N2 | 08-10-14 | (A/swine/Chile/VN1401-271/2014(H1N2)) |
| MF099289 | 5 (NP) | H1N2 | 08-10-14 | (A/swine/Chile/VN1401-271/2014(H1N2)) |
| MF099361 | 2 (PB1) | H1N2 | 08-10-14 | (A/swine/Chile/VN1401-271/2014(H1N2)) |
| MF098854 | 1 (PB2) | H1N2 | 08-10-14 | (A/swine/Chile/VN1401-274/2014(H1N2)) |
| MF098920 | 5 (NP) | H1N2 | 08-10-14 | (A/swine/Chile/VN1401-274/2014(H1N2)) |
| MF098970 | 8 (NS) | H1N2 | 08-10-14 | (A/swine/Chile/VN1401-274/2014(H1N2)) |
| MF099149 | 4 (HA) | H1N2 | 08-10-14 | (A/swine/Chile/VN1401-274/2014(H1N2)) |
| MF099190 | 2 (PB1) | H1N2 | 08-10-14 | (A/swine/Chile/VN1401-274/2014(H1N2)) |
| MF099217 | 3 (PA) | H1N2 | 08-10-14 | (A/swine/Chile/VN1401-274/2014(H1N2)) |
| MF099327 | 6 (NA) | H1N2 | 08-10-14 | (A/swine/Chile/VN1401-274/2014(H1N2)) |
| MF099374 | 7 (MP) | H1N2 | 08-10-14 | (A/swine/Chile/VN1401-274/2014(H1N2)) |
| MF098814 | 3 (PA) | H1N1 | 16-10-14 | (A/swine/Chile/VN1401-336/2014(H1N1)) |
| MF098843 | 6 (NA) | H1N1 | 16-10-14 | (A/swine/Chile/VN1401-336/2014(H1N1)) |
| MF098866 | 8 (NS) | H1N1 | 16-10-14 | (A/swine/Chile/VN1401-336/2014(H1N1)) |
| MF099207 | 5 (NP) | H1N1 | 16-10-14 | (A/swine/Chile/VN1401-336/2014(H1N1)) |
| MF099221 | 1 (PB2) | H1N1 | 16-10-14 | (A/swine/Chile/VN1401-336/2014(H1N1)) |
| MF099286 | 2 (PB1) | H1N1 | 16-10-14 | (A/swine/Chile/VN1401-336/2014(H1N1)) |
| MF099341 | 7 (MP) | H1N1 | 16-10-14 | (A/swine/Chile/VN1401-336/2014(H1N1)) |
| MF099368 | 4 (HA) | H1N1 | 16-10-14 | (A/swine/Chile/VN1401-336/2014(H1N1)) |
| MF098947 | 5 (NP) | H1N2 | 17-10-14 | (A/swine/Chile/VN1401-339/2014(H1N2)) |
| MF098974 | 1 (PB2) | H1N2 | 17-10-14 | (A/swine/Chile/VN1401-339/2014(H1N2)) |
| MF099066 | 3 (PA) | H1N2 | 17-10-14 | (A/swine/Chile/VN1401-339/2014(H1N2)) |
| MF099151 | 6 (NA) | H1N2 | 17-10-14 | (A/swine/Chile/VN1401-339/2014(H1N2)) |
| MF099311 | 2 (PB1) | H1N2 | 17-10-14 | (A/swine/Chile/VN1401-339/2014(H1N2)) |
| MF099322 | 8 (NS) | H1N2 | 17-10-14 | (A/swine/Chile/VN1401-339/2014(H1N2)) |
| MF099323 | 4 (HA) | H1N2 | 17-10-14 | (A/swine/Chile/VN1401-339/2014(H1N2)) |
| MF099405 | 7 (MP) | H1N2 | 17-10-14 | (A/swine/Chile/VN1401-339/2014(H1N2)) |
| MF098846 | 1 (PB2) | H1N2 | 17-10-14 | (A/swine/Chile/VN1401-343/2014(H1N2)) |
| MF098875 | 6 (NA) | H1N2 | 17-10-14 | (A/swine/Chile/VN1401-343/2014(H1N2)) |
| MF098881 | 7 (MP) | H1N2 | 17-10-14 | (A/swine/Chile/VN1401-343/2014(H1N2)) |
| MF098955 | 4 (HA) | H1N2 | 17-10-14 | (A/swine/Chile/VN1401-343/2014(H1N2)) |
| MF099119 | 5 (NP) | H1N2 | 17-10-14 | (A/swine/Chile/VN1401-343/2014(H1N2)) |
| MF099288 | 2 (PB1) | H1N2 | 17-10-14 | (A/swine/Chile/VN1401-343/2014(H1N2)) |
| MF099319 | 8 (NS) | H1N2 | 17-10-14 | (A/swine/Chile/VN1401-343/2014(H1N2)) |
| MF099345 | 3 (PA) | H1N2 | 17-10-14 | (A/swine/Chile/VN1401-343/2014(H1N2)) |
| MF098838 | 2 (PB1) | H1N2 | 17-10-14 | (A/swine/Chile/VN1401-367/2014(H1N2)) |
| MF098849 | 8 (NS) | H1N2 | 17-10-14 | (A/swine/Chile/VN1401-367/2014(H1N2)) |
| MF098954 | 1 (PB2) | H1N2 | 17-10-14 | (A/swine/Chile/VN1401-367/2014(H1N2)) |
| MF098959 | 5 (NP) | H1N2 | 17-10-14 | (A/swine/Chile/VN1401-367/2014(H1N2)) |
| MF099004 | 3 (PA) | H1N2 | 17-10-14 | (A/swine/Chile/VN1401-367/2014(H1N2)) |
| MF099098 | 7 (MP) | H1N2 | 17-10-14 | (A/swine/Chile/VN1401-367/2014(H1N2)) |
| MF099173 | 4 (HA) | H1N2 | 17-10-14 | (A/swine/Chile/VN1401-367/2014(H1N2)) |
| MF099293 | 6 (NA) | H1N2 | 17-10-14 | (A/swine/Chile/VN1401-367/2014(H1N2)) |
| MN857643 | 4 (HA) | H1N2 | 17-10-14 | (A/swine/Chile/VN1401-389/2014) |
| MF098932 | 7 (MP) | H1N2 | 25-02-14 | (A/swine/Chile/VN1401-4/2014(H1N2)) |
| MF099038 | 8 (NS) | H1N2 | 25-02-14 | (A/swine/Chile/VN1401-4/2014(H1N2)) |
| MF099067 | 3 (PA) | H1N2 | 25-02-14 | (A/swine/Chile/VN1401-4/2014(H1N2)) |
| MF099073 | 4 (HA) | H1N2 | 25-02-14 | (A/swine/Chile/VN1401-4/2014(H1N2)) |
| MF099133 | 5 (NP) | H1N2 | 25-02-14 | (A/swine/Chile/VN1401-4/2014(H1N2)) |
| MF099189 | 2 (PB1) | H1N2 | 25-02-14 | (A/swine/Chile/VN1401-4/2014(H1N2)) |
| MF099251 | 1 (PB2) | H1N2 | 25-02-14 | (A/swine/Chile/VN1401-4/2014(H1N2)) |
| MF099355 | 6 (NA) | H1N2 | 25-02-14 | (A/swine/Chile/VN1401-4/2014(H1N2)) |
| MF098902 | 4 (HA) | H1N2 | 24-04-14 | (A/swine/Chile/VN1401-41/2014(H1N2)) |
| MF098957 | 1 (PB2) | H1N2 | 24-04-14 | (A/swine/Chile/VN1401-41/2014(H1N2)) |
| MF098971 | 3 (PA) | H1N2 | 24-04-14 | (A/swine/Chile/VN1401-41/2014(H1N2)) |
| MF099060 | 5 (NP) | H1N2 | 24-04-14 | (A/swine/Chile/VN1401-41/2014(H1N2)) |
| MF099171 | 6 (NA) | H1N2 | 24-04-14 | (A/swine/Chile/VN1401-41/2014(H1N2)) |
| MF099240 | 6 (NA) | H1N2 | 24-04-14 | (A/swine/Chile/VN1401-41/2014(H1N2)) |
| MF099242 | 8 (NS) | H1N2 | 24-04-14 | (A/swine/Chile/VN1401-41/2014(H1N2)) |
| MF099303 | 7 (MP) | H1N2 | 24-04-14 | (A/swine/Chile/VN1401-41/2014(H1N2)) |
| MF099359 | 2 (PB1) | H1N2 | 24-04-14 | (A/swine/Chile/VN1401-41/2014(H1N2)) |
| MN857644 | 4 (HA) | H1N2 | 17-11-14 | (A/swine/Chile/VN1401-454/2014) |
| MN857645 | 4 (HA) | H1N2 | 17-11-14 | (A/swine/Chile/VN1401-482/2014) |
| MN857638 | 4 (HA) | H1N1 | 24-11-14 | (A/swine/Chile/VN1401-508/2014) |
| MF098823 | 5 (NP) | H1N1 | 24-11-14 | (A/swine/Chile/VN1401-510/2014(H1N1)) |
| MF099072 | 7 (MP) | H1N1 | 24-11-14 | (A/swine/Chile/VN1401-510/2014(H1N1)) |
| MF099324 | 4 (HA) | H1N1 | 24-11-14 | (A/swine/Chile/VN1401-510/2014(H1N1)) |
| MF099329 | 1 (PB2) | H1N1 | 24-11-14 | (A/swine/Chile/VN1401-510/2014(H1N1)) |
| MF099343 | 8 (NS) | H1N1 | 24-11-14 | (A/swine/Chile/VN1401-510/2014(H1N1)) |
| MF099357 | 2 (PB1) | H1N1 | 24-11-14 | (A/swine/Chile/VN1401-510/2014(H1N1)) |
| MF099363 | 6 (NA) | H1N1 | 24-11-14 | (A/swine/Chile/VN1401-510/2014(H1N1)) |
| MF099399 | 3 (PA) | H1N1 | 24-11-14 | (A/swine/Chile/VN1401-510/2014(H1N1)) |
| MN857639 | 4 (HA) | H1N1 | 24-11-14 | (A/swine/Chile/VN1401-525/2014) |
| MN857640 | 4 (HA) | H1N1 | 24-11-14 | (A/swine/Chile/VN1401-536/2014) |
| MF098839 | 1 (PB2) | H1N1 | 01-12-14 | (A/swine/Chile/VN1401-571/2014(H1N1)) |
| MF098896 | 7 (MP) | H1N1 | 01-12-14 | (A/swine/Chile/VN1401-571/2014(H1N1)) |
| MF098916 | 5 (NP) | H1N1 | 01-12-14 | (A/swine/Chile/VN1401-571/2014(H1N1)) |
| MF099090 | 3 (PA) | H1N1 | 01-12-14 | (A/swine/Chile/VN1401-571/2014(H1N1)) |
| MF099135 | 2 (PB1) | H1N1 | 01-12-14 | (A/swine/Chile/VN1401-571/2014(H1N1)) |
| MF099145 | 8 (NS) | H1N1 | 01-12-14 | (A/swine/Chile/VN1401-571/2014(H1N1)) |
| MF099203 | 6 (NA) | H1N1 | 01-12-14 | (A/swine/Chile/VN1401-571/2014(H1N1)) |
| MF099276 | 4 (HA) | H1N1 | 01-12-14 | (A/swine/Chile/VN1401-571/2014(H1N1)) |
| MF098827 | 4 (HA) | H1N1 | 01-12-14 | (A/swine/Chile/VN1401-587/2014(H1N1)) |
| MF098930 | 7 (MP) | H1N1 | 01-12-14 | (A/swine/Chile/VN1401-587/2014(H1N1)) |
| MF098993 | 2 (PB1) | H1N1 | 01-12-14 | (A/swine/Chile/VN1401-587/2014(H1N1)) |
| MF099015 | 1 (PB2) | H1N1 | 01-12-14 | (A/swine/Chile/VN1401-587/2014(H1N1)) |
| MF099082 | 8 (NS) | H1N1 | 01-12-14 | (A/swine/Chile/VN1401-587/2014(H1N1)) |
| MF099094 | 6 (NA) | H1N1 | 01-12-14 | (A/swine/Chile/VN1401-587/2014(H1N1)) |
| MF099156 | 4 (HA) | H1N1 | 01-12-14 | (A/swine/Chile/VN1401-587/2014(H1N1)) |
| MF099159 | 5 (NP) | H1N1 | 01-12-14 | (A/swine/Chile/VN1401-587/2014(H1N1)) |
| MF099208 | 6 (NA) | H1N1 | 01-12-14 | (A/swine/Chile/VN1401-587/2014(H1N1)) |
| MF099263 | 3 (PA) | H1N1 | 01-12-14 | (A/swine/Chile/VN1401-587/2014(H1N1)) |
| MF098869 | 4 (HA) | H1N1 | 05-06-14 | (A/swine/Chile/VN1401-80/2014(H1N1)) |
| MF098895 | 2 (PB1) | H1N1 | 05-06-14 | (A/swine/Chile/VN1401-80/2014(H1N1)) |
| MF099011 | 3 (PA) | H1N1 | 05-06-14 | (A/swine/Chile/VN1401-80/2014(H1N1)) |
| MF099113 | 7 (MP) | H1N1 | 05-06-14 | (A/swine/Chile/VN1401-80/2014(H1N1)) |
| MF099129 | 6 (NA) | H1N1 | 05-06-14 | (A/swine/Chile/VN1401-80/2014(H1N1)) |
| MF099219 | 5 (NP) | H1N1 | 05-06-14 | (A/swine/Chile/VN1401-80/2014(H1N1)) |
| MF099309 | 1 (PB2) | H1N1 | 05-06-14 | (A/swine/Chile/VN1401-80/2014(H1N1)) |
| MF099331 | 8 (NS) | H1N1 | 05-06-14 | (A/swine/Chile/VN1401-80/2014(H1N1)) |
| MF098821 | 6 (NA) | H1N1 | 05-06-14 | (A/swine/Chile/VN1401-82/2014(H1N1)) |
| MF098981 | 1 (PB2) | H1N1 | 05-06-14 | (A/swine/Chile/VN1401-82/2014(H1N1)) |
| MF098988 | 2 (PB1) | H1N1 | 05-06-14 | (A/swine/Chile/VN1401-82/2014(H1N1)) |
| MF099007 | 8 (NS) | H1N1 | 05-06-14 | (A/swine/Chile/VN1401-82/2014(H1N1)) |
| MF099008 | 5 (NP) | H1N1 | 05-06-14 | (A/swine/Chile/VN1401-82/2014(H1N1)) |
| MF099110 | 3 (PA) | H1N1 | 05-06-14 | (A/swine/Chile/VN1401-82/2014(H1N1)) |
| MF099161 | 4 (HA) | H1N1 | 05-06-14 | (A/swine/Chile/VN1401-82/2014(H1N1)) |
| MF099330 | 7 (MP) | H1N1 | 05-06-14 | (A/swine/Chile/VN1401-82/2014(H1N1)) |
| MF103721 | 1 (PB2) | H1 | 25-02-14 | (A/swine/Mallarauco/VN1401-03/2014(H1)) |
| MF103722 | 8 (NS) | H1 | 25-02-14 | (A/swine/Mallarauco/VN1401-03/2014(H1)) |
| MF103723 | 7 (MP) | H1 | 25-02-14 | (A/swine/Mallarauco/VN1401-03/2014(H1)) |
| MF103724 | 2 (PB1) | H1 | 25-02-14 | (A/swine/Mallarauco/VN1401-03/2014(H1)) |
| MF103725 | 3 (PA) | H1 | 25-02-14 | (A/swine/Mallarauco/VN1401-03/2014(H1)) |
| MF103726 | 4 (HA) | H1 | 25-02-14 | (A/swine/Mallarauco/VN1401-03/2014(H1)) |
| MF103727 | 5 (NP) | H1 | 25-02-14 | (A/swine/Mallarauco/VN1401-03/2014(H1)) |
| MN054095 | 1 (PB2) | H1N1 | 2013 | (A/swine/Nuble/VN1401-3755/2013) |
| MN054096 | 6 (NA) | H1N1 | 2013 | (A/swine/Nuble/VN1401-3755/2013) |
| MN054097 | 3 (PA) | H1N1 | 2013 | (A/swine/Nuble/VN1401-3755/2013) |
| MN054098 | 5 (NP) | H1N1 | 2013 | (A/swine/Nuble/VN1401-3755/2013) |
| MN054099 | 7 (MP) | H1N1 | 2013 | (A/swine/Nuble/VN1401-3755/2013) |
| MN054101 | 4 (HA) | H1N1 | 2013 | (A/swine/Nuble/VN1401-3755/2013) |
| MN054417 | 2 (PB1) | H1N1 | 2013 | (A/swine/Nuble/VN1401-3755/2013) |
| MN054100 | 8 (NS) | H1N1 | 2013 | (A/swine/Nuble/VN1401-3755/2013) |
| MN054138 | 6 (NA) | H1N1 | 2013 | (A/swine/Nuble/VN1401-3756/2013) |
| MN054525 | 2 (PB1) | H1N1 | 2013 | (A/swine/Nuble/VN1401-3756/2013) |
| MN054139 | 1 (PB2) | H1N1 | 2013 | (A/swine/Nuble/VN1401-3756/2013) |
| MN054137 | 5 (NP) | H1N1 | 2013 | (A/swine/Nuble/VN1401-3756/2013) |
| MN054136 | 4 (HA) | H1N1 | 2013 | (A/swine/Nuble/VN1401-3756/2013) |
| MN054135 | 8 (NS) | H1N1 | 2013 | (A/swine/Nuble/VN1401-3756/2013) |
| MN054134 | 3 (PA) | H1N1 | 2013 | (A/swine/Nuble/VN1401-3756/2013) |
| MN054133 | 7 (MP) | H1N1 | 2013 | (A/swine/Nuble/VN1401-3756/2013) |
| MN054292 | 7 (MP) | H1N1 | 2013 | (A/swine/Nuble/VN1401-3761/2013) |
| MN054293 | 8 (NS) | H1N1 | 2013 | (A/swine/Nuble/VN1401-3761/2013) |
| MN054294 | 1 (PB2) | H1N1 | 2013 | (A/swine/Nuble/VN1401-3761/2013) |
| MN054295 | 4 (HA) | H1N1 | 2013 | (A/swine/Nuble/VN1401-3761/2013) |
| MN054296 | 6 (NA) | H1N1 | 2013 | (A/swine/Nuble/VN1401-3761/2013) |
| MN054297 | 5 (NP) | H1N1 | 2013 | (A/swine/Nuble/VN1401-3761/2013) |
| MN054298 | 3 (PA) | H1N1 | 2013 | (A/swine/Nuble/VN1401-3761/2013) |
| MN055117 | 2 (PB1) | H1N1 | 2013 | (A/swine/Nuble/VN1401-3761/2013) |
| MN054185 | 3 (PA) | H1N1 | 2013 | (A/swine/Nuble/VN1401-3762/2013) |
| MN054448 | 2 (PB1) | H1N1 | 2013 | (A/swine/Nuble/VN1401-3762/2013) |
| MN054181 | 1 (PB2) | H1N1 | 2013 | (A/swine/Nuble/VN1401-3762/2013) |
| MN054182 | 8 (NS) | H1N1 | 2013 | (A/swine/Nuble/VN1401-3762/2013) |
| MN054183 | 7 (MP) | H1N1 | 2013 | (A/swine/Nuble/VN1401-3762/2013) |
| MN054184 | 6 (NA) | H1N1 | 2013 | (A/swine/Nuble/VN1401-3762/2013) |
| MN054186 | 5 (NP) | H1N1 | 2013 | (A/swine/Nuble/VN1401-3762/2013) |
| MN054187 | 4 (HA) | H1N1 | 2013 | (A/swine/Nuble/VN1401-3762/2013) |
| MN055144 | 6 (NA) | H1N1 | 2013 | (A/swine/Nuble/VN1401-3763/2013) |
| MN055145 | 5 (NP) | H1N1 | 2013 | (A/swine/Nuble/VN1401-3763/2013) |
| MN055146 | 7 (MP) | H1N1 | 2013 | (A/swine/Nuble/VN1401-3763/2013) |
| MN055147 | 1 (PB2) | H1N1 | 2013 | (A/swine/Nuble/VN1401-3763/2013) |
| MN055148 | 3 (PA) | H1N1 | 2013 | (A/swine/Nuble/VN1401-3763/2013) |
| MN055149 | 4 (HA) | H1N1 | 2013 | (A/swine/Nuble/VN1401-3763/2013) |
| MN055150 | 8 (NS) | H1N1 | 2013 | (A/swine/Nuble/VN1401-3763/2013) |
| MN055221 | 2 (PB1) | H1N1 | 2013 | (A/swine/Nuble/VN1401-3763/2013) |
| MN055076 | 2 (PB1) | H1N1 | 2013 | (A/swine/Nuble/VN1401-3764/2013) |
| MN054256 | 8 (NS) | H1N1 | 2013 | (A/swine/Nuble/VN1401-3764/2013) |
| MN054257 | 4 (HA) | H1N1 | 2013 | (A/swine/Nuble/VN1401-3764/2013) |
| MN054258 | 6 (NA) | H1N1 | 2013 | (A/swine/Nuble/VN1401-3764/2013) |
| MN054259 | 7 (MP) | H1N1 | 2013 | (A/swine/Nuble/VN1401-3764/2013) |
| MN054260 | 3 (PA) | H1N1 | 2013 | (A/swine/Nuble/VN1401-3764/2013) |
| MN054261 | 1 (PB2) | H1N1 | 2013 | (A/swine/Nuble/VN1401-3764/2013) |
| MN054262 | 5 (NP) | H1N1 | 2013 | (A/swine/Nuble/VN1401-3764/2013) |
| MN055069 | 4 (HA) | H1N1 | 2013 | (A/swine/Nuble/VN1401-3972/2013) |
| MN055070 | 7 (MP) | H1N1 | 2013 | (A/swine/Nuble/VN1401-3972/2013) |
| MN055071 | 6 (NA) | H1N1 | 2013 | (A/swine/Nuble/VN1401-3972/2013) |
| MN055072 | 3 (PA) | H1N1 | 2013 | (A/swine/Nuble/VN1401-3972/2013) |
| MN055073 | 1 (PB2) | H1N1 | 2013 | (A/swine/Nuble/VN1401-3972/2013) |
| MN055074 | 8 (NS) | H1N1 | 2013 | (A/swine/Nuble/VN1401-3972/2013) |
| MN055075 | 5 (NP) | H1N1 | 2013 | (A/swine/Nuble/VN1401-3972/2013) |
| MN055395 | 2 (PB1) | H1N1 | 2013 | (A/swine/Nuble/VN1401-3972/2013) |
| MN055135 | 2 (PB1) | H1N1 | 2013 | (A/swine/O'Higgins/VN1401-3768/2013) |
| MN055388 | 7 (MP) | H1N1 | 2013 | (A/swine/O'Higgins/VN1401-3768/2013) |
| MN055389 | 5 (NP) | H1N1 | 2013 | (A/swine/O'Higgins/VN1401-3768/2013) |
| MN055390 | 3 (PA) | H1N1 | 2013 | (A/swine/O'Higgins/VN1401-3768/2013) |
| MN055391 | 1 (PB2) | H1N1 | 2013 | (A/swine/O'Higgins/VN1401-3768/2013) |
| MN055392 | 4 (HA) | H1N1 | 2013 | (A/swine/O'Higgins/VN1401-3768/2013) |
| MN055393 | 8 (NS) | H1N1 | 2013 | (A/swine/O'Higgins/VN1401-3768/2013) |
| MN055394 | 6 (NA) | H1N1 | 2013 | (A/swine/O'Higgins/VN1401-3768/2013) |
| MN054506 | 7 (MP) | H1N1 | 2013 | (A/swine/O'Higgins/VN1401-3770/2013) |
| MN054507 | 4 (HA) | H1N1 | 2013 | (A/swine/O'Higgins/VN1401-3770/2013) |
| MN054508 | 8 (NS) | H1N1 | 2013 | (A/swine/O'Higgins/VN1401-3770/2013) |
| MN054509 | 6 (NA) | H1N1 | 2013 | (A/swine/O'Higgins/VN1401-3770/2013) |
| MN054510 | 5 (NP) | H1N1 | 2013 | (A/swine/O'Higgins/VN1401-3770/2013) |
| MF098842 | 6 (NA) | H1N1 | 17-10-14 | (A/swine/O'Higgins/VN1401-380/2014(H1N1)) |
| MF098925 | 1 (PB2) | H1N1 | 17-10-14 | (A/swine/O'Higgins/VN1401-380/2014(H1N1)) |
| MF098966 | 5 (NP) | H1N1 | 17-10-14 | (A/swine/O'Higgins/VN1401-380/2014(H1N1)) |
| MF099019 | 4 (HA) | H1N1 | 17-10-14 | (A/swine/O'Higgins/VN1401-380/2014(H1N1)) |
| MF099107 | 8 (NS) | H1N1 | 17-10-14 | (A/swine/O'Higgins/VN1401-380/2014(H1N1)) |
| MF099143 | 2 (PB1) | H1N1 | 17-10-14 | (A/swine/O'Higgins/VN1401-380/2014(H1N1)) |
| MF099239 | 3 (PA) | H1N1 | 17-10-14 | (A/swine/O'Higgins/VN1401-380/2014(H1N1)) |
| MF099279 | 7 (MP) | H1N1 | 17-10-14 | (A/swine/O'Higgins/VN1401-380/2014(H1N1)) |
| MF098909 | 2 (PB1) | H1N2 | 23-07-14 | (A/swine/Rancagua/VN1401-143/2014(H1N2)) |
| MF098911 | 1 (PB2) | H1N2 | 23-07-14 | (A/swine/Rancagua/VN1401-143/2014(H1N2)) |
| MF098928 | 5 (NP) | H1N2 | 23-07-14 | (A/swine/Rancagua/VN1401-143/2014(H1N2)) |
| MF099062 | 4 (HA) | H1N2 | 23-07-14 | (A/swine/Rancagua/VN1401-143/2014(H1N2)) |
| MF099200 | 7 (MP) | H1N2 | 23-07-14 | (A/swine/Rancagua/VN1401-143/2014(H1N2)) |
| MF099328 | 8 (NS) | H1N2 | 23-07-14 | (A/swine/Rancagua/VN1401-143/2014(H1N2)) |
| MF099340 | 3 (PA) | H1N2 | 23-07-14 | (A/swine/Rancagua/VN1401-143/2014(H1N2)) |
| MF099372 | 6 (NA) | H1N2 | 23-07-14 | (A/swine/Rancagua/VN1401-143/2014(H1N2)) |
| MF098874 | 4 (HA) | H1N1 | 19-02-14 | (A/swine/Rancagua/VN1401-19/2014(H1N1)) |
| MF098951 | 1 (PB2) | H1N1 | 19-02-14 | (A/swine/Rancagua/VN1401-19/2014(H1N1)) |
| MF099032 | 3 (PA) | H1N1 | 19-02-14 | (A/swine/Rancagua/VN1401-19/2014(H1N1)) |
| MF099087 | 5 (NP) | H1N1 | 19-02-14 | (A/swine/Rancagua/VN1401-19/2014(H1N1)) |
| MF099095 | 6 (NA) | H1N1 | 19-02-14 | (A/swine/Rancagua/VN1401-19/2014(H1N1)) |
| MF099132 | 2 (PB1) | H1N1 | 19-02-14 | (A/swine/Rancagua/VN1401-19/2014(H1N1)) |
| MF099266 | 8 (NS) | H1N1 | 19-02-14 | (A/swine/Rancagua/VN1401-19/2014(H1N1)) |
| MF099313 | 7 (MP) | H1N1 | 19-02-14 | (A/swine/Rancagua/VN1401-19/2014(H1N1)) |
| MF098820 | 3 (PA) | H1N1 | 19-02-14 | (A/swine/Rancagua/VN1401-23/2014(H1N1)) |
| MF098851 | 7 (MP) | H1N1 | 19-02-14 | (A/swine/Rancagua/VN1401-23/2014(H1N1)) |
| MF099057 | 5 (NP) | H1N1 | 19-02-14 | (A/swine/Rancagua/VN1401-23/2014(H1N1)) |
| MF099091 | 8 (NS) | H1N1 | 19-02-14 | (A/swine/Rancagua/VN1401-23/2014(H1N1)) |
| MF099164 | 1 (PB2) | H1N1 | 19-02-14 | (A/swine/Rancagua/VN1401-23/2014(H1N1)) |
| MF099185 | 6 (NA) | H1N1 | 19-02-14 | (A/swine/Rancagua/VN1401-23/2014(H1N1)) |
| MF099373 | 2 (PB1) | H1N1 | 19-02-14 | (A/swine/Rancagua/VN1401-23/2014(H1N1)) |
| MF099392 | 4 (HA) | H1N1 | 19-02-14 | (A/swine/Rancagua/VN1401-23/2014(H1N1)) |
| MF098939 | 8 (NS) | H1N1 | 05-06-14 | (A/swine/Rengo/VN1401-83/2014(H1N1)) |
| MF098960 | 4 (HA) | H1N1 | 05-06-14 | (A/swine/Rengo/VN1401-83/2014(H1N1)) |
| MF099020 | 7 (MP) | H1N1 | 05-06-14 | (A/swine/Rengo/VN1401-83/2014(H1N1)) |
| MF099022 | 2 (PB1) | H1N1 | 05-06-14 | (A/swine/Rengo/VN1401-83/2014(H1N1)) |
| MF099079 | 1 (PB2) | H1N1 | 05-06-14 | (A/swine/Rengo/VN1401-83/2014(H1N1)) |
| MF099138 | 3 (PA) | H1N1 | 05-06-14 | (A/swine/Rengo/VN1401-83/2014(H1N1)) |
| MF099261 | 6 (NA) | H1N1 | 05-06-14 | (A/swine/Rengo/VN1401-83/2014(H1N1)) |
| MF099384 | 5 (NP) | H1N1 | 05-06-14 | (A/swine/Rengo/VN1401-83/2014(H1N1)) |
| MF098830 | 4 (HA) | H1N1 | 05-06-14 | (A/swine/Rengo/VN1401-94/2014(H1N1)) |
| MF099074 | 8 (NS) | H1N1 | 05-06-14 | (A/swine/Rengo/VN1401-94/2014(H1N1)) |
| MF099169 | 3 (PA) | H1N1 | 05-06-14 | (A/swine/Rengo/VN1401-94/2014(H1N1)) |
| MF099172 | 6 (NA) | H1N1 | 05-06-14 | (A/swine/Rengo/VN1401-94/2014(H1N1)) |
| MF099198 | 7 (MP) | H1N1 | 05-06-14 | (A/swine/Rengo/VN1401-94/2014(H1N1)) |
| MF099235 | 1 (PB2) | H1N1 | 05-06-14 | (A/swine/Rengo/VN1401-94/2014(H1N1)) |
| MF099394 | 2 (PB1) | H1N1 | 05-06-14 | (A/swine/Rengo/VN1401-94/2014(H1N1)) |
| MF099396 | 5 (NP) | H1N1 | 05-06-14 | (A/swine/Rengo/VN1401-94/2014(H1N1)) |
| MF098856 | 6 (NA) | H1N1 | 05-06-14 | (A/swine/Rengo/VN1401-95/2014(H1N1)) |
| MF098887 | 3 (PA) | H1N1 | 05-06-14 | (A/swine/Rengo/VN1401-95/2014(H1N1)) |
| MF099128 | 7 (MP) | H1N1 | 05-06-14 | (A/swine/Rengo/VN1401-95/2014(H1N1)) |
| MF099199 | 5 (NP) | H1N1 | 05-06-14 | (A/swine/Rengo/VN1401-95/2014(H1N1)) |
| MF099212 | 1 (PB2) | H1N1 | 05-06-14 | (A/swine/Rengo/VN1401-95/2014(H1N1)) |
| MF099253 | 2 (PB1) | H1N1 | 05-06-14 | (A/swine/Rengo/VN1401-95/2014(H1N1)) |
| MF099360 | 4 (HA) | H1N1 | 05-06-14 | (A/swine/Rengo/VN1401-95/2014(H1N1)) |
| MF099367 | 8 (NS) | H1N1 | 05-06-14 | (A/swine/Rengo/VN1401-95/2014(H1N1)) |
| ADO12143 | HA | H1N1 | 2009/05/23 | (A/Chile/28/2009(H1N1)) |
| ADO12144 | M1 | H1N1 | 2009/05/23 | (A/Chile/28/2009(H1N1)) |
| ADO12145 | M2 | H1N1 | 2009/05/23 | (A/Chile/28/2009(H1N1)) |
| ADO12146 | NA | H1N1 | 2009/05/23 | (A/Chile/28/2009(H1N1)) |
| ADO12147 | NP | H1N1 | 2009/05/23 | (A/Chile/28/2009(H1N1)) |
| ADO12148 | NS1 | H1N1 | 2009/05/23 | (A/Chile/28/2009(H1N1)) |
| ADO12149 | NS2 | H1N1 | 2009/05/23 | (A/Chile/28/2009(H1N1)) |
| ADO12150 | PA | H1N1 | 2009/05/23 | (A/Chile/28/2009(H1N1)) |
| ADO12151 | PB1 | H1N1 | 2009/05/23 | (A/Chile/28/2009(H1N1)) |
| ADO12152 | PB2 | H1N1 | 2009/05/23 | (A/Chile/28/2009(H1N1)) |
| ADO12153 | HA | H1N1 | 2009/05/25 | (A/Chile/31/2009(H1N1)) |
| ADO12154 | M1 | H1N1 | 2009/05/25 | (A/Chile/31/2009(H1N1)) |
| ADO12155 | M2 | H1N1 | 2009/05/25 | (A/Chile/31/2009(H1N1)) |
| ADO12156 | NA | H1N1 | 2009/05/25 | (A/Chile/31/2009(H1N1)) |
| ADO12157 | NP | H1N1 | 2009/05/25 | (A/Chile/31/2009(H1N1)) |
| ADO12158 | NS1 | H1N1 | 2009/05/25 | (A/Chile/31/2009(H1N1)) |
| ADO12159 | NS2 | H1N1 | 2009/05/25 | (A/Chile/31/2009(H1N1)) |
| ADO12160 | PA | H1N1 | 2009/05/25 | (A/Chile/31/2009(H1N1)) |
| ADO12161 | PB1 | H1N1 | 2009/05/25 | (A/Chile/31/2009(H1N1)) |
| ADO12162 | PB2 | H1N1 | 2009/05/25 | (A/Chile/31/2009(H1N1)) |
| ADO12163 | HA | H1N1 | 2009/05/25 | (A/Chile/32/2009(H1N1)) |
| ADO12164 | M1 | H1N1 | 2009/05/25 | (A/Chile/32/2009(H1N1)) |
| ADO12165 | M2 | H1N1 | 2009/05/25 | (A/Chile/32/2009(H1N1)) |
| ADO12166 | NA | H1N1 | 2009/05/25 | (A/Chile/32/2009(H1N1)) |
| ADO12167 | NP | H1N1 | 2009/05/25 | (A/Chile/32/2009(H1N1)) |
| ADO12168 | NS1 | H1N1 | 2009/05/25 | (A/Chile/32/2009(H1N1)) |
| ADO12169 | NS2 | H1N1 | 2009/05/25 | (A/Chile/32/2009(H1N1)) |
| ADO12170 | PA | H1N1 | 2009/05/25 | (A/Chile/32/2009(H1N1)) |
| ADO12171 | PB1 | H1N1 | 2009/05/25 | (A/Chile/32/2009(H1N1)) |
| ADO12172 | PB2 | H1N1 | 2009/05/25 | (A/Chile/32/2009(H1N1)) |
| ADO12173 | HA | H1N1 | 2009/05/25 | (A/Chile/36/2009(H1N1)) |
| ADO12174 | M1 | H1N1 | 2009/05/25 | (A/Chile/36/2009(H1N1)) |
| ADO12175 | M2 | H1N1 | 2009/05/25 | (A/Chile/36/2009(H1N1)) |
| ADO12176 | NA | H1N1 | 2009/05/25 | (A/Chile/36/2009(H1N1)) |
| ADO12177 | NP | H1N1 | 2009/05/25 | (A/Chile/36/2009(H1N1)) |
| ADO12178 | NS1 | H1N1 | 2009/05/25 | (A/Chile/36/2009(H1N1)) |
| ADO12179 | NS2 | H1N1 | 2009/05/25 | (A/Chile/36/2009(H1N1)) |
| ADO12180 | PA | H1N1 | 2009/05/25 | (A/Chile/36/2009(H1N1)) |
| ADO12181 | PB1 | H1N1 | 2009/05/25 | (A/Chile/36/2009(H1N1)) |
| ADO12182 | PB2 | H1N1 | 2009/05/25 | (A/Chile/36/2009(H1N1)) |
| ADO12183 | HA | H1N1 | 2009/05/25 | (A/Chile/40/2009(H1N1)) |
| ADO12184 | M1 | H1N1 | 2009/05/25 | (A/Chile/40/2009(H1N1)) |
| ADO12185 | M2 | H1N1 | 2009/05/25 | (A/Chile/40/2009(H1N1)) |
| ADO12186 | NA | H1N1 | 2009/05/25 | (A/Chile/40/2009(H1N1)) |
| ADO12187 | NP | H1N1 | 2009/05/25 | (A/Chile/40/2009(H1N1)) |
| ADO12188 | NS1 | H1N1 | 2009/05/25 | (A/Chile/40/2009(H1N1)) |
| ADO12189 | NS2 | H1N1 | 2009/05/25 | (A/Chile/40/2009(H1N1)) |
| ADO12190 | PA | H1N1 | 2009/05/25 | (A/Chile/40/2009(H1N1)) |
| ADO12191 | PB1 | H1N1 | 2009/05/25 | (A/Chile/40/2009(H1N1)) |
| ADO12192 | PB2 | H1N1 | 2009/05/25 | (A/Chile/40/2009(H1N1)) |
| ADO12193 | HA | H1N1 | 2009/05/25 | (A/Chile/42/2009(H1N1)) |
| ADO12194 | M1 | H1N1 | 2009/05/25 | (A/Chile/42/2009(H1N1)) |
| ADO12195 | M2 | H1N1 | 2009/05/25 | (A/Chile/42/2009(H1N1)) |
| ADO12196 | NA | H1N1 | 2009/05/25 | (A/Chile/42/2009(H1N1)) |
| ADO12197 | NP | H1N1 | 2009/05/25 | (A/Chile/42/2009(H1N1)) |
| ADO12198 | NS1 | H1N1 | 2009/05/25 | (A/Chile/42/2009(H1N1)) |
| ADO12199 | NS2 | H1N1 | 2009/05/25 | (A/Chile/42/2009(H1N1)) |
| ADO12200 | PA | H1N1 | 2009/05/25 | (A/Chile/42/2009(H1N1)) |
| ADO12201 | PB1 | H1N1 | 2009/05/25 | (A/Chile/42/2009(H1N1)) |
| ADO12202 | PB2 | H1N1 | 2009/05/25 | (A/Chile/42/2009(H1N1)) |
| ADO12203 | HA | H1N1 | 2009/05/26 | (A/Chile/56/2009(H1N1)) |
| ADO12204 | M1 | H1N1 | 2009/05/26 | (A/Chile/56/2009(H1N1)) |
| ADO12205 | M2 | H1N1 | 2009/05/26 | (A/Chile/56/2009(H1N1)) |
| ADO12206 | NA | H1N1 | 2009/05/26 | (A/Chile/56/2009(H1N1)) |
| ADO12207 | NP | H1N1 | 2009/05/26 | (A/Chile/56/2009(H1N1)) |
| ADO12208 | NS1 | H1N1 | 2009/05/26 | (A/Chile/56/2009(H1N1)) |
| ADO12209 | NS2 | H1N1 | 2009/05/26 | (A/Chile/56/2009(H1N1)) |
| ADO12210 | PA | H1N1 | 2009/05/26 | (A/Chile/56/2009(H1N1)) |
| ADO12211 | PB1 | H1N1 | 2009/05/26 | (A/Chile/56/2009(H1N1)) |
| ADO12212 | PB2 | H1N1 | 2009/05/26 | (A/Chile/56/2009(H1N1)) |
| ADO12213 | HA | H1N1 | 2009/05/27 | (A/Chile/88/2009(H1N1)) |
| ADO12214 | M1 | H1N1 | 2009/05/27 | (A/Chile/88/2009(H1N1)) |
| ADO12215 | M2 | H1N1 | 2009/05/27 | (A/Chile/88/2009(H1N1)) |
| ADO12216 | NA | H1N1 | 2009/05/27 | (A/Chile/88/2009(H1N1)) |
| ADO12217 | NP | H1N1 | 2009/05/27 | (A/Chile/88/2009(H1N1)) |
| ADO12218 | NS1 | H1N1 | 2009/05/27 | (A/Chile/88/2009(H1N1)) |
| ADO12219 | NS2 | H1N1 | 2009/05/27 | (A/Chile/88/2009(H1N1)) |
| ADO12220 | PA | H1N1 | 2009/05/27 | (A/Chile/88/2009(H1N1)) |
| ADO12221 | PB1 | H1N1 | 2009/05/27 | (A/Chile/88/2009(H1N1)) |
| ADO12222 | PB2 | H1N1 | 2009/05/27 | (A/Chile/88/2009(H1N1)) |
| ADO12223 | HA | H1N1 | 2009/05/27 | (A/Chile/95/2009(H1N1)) |
| ADO12224 | M1 | H1N1 | 2009/05/27 | (A/Chile/95/2009(H1N1)) |
| ADO12225 | M2 | H1N1 | 2009/05/27 | (A/Chile/95/2009(H1N1)) |
| ADO12226 | NA | H1N1 | 2009/05/27 | (A/Chile/95/2009(H1N1)) |
| ADO12227 | NP | H1N1 | 2009/05/27 | (A/Chile/95/2009(H1N1)) |
| ADO12228 | NS1 | H1N1 | 2009/05/27 | (A/Chile/95/2009(H1N1)) |
| ADO12229 | NS2 | H1N1 | 2009/05/27 | (A/Chile/95/2009(H1N1)) |
| ADO12230 | PA | H1N1 | 2009/05/27 | (A/Chile/95/2009(H1N1)) |
| ADO12231 | PB1 | H1N1 | 2009/05/27 | (A/Chile/95/2009(H1N1)) |
| ADO12232 | PB2 | H1N1 | 2009/05/27 | (A/Chile/95/2009(H1N1)) |
| ADO12233 | HA | H1N1 | 2009/05/28 | (A/Chile/158/2009(H1N1)) |
| ADO12234 | M1 | H1N1 | 2009/05/28 | (A/Chile/158/2009(H1N1)) |
| ADO12235 | M2 | H1N1 | 2009/05/28 | (A/Chile/158/2009(H1N1)) |
| ADO12236 | NA | H1N1 | 2009/05/28 | (A/Chile/158/2009(H1N1)) |
| ADO12237 | NP | H1N1 | 2009/05/28 | (A/Chile/158/2009(H1N1)) |
| ADO12238 | NS1 | H1N1 | 2009/05/28 | (A/Chile/158/2009(H1N1)) |
| ADO12239 | NS2 | H1N1 | 2009/05/28 | (A/Chile/158/2009(H1N1)) |
| ADO12240 | PA | H1N1 | 2009/05/28 | (A/Chile/158/2009(H1N1)) |
| ADO12241 | PB1 | H1N1 | 2009/05/28 | (A/Chile/158/2009(H1N1)) |
| ADO12242 | PB2 | H1N1 | 2009/05/28 | (A/Chile/158/2009(H1N1)) |
| ADO12243 | HA | H1N1 | 2009/05/29 | (A/Chile/180/2009(H1N1)) |
| ADO12244 | M1 | H1N1 | 2009/05/29 | (A/Chile/180/2009(H1N1)) |
| ADO12245 | M2 | H1N1 | 2009/05/29 | (A/Chile/180/2009(H1N1)) |
| ADO12246 | NA | H1N1 | 2009/05/29 | (A/Chile/180/2009(H1N1)) |
| ADO12247 | NP | H1N1 | 2009/05/29 | (A/Chile/180/2009(H1N1)) |
| ADO12248 | NS1 | H1N1 | 2009/05/29 | (A/Chile/180/2009(H1N1)) |
| ADO12249 | NS2 | H1N1 | 2009/05/29 | (A/Chile/180/2009(H1N1)) |
| ADO12250 | PA | H1N1 | 2009/05/29 | (A/Chile/180/2009(H1N1)) |
| ADO12251 | PB1 | H1N1 | 2009/05/29 | (A/Chile/180/2009(H1N1)) |
| ADO12252 | PB2 | H1N1 | 2009/05/29 | (A/Chile/180/2009(H1N1)) |
| ADO12253 | HA | H1N1 | 2009/06/13 | (A/Chile/1586/2009(H1N1)) |
| ADO12254 | M1 | H1N1 | 2009/06/13 | (A/Chile/1586/2009(H1N1)) |
| ADO12255 | M2 | H1N1 | 2009/06/13 | (A/Chile/1586/2009(H1N1)) |
| ADO12256 | NA | H1N1 | 2009/06/13 | (A/Chile/1586/2009(H1N1)) |
| ADO12257 | NP | H1N1 | 2009/06/13 | (A/Chile/1586/2009(H1N1)) |
| ADO12258 | NS1 | H1N1 | 2009/06/13 | (A/Chile/1586/2009(H1N1)) |
| ADO12259 | NS2 | H1N1 | 2009/06/13 | (A/Chile/1586/2009(H1N1)) |
| ADO12260 | PA | H1N1 | 2009/06/13 | (A/Chile/1586/2009(H1N1)) |
| ADO12261 | PB1 | H1N1 | 2009/06/13 | (A/Chile/1586/2009(H1N1)) |
| ADO12262 | PB2 | H1N1 | 2009/06/13 | (A/Chile/1586/2009(H1N1)) |
| ADO12263 | HA | H1N1 | 2009/06/15 | (A/Chile/1598/2009(H1N1)) |
| ADO12264 | M1 | H1N1 | 2009/06/15 | (A/Chile/1598/2009(H1N1)) |
| ADO12265 | M2 | H1N1 | 2009/06/15 | (A/Chile/1598/2009(H1N1)) |
| ADO12266 | NA | H1N1 | 2009/06/15 | (A/Chile/1598/2009(H1N1)) |
| ADO12267 | NP | H1N1 | 2009/06/15 | (A/Chile/1598/2009(H1N1)) |
| ADO12268 | NS1 | H1N1 | 2009/06/15 | (A/Chile/1598/2009(H1N1)) |
| ADO12269 | NS2 | H1N1 | 2009/06/15 | (A/Chile/1598/2009(H1N1)) |
| ADO12270 | PA | H1N1 | 2009/06/15 | (A/Chile/1598/2009(H1N1)) |
| ADO12271 | PB1 | H1N1 | 2009/06/15 | (A/Chile/1598/2009(H1N1)) |
| ADO12272 | PB2 | H1N1 | 2009/06/15 | (A/Chile/1598/2009(H1N1)) |
| ADO12273 | HA | H1N1 | 2009/06/15 | (A/Chile/1599/2009(H1N1)) |
| ADO12274 | M1 | H1N1 | 2009/06/15 | (A/Chile/1599/2009(H1N1)) |
| ADO12275 | M2 | H1N1 | 2009/06/15 | (A/Chile/1599/2009(H1N1)) |
| ADO12276 | NA | H1N1 | 2009/06/15 | (A/Chile/1599/2009(H1N1)) |
| ADO12277 | NP | H1N1 | 2009/06/15 | (A/Chile/1599/2009(H1N1)) |
| ADO12278 | NS1 | H1N1 | 2009/06/15 | (A/Chile/1599/2009(H1N1)) |
| ADO12279 | NS2 | H1N1 | 2009/06/15 | (A/Chile/1599/2009(H1N1)) |
| ADO12280 | PA | H1N1 | 2009/06/15 | (A/Chile/1599/2009(H1N1)) |
| ADO12281 | PB1 | H1N1 | 2009/06/15 | (A/Chile/1599/2009(H1N1)) |
| ADO12282 | PB2 | H1N1 | 2009/06/15 | (A/Chile/1599/2009(H1N1)) |
| ADO12283 | HA | H1N1 | 2009/06/15 | (A/Chile/1600/2009(H1N1)) |
| ADO12284 | M1 | H1N1 | 2009/06/15 | (A/Chile/1600/2009(H1N1)) |
| ADO12285 | M2 | H1N1 | 2009/06/15 | (A/Chile/1600/2009(H1N1)) |
| ADO12286 | NA | H1N1 | 2009/06/15 | (A/Chile/1600/2009(H1N1)) |
| ADO12287 | NP | H1N1 | 2009/06/15 | (A/Chile/1600/2009(H1N1)) |
| ADO12288 | NS1 | H1N1 | 2009/06/15 | (A/Chile/1600/2009(H1N1)) |
| ADO12289 | NS2 | H1N1 | 2009/06/15 | (A/Chile/1600/2009(H1N1)) |
| ADO12290 | PA | H1N1 | 2009/06/15 | (A/Chile/1600/2009(H1N1)) |
| ADO12291 | PB1 | H1N1 | 2009/06/15 | (A/Chile/1600/2009(H1N1)) |
| ADO12292 | PB2 | H1N1 | 2009/06/15 | (A/Chile/1600/2009(H1N1)) |
| ADO12293 | HA | H1N1 | 2009/06/15 | (A/Chile/1603/2009(H1N1)) |
| ADO12294 | M1 | H1N1 | 2009/06/15 | (A/Chile/1603/2009(H1N1)) |
| ADO12295 | M2 | H1N1 | 2009/06/15 | (A/Chile/1603/2009(H1N1)) |
| ADO12296 | NA | H1N1 | 2009/06/15 | (A/Chile/1603/2009(H1N1)) |
| ADO12297 | NP | H1N1 | 2009/06/15 | (A/Chile/1603/2009(H1N1)) |
| ADO12298 | NS1 | H1N1 | 2009/06/15 | (A/Chile/1603/2009(H1N1)) |
| ADO12299 | NS2 | H1N1 | 2009/06/15 | (A/Chile/1603/2009(H1N1)) |
| ADO12300 | PA | H1N1 | 2009/06/15 | (A/Chile/1603/2009(H1N1)) |
| ADO12301 | PB1 | H1N1 | 2009/06/15 | (A/Chile/1603/2009(H1N1)) |
| ADO12302 | PB2 | H1N1 | 2009/06/15 | (A/Chile/1603/2009(H1N1)) |
| ADO12303 | HA | H1N1 | 2009/06/15 | (A/Chile/1624/2009(H1N1)) |
| ADO12304 | M1 | H1N1 | 2009/06/15 | (A/Chile/1624/2009(H1N1)) |
| ADO12305 | M2 | H1N1 | 2009/06/15 | (A/Chile/1624/2009(H1N1)) |
| ADO12306 | NA | H1N1 | 2009/06/15 | (A/Chile/1624/2009(H1N1)) |
| ADO12307 | NP | H1N1 | 2009/06/15 | (A/Chile/1624/2009(H1N1)) |
| ADO12308 | NS1 | H1N1 | 2009/06/15 | (A/Chile/1624/2009(H1N1)) |
| ADO12309 | NS2 | H1N1 | 2009/06/15 | (A/Chile/1624/2009(H1N1)) |
| ADO12310 | PA | H1N1 | 2009/06/15 | (A/Chile/1624/2009(H1N1)) |
| ADO12311 | PB1 | H1N1 | 2009/06/15 | (A/Chile/1624/2009(H1N1)) |
| ADO12312 | PB2 | H1N1 | 2009/06/15 | (A/Chile/1624/2009(H1N1)) |
| ADO12313 | HA | H1N1 | 2009/06/18 | (A/Chile/2009/2009(H1N1)) |
| ADO12314 | M1 | H1N1 | 2009/06/18 | (A/Chile/2009/2009(H1N1)) |
| ADO12315 | M2 | H1N1 | 2009/06/18 | (A/Chile/2009/2009(H1N1)) |
| ADO12316 | NA | H1N1 | 2009/06/18 | (A/Chile/2009/2009(H1N1)) |
| ADO12317 | NP | H1N1 | 2009/06/18 | (A/Chile/2009/2009(H1N1)) |
| ADO12318 | NS1 | H1N1 | 2009/06/18 | (A/Chile/2009/2009(H1N1)) |
| ADO12319 | NS2 | H1N1 | 2009/06/18 | (A/Chile/2009/2009(H1N1)) |
| ADO12320 | PA | H1N1 | 2009/06/18 | (A/Chile/2009/2009(H1N1)) |
| ADO12321 | PB1 | H1N1 | 2009/06/18 | (A/Chile/2009/2009(H1N1)) |
| ADO12322 | PB2 | H1N1 | 2009/06/18 | (A/Chile/2009/2009(H1N1)) |
| ADO12323 | HA | H1N1 | 2009/06/19 | (A/Chile/2239/2009(H1N1)) |
| ADO12324 | M1 | H1N1 | 2009/06/19 | (A/Chile/2239/2009(H1N1)) |
| ADO12325 | M2 | H1N1 | 2009/06/19 | (A/Chile/2239/2009(H1N1)) |
| ADO12326 | NA | H1N1 | 2009/06/19 | (A/Chile/2239/2009(H1N1)) |
| ADO12327 | NP | H1N1 | 2009/06/19 | (A/Chile/2239/2009(H1N1)) |
| ADO12328 | NS1 | H1N1 | 2009/06/19 | (A/Chile/2239/2009(H1N1)) |
| ADO12329 | NS2 | H1N1 | 2009/06/19 | (A/Chile/2239/2009(H1N1)) |
| ADO12330 | PA | H1N1 | 2009/06/19 | (A/Chile/2239/2009(H1N1)) |
| ADO12331 | PB1 | H1N1 | 2009/06/19 | (A/Chile/2239/2009(H1N1)) |
| ADO12332 | PB2 | H1N1 | 2009/06/19 | (A/Chile/2239/2009(H1N1)) |
| ADO12333 | HA | H1N1 | 2009/06/20 | (A/Chile/2851/2009(H1N1)) |
| ADO12334 | M1 | H1N1 | 2009/06/20 | (A/Chile/2851/2009(H1N1)) |
| ADO12335 | M2 | H1N1 | 2009/06/20 | (A/Chile/2851/2009(H1N1)) |
| ADO12336 | NA | H1N1 | 2009/06/20 | (A/Chile/2851/2009(H1N1)) |
| ADO12337 | NP | H1N1 | 2009/06/20 | (A/Chile/2851/2009(H1N1)) |
| ADO12338 | NS1 | H1N1 | 2009/06/20 | (A/Chile/2851/2009(H1N1)) |
| ADO12339 | NS2 | H1N1 | 2009/06/20 | (A/Chile/2851/2009(H1N1)) |
| ADO12340 | PA | H1N1 | 2009/06/20 | (A/Chile/2851/2009(H1N1)) |
| ADO12341 | PB1 | H1N1 | 2009/06/20 | (A/Chile/2851/2009(H1N1)) |
| ADO12342 | PB2 | H1N1 | 2009/06/20 | (A/Chile/2851/2009(H1N1)) |
| ADO12343 | HA | H1N1 | 2009/06/22 | (A/Chile/2362/2009(H1N1)) |
| ADO12344 | M1 | H1N1 | 2009/06/22 | (A/Chile/2362/2009(H1N1)) |
| ADO12345 | M2 | H1N1 | 2009/06/22 | (A/Chile/2362/2009(H1N1)) |
| ADO12346 | NA | H1N1 | 2009/06/22 | (A/Chile/2362/2009(H1N1)) |
| ADO12347 | NP | H1N1 | 2009/06/22 | (A/Chile/2362/2009(H1N1)) |
| ADO12348 | NS1 | H1N1 | 2009/06/22 | (A/Chile/2362/2009(H1N1)) |
| ADO12349 | NS2 | H1N1 | 2009/06/22 | (A/Chile/2362/2009(H1N1)) |
| ADO12350 | PA | H1N1 | 2009/06/22 | (A/Chile/2362/2009(H1N1)) |
| ADO12351 | PB1 | H1N1 | 2009/06/22 | (A/Chile/2362/2009(H1N1)) |
| ADO12352 | PB2 | H1N1 | 2009/06/22 | (A/Chile/2362/2009(H1N1)) |
| ADO12353 | HA | H1N1 | 2009/06/25 | (A/Chile/2909/2009(H1N1)) |
| ADO12354 | M1 | H1N1 | 2009/06/25 | (A/Chile/2909/2009(H1N1)) |
| ADO12355 | M2 | H1N1 | 2009/06/25 | (A/Chile/2909/2009(H1N1)) |
| ADO12356 | NA | H1N1 | 2009/06/25 | (A/Chile/2909/2009(H1N1)) |
| ADO12357 | NP | H1N1 | 2009/06/25 | (A/Chile/2909/2009(H1N1)) |
| ADO12358 | NS1 | H1N1 | 2009/06/25 | (A/Chile/2909/2009(H1N1)) |
| ADO12359 | NS2 | H1N1 | 2009/06/25 | (A/Chile/2909/2009(H1N1)) |
| ADO12360 | PA | H1N1 | 2009/06/25 | (A/Chile/2909/2009(H1N1)) |
| ADO12361 | PB1 | H1N1 | 2009/06/25 | (A/Chile/2909/2009(H1N1)) |
| ADO12362 | PB2 | H1N1 | 2009/06/25 | (A/Chile/2909/2009(H1N1)) |
| ADO12363 | HA | H1N1 | 2009/06/25 | (A/Chile/2911/2009(H1N1)) |
| ADO12364 | M1 | H1N1 | 2009/06/25 | (A/Chile/2911/2009(H1N1)) |
| ADO12365 | M2 | H1N1 | 2009/06/25 | (A/Chile/2911/2009(H1N1)) |
| ADO12366 | NA | H1N1 | 2009/06/25 | (A/Chile/2911/2009(H1N1)) |
| ADO12367 | NP | H1N1 | 2009/06/25 | (A/Chile/2911/2009(H1N1)) |
| ADO12368 | NS1 | H1N1 | 2009/06/25 | (A/Chile/2911/2009(H1N1)) |
| ADO12369 | NS2 | H1N1 | 2009/06/25 | (A/Chile/2911/2009(H1N1)) |
| ADO12370 | PA | H1N1 | 2009/06/25 | (A/Chile/2911/2009(H1N1)) |
| ADO12371 | PB1 | H1N1 | 2009/06/25 | (A/Chile/2911/2009(H1N1)) |
| ADO12372 | PB2 | H1N1 | 2009/06/25 | (A/Chile/2911/2009(H1N1)) |
| ADO12373 | HA | H1N1 | 2009/06/26 | (A/Chile/2994/2009(H1N1)) |
| ADO12374 | M1 | H1N1 | 2009/06/26 | (A/Chile/2994/2009(H1N1)) |
| ADO12375 | M2 | H1N1 | 2009/06/26 | (A/Chile/2994/2009(H1N1)) |
| ADO12376 | NA | H1N1 | 2009/06/26 | (A/Chile/2994/2009(H1N1)) |
| ADO12377 | NP | H1N1 | 2009/06/26 | (A/Chile/2994/2009(H1N1)) |
| ADO12378 | NS1 | H1N1 | 2009/06/26 | (A/Chile/2994/2009(H1N1)) |
| ADO12379 | NS2 | H1N1 | 2009/06/26 | (A/Chile/2994/2009(H1N1)) |
| ADO12380 | PA | H1N1 | 2009/06/26 | (A/Chile/2994/2009(H1N1)) |
| ADO12381 | PB1 | H1N1 | 2009/06/26 | (A/Chile/2994/2009(H1N1)) |
| ADO12382 | PB2 | H1N1 | 2009/06/26 | (A/Chile/2994/2009(H1N1)) |
| ADO12383 | HA | H1N1 | 2009/06/26 | (A/Chile/3019/2009(H1N1)) |
| ADO12384 | M1 | H1N1 | 2009/06/26 | (A/Chile/3019/2009(H1N1)) |
| ADO12385 | M2 | H1N1 | 2009/06/26 | (A/Chile/3019/2009(H1N1)) |
| ADO12386 | NA | H1N1 | 2009/06/26 | (A/Chile/3019/2009(H1N1)) |
| ADO12387 | NP | H1N1 | 2009/06/26 | (A/Chile/3019/2009(H1N1)) |
| ADO12388 | NS1 | H1N1 | 2009/06/26 | (A/Chile/3019/2009(H1N1)) |
| ADO12389 | NS2 | H1N1 | 2009/06/26 | (A/Chile/3019/2009(H1N1)) |
| ADO12390 | PA | H1N1 | 2009/06/26 | (A/Chile/3019/2009(H1N1)) |
| ADO12391 | PB1 | H1N1 | 2009/06/26 | (A/Chile/3019/2009(H1N1)) |
| ADO12392 | PB2 | H1N1 | 2009/06/26 | (A/Chile/3019/2009(H1N1)) |
| ADO12393 | HA | H1N1 | 2009/06/26 | (A/Chile/3056/2009(H1N1)) |
| ADO12394 | M1 | H1N1 | 2009/06/26 | (A/Chile/3056/2009(H1N1)) |
| ADO12395 | M2 | H1N1 | 2009/06/26 | (A/Chile/3056/2009(H1N1)) |
| ADO12396 | NA | H1N1 | 2009/06/26 | (A/Chile/3056/2009(H1N1)) |
| ADO12397 | NP | H1N1 | 2009/06/26 | (A/Chile/3056/2009(H1N1)) |
| ADO12398 | NS1 | H1N1 | 2009/06/26 | (A/Chile/3056/2009(H1N1)) |
| ADO12399 | NS2 | H1N1 | 2009/06/26 | (A/Chile/3056/2009(H1N1)) |
| ADO12400 | PA | H1N1 | 2009/06/26 | (A/Chile/3056/2009(H1N1)) |
| ADO12401 | PB1 | H1N1 | 2009/06/26 | (A/Chile/3056/2009(H1N1)) |
| ADO12402 | PB2 | H1N1 | 2009/06/26 | (A/Chile/3056/2009(H1N1)) |
| ADO12403 | HA | H1N1 | 2009/06/26 | (A/Chile/3123/2009(H1N1)) |
| ADO12404 | M1 | H1N1 | 2009/06/26 | (A/Chile/3123/2009(H1N1)) |
| ADO12405 | M2 | H1N1 | 2009/06/26 | (A/Chile/3123/2009(H1N1)) |
| ADO12406 | NA | H1N1 | 2009/06/26 | (A/Chile/3123/2009(H1N1)) |
| ADO12407 | NP | H1N1 | 2009/06/26 | (A/Chile/3123/2009(H1N1)) |
| ADO12408 | NS1 | H1N1 | 2009/06/26 | (A/Chile/3123/2009(H1N1)) |
| ADO12409 | NS2 | H1N1 | 2009/06/26 | (A/Chile/3123/2009(H1N1)) |
| ADO12410 | PA | H1N1 | 2009/06/26 | (A/Chile/3123/2009(H1N1)) |
| ADO12411 | PB1 | H1N1 | 2009/06/26 | (A/Chile/3123/2009(H1N1)) |
| ADO12412 | PB2 | H1N1 | 2009/06/26 | (A/Chile/3123/2009(H1N1)) |
| ADO12413 | HA | H1N1 | 2009/06/30 | (A/Chile/3220/2009(H1N1)) |
| ADO12414 | M1 | H1N1 | 2009/06/30 | (A/Chile/3220/2009(H1N1)) |
| ADO12415 | M2 | H1N1 | 2009/06/30 | (A/Chile/3220/2009(H1N1)) |
| ADO12416 | NA | H1N1 | 2009/06/30 | (A/Chile/3220/2009(H1N1)) |
| ADO12417 | NP | H1N1 | 2009/06/30 | (A/Chile/3220/2009(H1N1)) |
| ADO12418 | NS1 | H1N1 | 2009/06/30 | (A/Chile/3220/2009(H1N1)) |
| ADO12419 | NS2 | H1N1 | 2009/06/30 | (A/Chile/3220/2009(H1N1)) |
| ADO12420 | PA | H1N1 | 2009/06/30 | (A/Chile/3220/2009(H1N1)) |
| ADO12421 | PB1 | H1N1 | 2009/06/30 | (A/Chile/3220/2009(H1N1)) |
| ADO12422 | PB2 | H1N1 | 2009/06/30 | (A/Chile/3220/2009(H1N1)) |
| ADO12423 | HA | H1N1 | 2009/06/30 | (A/Chile/3242/2009(H1N1)) |
| ADO12424 | M1 | H1N1 | 2009/06/30 | (A/Chile/3242/2009(H1N1)) |
| ADO12425 | M2 | H1N1 | 2009/06/30 | (A/Chile/3242/2009(H1N1)) |
| ADO12426 | NA | H1N1 | 2009/06/30 | (A/Chile/3242/2009(H1N1)) |
| ADO12427 | NP | H1N1 | 2009/06/30 | (A/Chile/3242/2009(H1N1)) |
| ADO12428 | NS1 | H1N1 | 2009/06/30 | (A/Chile/3242/2009(H1N1)) |
| ADO12429 | NS2 | H1N1 | 2009/06/30 | (A/Chile/3242/2009(H1N1)) |
| ADO12430 | PA | H1N1 | 2009/06/30 | (A/Chile/3242/2009(H1N1)) |
| ADO12431 | PB1 | H1N1 | 2009/06/30 | (A/Chile/3242/2009(H1N1)) |
| ADO12432 | PB2 | H1N1 | 2009/06/30 | (A/Chile/3242/2009(H1N1)) |
| ADO12433 | HA | H1N1 | 2009/06/30 | (A/Chile/3244/2009(H1N1)) |
| ADO12434 | M1 | H1N1 | 2009/06/30 | (A/Chile/3244/2009(H1N1)) |
| ADO12435 | M2 | H1N1 | 2009/06/30 | (A/Chile/3244/2009(H1N1)) |
| ADO12436 | NA | H1N1 | 2009/06/30 | (A/Chile/3244/2009(H1N1)) |
| ADO12437 | NP | H1N1 | 2009/06/30 | (A/Chile/3244/2009(H1N1)) |
| ADO12438 | NS1 | H1N1 | 2009/06/30 | (A/Chile/3244/2009(H1N1)) |
| ADO12439 | NS2 | H1N1 | 2009/06/30 | (A/Chile/3244/2009(H1N1)) |
| ADO12440 | PA | H1N1 | 2009/06/30 | (A/Chile/3244/2009(H1N1)) |
| ADO12441 | PB1 | H1N1 | 2009/06/30 | (A/Chile/3244/2009(H1N1)) |
| ADO12442 | PB2 | H1N1 | 2009/06/30 | (A/Chile/3244/2009(H1N1)) |
| ADO12443 | HA | H1N1 | 2009/06/30 | (A/Chile/3295/2009(H1N1)) |
| ADO12444 | M1 | H1N1 | 2009/06/30 | (A/Chile/3295/2009(H1N1)) |
| ADO12445 | M2 | H1N1 | 2009/06/30 | (A/Chile/3295/2009(H1N1)) |
| ADO12446 | NA | H1N1 | 2009/06/30 | (A/Chile/3295/2009(H1N1)) |
| ADO12447 | NP | H1N1 | 2009/06/30 | (A/Chile/3295/2009(H1N1)) |
| ADO12448 | NS1 | H1N1 | 2009/06/30 | (A/Chile/3295/2009(H1N1)) |
| ADO12449 | NS2 | H1N1 | 2009/06/30 | (A/Chile/3295/2009(H1N1)) |
| ADO12450 | PA | H1N1 | 2009/06/30 | (A/Chile/3295/2009(H1N1)) |
| ADO12451 | PB1 | H1N1 | 2009/06/30 | (A/Chile/3295/2009(H1N1)) |
| ADO12452 | PB2 | H1N1 | 2009/06/30 | (A/Chile/3295/2009(H1N1)) |
| ADO12453 | HA | H1N1 | 2009/07/01 | (A/Chile/3349/2009(H1N1)) |
| ADO12454 | M1 | H1N1 | 2009/07/01 | (A/Chile/3349/2009(H1N1)) |
| ADO12455 | M2 | H1N1 | 2009/07/01 | (A/Chile/3349/2009(H1N1)) |
| ADO12456 | NA | H1N1 | 2009/07/01 | (A/Chile/3349/2009(H1N1)) |
| ADO12457 | NP | H1N1 | 2009/07/01 | (A/Chile/3349/2009(H1N1)) |
| ADO12458 | NS1 | H1N1 | 2009/07/01 | (A/Chile/3349/2009(H1N1)) |
| ADO12459 | NS2 | H1N1 | 2009/07/01 | (A/Chile/3349/2009(H1N1)) |
| ADO12460 | PA | H1N1 | 2009/07/01 | (A/Chile/3349/2009(H1N1)) |
| ADO12461 | PB1 | H1N1 | 2009/07/01 | (A/Chile/3349/2009(H1N1)) |
| ADO12462 | PB2 | H1N1 | 2009/07/01 | (A/Chile/3349/2009(H1N1)) |
| ADO12463 | HA | H1N1 | 2009/07/01 | (A/Chile/3361/2009(H1N1)) |
| ADO12464 | M1 | H1N1 | 2009/07/01 | (A/Chile/3361/2009(H1N1)) |
| ADO12465 | M2 | H1N1 | 2009/07/01 | (A/Chile/3361/2009(H1N1)) |
| ADO12466 | NA | H1N1 | 2009/07/01 | (A/Chile/3361/2009(H1N1)) |
| ADO12467 | NP | H1N1 | 2009/07/01 | (A/Chile/3361/2009(H1N1)) |
| ADO12468 | NS1 | H1N1 | 2009/07/01 | (A/Chile/3361/2009(H1N1)) |
| ADO12469 | NS2 | H1N1 | 2009/07/01 | (A/Chile/3361/2009(H1N1)) |
| ADO12470 | PA | H1N1 | 2009/07/01 | (A/Chile/3361/2009(H1N1)) |
| ADO12471 | PB1 | H1N1 | 2009/07/01 | (A/Chile/3361/2009(H1N1)) |
| ADO12472 | PB2 | H1N1 | 2009/07/01 | (A/Chile/3361/2009(H1N1)) |
| ADO12473 | HA | H1N1 | 2009/07/01 | (A/Chile/3369/2009(H1N1)) |
| ADO12474 | M1 | H1N1 | 2009/07/01 | (A/Chile/3369/2009(H1N1)) |
| ADO12475 | M2 | H1N1 | 2009/07/01 | (A/Chile/3369/2009(H1N1)) |
| ADO12476 | NA | H1N1 | 2009/07/01 | (A/Chile/3369/2009(H1N1)) |
| ADO12477 | NP | H1N1 | 2009/07/01 | (A/Chile/3369/2009(H1N1)) |
| ADO12478 | NS1 | H1N1 | 2009/07/01 | (A/Chile/3369/2009(H1N1)) |
| ADO12479 | NS2 | H1N1 | 2009/07/01 | (A/Chile/3369/2009(H1N1)) |
| ADO12480 | PA | H1N1 | 2009/07/01 | (A/Chile/3369/2009(H1N1)) |
| ADO12481 | PB1 | H1N1 | 2009/07/01 | (A/Chile/3369/2009(H1N1)) |
| ADO12482 | PB2 | H1N1 | 2009/07/01 | (A/Chile/3369/2009(H1N1)) |
| ADO12483 | HA | H1N1 | 2009/07/01 | (A/Chile/3375/2009(H1N1)) |
| ADO12484 | M1 | H1N1 | 2009/07/01 | (A/Chile/3375/2009(H1N1)) |
| ADO12485 | M2 | H1N1 | 2009/07/01 | (A/Chile/3375/2009(H1N1)) |
| ADO12486 | NA | H1N1 | 2009/07/01 | (A/Chile/3375/2009(H1N1)) |
| ADO12487 | NP | H1N1 | 2009/07/01 | (A/Chile/3375/2009(H1N1)) |
| ADO12488 | NS1 | H1N1 | 2009/07/01 | (A/Chile/3375/2009(H1N1)) |
| ADO12489 | NS2 | H1N1 | 2009/07/01 | (A/Chile/3375/2009(H1N1)) |
| ADO12490 | PA | H1N1 | 2009/07/01 | (A/Chile/3375/2009(H1N1)) |
| ADO12491 | PB1 | H1N1 | 2009/07/01 | (A/Chile/3375/2009(H1N1)) |
| ADO12492 | PB2 | H1N1 | 2009/07/01 | (A/Chile/3375/2009(H1N1)) |
| ADO12493 | HA | H1N1 | 2009/07/02 | (A/Chile/3467/2009(H1N1)) |
| ADO12494 | M1 | H1N1 | 2009/07/02 | (A/Chile/3467/2009(H1N1)) |
| ADO12495 | M2 | H1N1 | 2009/07/02 | (A/Chile/3467/2009(H1N1)) |
| ADO12496 | NA | H1N1 | 2009/07/02 | (A/Chile/3467/2009(H1N1)) |
| ADO12497 | NP | H1N1 | 2009/07/02 | (A/Chile/3467/2009(H1N1)) |
| ADO12498 | NS1 | H1N1 | 2009/07/02 | (A/Chile/3467/2009(H1N1)) |
| ADO12499 | NS2 | H1N1 | 2009/07/02 | (A/Chile/3467/2009(H1N1)) |
| ADO12500 | PA | H1N1 | 2009/07/02 | (A/Chile/3467/2009(H1N1)) |
| ADO12501 | PB1 | H1N1 | 2009/07/02 | (A/Chile/3467/2009(H1N1)) |
| ADO12502 | PB2 | H1N1 | 2009/07/02 | (A/Chile/3467/2009(H1N1)) |
| ADO12503 | HA | H1N1 | 2009/07/02 | (A/Chile/3553/2009(H1N1)) |
| ADO12504 | M1 | H1N1 | 2009/07/02 | (A/Chile/3553/2009(H1N1)) |
| ADO12505 | M2 | H1N1 | 2009/07/02 | (A/Chile/3553/2009(H1N1)) |
| ADO12506 | NA | H1N1 | 2009/07/02 | (A/Chile/3553/2009(H1N1)) |
| ADO12507 | NP | H1N1 | 2009/07/02 | (A/Chile/3553/2009(H1N1)) |
| ADO12508 | NS1 | H1N1 | 2009/07/02 | (A/Chile/3553/2009(H1N1)) |
| ADO12509 | NS2 | H1N1 | 2009/07/02 | (A/Chile/3553/2009(H1N1)) |
| ADO12510 | PA | H1N1 | 2009/07/02 | (A/Chile/3553/2009(H1N1)) |
| ADO12511 | PB1 | H1N1 | 2009/07/02 | (A/Chile/3553/2009(H1N1)) |
| ADO12512 | PB2 | H1N1 | 2009/07/02 | (A/Chile/3553/2009(H1N1)) |
| ADO12513 | HA | H1N1 | 2009/07/03 | (A/Chile/3586/2009(H1N1)) |
| ADO12514 | M1 | H1N1 | 2009/07/03 | (A/Chile/3586/2009(H1N1)) |
| ADO12515 | M2 | H1N1 | 2009/07/03 | (A/Chile/3586/2009(H1N1)) |
| ADO12516 | NA | H1N1 | 2009/07/03 | (A/Chile/3586/2009(H1N1)) |
| ADO12517 | NP | H1N1 | 2009/07/03 | (A/Chile/3586/2009(H1N1)) |
| ADO12518 | NS1 | H1N1 | 2009/07/03 | (A/Chile/3586/2009(H1N1)) |
| ADO12519 | NS2 | H1N1 | 2009/07/03 | (A/Chile/3586/2009(H1N1)) |
| ADO12520 | PA | H1N1 | 2009/07/03 | (A/Chile/3586/2009(H1N1)) |
| ADO12521 | PB1 | H1N1 | 2009/07/03 | (A/Chile/3586/2009(H1N1)) |
| ADO12522 | PB2 | H1N1 | 2009/07/03 | (A/Chile/3586/2009(H1N1)) |
| ADO12523 | HA | H1N1 | 2009/07/06 | (A/Chile/3760/2009(H1N1)) |
| ADO12524 | M1 | H1N1 | 2009/07/06 | (A/Chile/3760/2009(H1N1)) |
| ADO12525 | M2 | H1N1 | 2009/07/06 | (A/Chile/3760/2009(H1N1)) |
| ADO12526 | NA | H1N1 | 2009/07/06 | (A/Chile/3760/2009(H1N1)) |
| ADO12527 | NP | H1N1 | 2009/07/06 | (A/Chile/3760/2009(H1N1)) |
| ADO12528 | NS1 | H1N1 | 2009/07/06 | (A/Chile/3760/2009(H1N1)) |
| ADO12529 | NS2 | H1N1 | 2009/07/06 | (A/Chile/3760/2009(H1N1)) |
| ADO12530 | PA | H1N1 | 2009/07/06 | (A/Chile/3760/2009(H1N1)) |
| ADO12531 | PB1 | H1N1 | 2009/07/06 | (A/Chile/3760/2009(H1N1)) |
| ADO12532 | PB2 | H1N1 | 2009/07/06 | (A/Chile/3760/2009(H1N1)) |
| ADO12533 | HA | H1N1 | 2009/07/06 | (A/Chile/3766/2009(H1N1)) |
| ADO12534 | M1 | H1N1 | 2009/07/06 | (A/Chile/3766/2009(H1N1)) |
| ADO12535 | M2 | H1N1 | 2009/07/06 | (A/Chile/3766/2009(H1N1)) |
| ADO12536 | NA | H1N1 | 2009/07/06 | (A/Chile/3766/2009(H1N1)) |
| ADO12537 | NP | H1N1 | 2009/07/06 | (A/Chile/3766/2009(H1N1)) |
| ADO12538 | NS1 | H1N1 | 2009/07/06 | (A/Chile/3766/2009(H1N1)) |
| ADO12539 | NS2 | H1N1 | 2009/07/06 | (A/Chile/3766/2009(H1N1)) |
| ADO12540 | PA | H1N1 | 2009/07/06 | (A/Chile/3766/2009(H1N1)) |
| ADO12541 | PB1 | H1N1 | 2009/07/06 | (A/Chile/3766/2009(H1N1)) |
| ADO12542 | PB2 | H1N1 | 2009/07/06 | (A/Chile/3766/2009(H1N1)) |
| ADO12543 | HA | H1N1 | 2009/07/06 | (A/Chile/3819/2009(H1N1)) |
| ADO12544 | M1 | H1N1 | 2009/07/06 | (A/Chile/3819/2009(H1N1)) |
| ADO12545 | M2 | H1N1 | 2009/07/06 | (A/Chile/3819/2009(H1N1)) |
| ADO12546 | NA | H1N1 | 2009/07/06 | (A/Chile/3819/2009(H1N1)) |
| ADO12547 | NP | H1N1 | 2009/07/06 | (A/Chile/3819/2009(H1N1)) |
| ADO12548 | NS1 | H1N1 | 2009/07/06 | (A/Chile/3819/2009(H1N1)) |
| ADO12549 | NS2 | H1N1 | 2009/07/06 | (A/Chile/3819/2009(H1N1)) |
| ADO12550 | PA | H1N1 | 2009/07/06 | (A/Chile/3819/2009(H1N1)) |
| ADO12551 | PB1 | H1N1 | 2009/07/06 | (A/Chile/3819/2009(H1N1)) |
| ADO12552 | PB2 | H1N1 | 2009/07/06 | (A/Chile/3819/2009(H1N1)) |
| ADO12553 | HA | H1N1 | 2009/07/07 | (A/Chile/3905/2009(H1N1)) |
| ADO12554 | M1 | H1N1 | 2009/07/07 | (A/Chile/3905/2009(H1N1)) |
| ADO12555 | M2 | H1N1 | 2009/07/07 | (A/Chile/3905/2009(H1N1)) |
| ADO12556 | NA | H1N1 | 2009/07/07 | (A/Chile/3905/2009(H1N1)) |
| ADO12557 | NP | H1N1 | 2009/07/07 | (A/Chile/3905/2009(H1N1)) |
| ADO12558 | NS1 | H1N1 | 2009/07/07 | (A/Chile/3905/2009(H1N1)) |
| ADO12559 | NS2 | H1N1 | 2009/07/07 | (A/Chile/3905/2009(H1N1)) |
| ADO12560 | PA | H1N1 | 2009/07/07 | (A/Chile/3905/2009(H1N1)) |
| ADO12561 | PB1 | H1N1 | 2009/07/07 | (A/Chile/3905/2009(H1N1)) |
| ADO12562 | PB2 | H1N1 | 2009/07/07 | (A/Chile/3905/2009(H1N1)) |
| ADO12563 | HA | H1N1 | 2009/07/07 | (A/Chile/3935/2009(H1N1)) |
| ADO12564 | M1 | H1N1 | 2009/07/07 | (A/Chile/3935/2009(H1N1)) |
| ADO12565 | M2 | H1N1 | 2009/07/07 | (A/Chile/3935/2009(H1N1)) |
| ADO12566 | NA | H1N1 | 2009/07/07 | (A/Chile/3935/2009(H1N1)) |
| ADO12567 | NP | H1N1 | 2009/07/07 | (A/Chile/3935/2009(H1N1)) |
| ADO12568 | NS1 | H1N1 | 2009/07/07 | (A/Chile/3935/2009(H1N1)) |
| ADO12569 | NS2 | H1N1 | 2009/07/07 | (A/Chile/3935/2009(H1N1)) |
| ADO12570 | PA | H1N1 | 2009/07/07 | (A/Chile/3935/2009(H1N1)) |
| ADO12571 | PB1 | H1N1 | 2009/07/07 | (A/Chile/3935/2009(H1N1)) |
| ADO12572 | PB2 | H1N1 | 2009/07/07 | (A/Chile/3935/2009(H1N1)) |
| ADO12573 | HA | H1N1 | 2009/07/09 | (A/Chile/4064/2009(H1N1)) |
| ADO12574 | M1 | H1N1 | 2009/07/09 | (A/Chile/4064/2009(H1N1)) |
| ADO12575 | M2 | H1N1 | 2009/07/09 | (A/Chile/4064/2009(H1N1)) |
| ADO12576 | NA | H1N1 | 2009/07/09 | (A/Chile/4064/2009(H1N1)) |
| ADO12577 | NP | H1N1 | 2009/07/09 | (A/Chile/4064/2009(H1N1)) |
| ADO12578 | NS1 | H1N1 | 2009/07/09 | (A/Chile/4064/2009(H1N1)) |
| ADO12579 | NS2 | H1N1 | 2009/07/09 | (A/Chile/4064/2009(H1N1)) |
| ADO12580 | PA | H1N1 | 2009/07/09 | (A/Chile/4064/2009(H1N1)) |
| ADO12581 | PB1 | H1N1 | 2009/07/09 | (A/Chile/4064/2009(H1N1)) |
| ADO12582 | PB2 | H1N1 | 2009/07/09 | (A/Chile/4064/2009(H1N1)) |
| ADO12583 | HA | H1N1 | 2009/07/09 | (A/Chile/4181/2009(H1N1)) |
| ADO12584 | M1 | H1N1 | 2009/07/09 | (A/Chile/4181/2009(H1N1)) |
| ADO12585 | M2 | H1N1 | 2009/07/09 | (A/Chile/4181/2009(H1N1)) |
| ADO12586 | NA | H1N1 | 2009/07/09 | (A/Chile/4181/2009(H1N1)) |
| ADO12587 | NP | H1N1 | 2009/07/09 | (A/Chile/4181/2009(H1N1)) |
| ADO12588 | NS1 | H1N1 | 2009/07/09 | (A/Chile/4181/2009(H1N1)) |
| ADO12589 | NS2 | H1N1 | 2009/07/09 | (A/Chile/4181/2009(H1N1)) |
| ADO12590 | PA | H1N1 | 2009/07/09 | (A/Chile/4181/2009(H1N1)) |
| ADO12591 | PB1 | H1N1 | 2009/07/09 | (A/Chile/4181/2009(H1N1)) |
| ADO12592 | PB2 | H1N1 | 2009/07/09 | (A/Chile/4181/2009(H1N1)) |
| ADO12593 | HA | H1N1 | 2009/07/10 | (A/Chile/4182/2009(H1N1)) |
| ADO12594 | M1 | H1N1 | 2009/07/10 | (A/Chile/4182/2009(H1N1)) |
| ADO12595 | M2 | H1N1 | 2009/07/10 | (A/Chile/4182/2009(H1N1)) |
| ADO12596 | NA | H1N1 | 2009/07/10 | (A/Chile/4182/2009(H1N1)) |
| ADO12597 | NP | H1N1 | 2009/07/10 | (A/Chile/4182/2009(H1N1)) |
| ADO12598 | NS1 | H1N1 | 2009/07/10 | (A/Chile/4182/2009(H1N1)) |
| ADO12599 | NS2 | H1N1 | 2009/07/10 | (A/Chile/4182/2009(H1N1)) |
| ADO12600 | PA | H1N1 | 2009/07/10 | (A/Chile/4182/2009(H1N1)) |
| ADO12601 | PB1 | H1N1 | 2009/07/10 | (A/Chile/4182/2009(H1N1)) |
| ADO12602 | PB2 | H1N1 | 2009/07/10 | (A/Chile/4182/2009(H1N1)) |
| ADO12603 | HA | H1N1 | 2009/07/13 | (A/Chile/4257/2009(H1N1)) |
| ADO12604 | M1 | H1N1 | 2009/07/13 | (A/Chile/4257/2009(H1N1)) |
| ADO12605 | M2 | H1N1 | 2009/07/13 | (A/Chile/4257/2009(H1N1)) |
| ADO12606 | NA | H1N1 | 2009/07/13 | (A/Chile/4257/2009(H1N1)) |
| ADO12607 | NP | H1N1 | 2009/07/13 | (A/Chile/4257/2009(H1N1)) |
| ADO12608 | NS1 | H1N1 | 2009/07/13 | (A/Chile/4257/2009(H1N1)) |
| ADO12609 | NS2 | H1N1 | 2009/07/13 | (A/Chile/4257/2009(H1N1)) |
| ADO12610 | PA | H1N1 | 2009/07/13 | (A/Chile/4257/2009(H1N1)) |
| ADO12611 | PB1 | H1N1 | 2009/07/13 | (A/Chile/4257/2009(H1N1)) |
| ADO12612 | PB2 | H1N1 | 2009/07/13 | (A/Chile/4257/2009(H1N1)) |
| ADO12613 | HA | H1N1 | 2009/07/17 | (A/Chile/4406/2009(H1N1)) |
| ADO12614 | M1 | H1N1 | 2009/07/17 | (A/Chile/4406/2009(H1N1)) |
| ADO12615 | M2 | H1N1 | 2009/07/17 | (A/Chile/4406/2009(H1N1)) |
| ADO12616 | NA | H1N1 | 2009/07/17 | (A/Chile/4406/2009(H1N1)) |
| ADO12617 | NP | H1N1 | 2009/07/17 | (A/Chile/4406/2009(H1N1)) |
| ADO12618 | NS1 | H1N1 | 2009/07/17 | (A/Chile/4406/2009(H1N1)) |
| ADO12619 | NS2 | H1N1 | 2009/07/17 | (A/Chile/4406/2009(H1N1)) |
| ADO12620 | PA | H1N1 | 2009/07/17 | (A/Chile/4406/2009(H1N1)) |
| ADO12621 | PB1 | H1N1 | 2009/07/17 | (A/Chile/4406/2009(H1N1)) |
| ADO12622 | PB2 | H1N1 | 2009/07/17 | (A/Chile/4406/2009(H1N1)) |
| ADO12623 | HA | H1N1 | 2009/07/20 | (A/Chile/4438/2009(H1N1)) |
| ADO12624 | M1 | H1N1 | 2009/07/20 | (A/Chile/4438/2009(H1N1)) |
| ADO12625 | M2 | H1N1 | 2009/07/20 | (A/Chile/4438/2009(H1N1)) |
| ADO12626 | NA | H1N1 | 2009/07/20 | (A/Chile/4438/2009(H1N1)) |
| ADO12627 | NP | H1N1 | 2009/07/20 | (A/Chile/4438/2009(H1N1)) |
| ADO12628 | NS1 | H1N1 | 2009/07/20 | (A/Chile/4438/2009(H1N1)) |
| ADO12629 | NS2 | H1N1 | 2009/07/20 | (A/Chile/4438/2009(H1N1)) |
| ADO12630 | PA | H1N1 | 2009/07/20 | (A/Chile/4438/2009(H1N1)) |
| ADO12631 | PB1 | H1N1 | 2009/07/20 | (A/Chile/4438/2009(H1N1)) |
| ADO12632 | PB2 | H1N1 | 2009/07/20 | (A/Chile/4438/2009(H1N1)) |
| AEA73771 | HA | H1N1 | 2009/05/22 | (A/Chile/19/2009(H1N1)) |
| AEA73772 | M1 | H1N1 | 2009/05/22 | (A/Chile/19/2009(H1N1)) |
| AEA73773 | M2 | H1N1 | 2009/05/22 | (A/Chile/19/2009(H1N1)) |
| AEA73774 | NA | H1N1 | 2009/05/22 | (A/Chile/19/2009(H1N1)) |
| AEA73775 | NP | H1N1 | 2009/05/22 | (A/Chile/19/2009(H1N1)) |
| AEA73776 | NS1 | H1N1 | 2009/05/22 | (A/Chile/19/2009(H1N1)) |
| AEA73777 | NS2 | H1N1 | 2009/05/22 | (A/Chile/19/2009(H1N1)) |
| AEA73778 | PA | H1N1 | 2009/05/22 | (A/Chile/19/2009(H1N1)) |
| AEA73779 | PB1 | H1N1 | 2009/05/22 | (A/Chile/19/2009(H1N1)) |
| AEA73780 | PB2 | H1N1 | 2009/05/22 | (A/Chile/19/2009(H1N1)) |
| AEJ37853 | HA | H1N1 | 2010/05/11 | (A/Chile/6/2010(H1N1)) |
| AEJ37854 | M1 | H1N1 | 2010/05/11 | (A/Chile/6/2010(H1N1)) |
| AEJ37855 | M2 | H1N1 | 2010/05/11 | (A/Chile/6/2010(H1N1)) |
| AEJ37856 | NA | H1N1 | 2010/05/11 | (A/Chile/6/2010(H1N1)) |
| AEJ37857 | NP | H1N1 | 2010/05/11 | (A/Chile/6/2010(H1N1)) |
| AEJ37858 | NS1 | H1N1 | 2010/05/11 | (A/Chile/6/2010(H1N1)) |
| AEJ37859 | NS2 | H1N1 | 2010/05/11 | (A/Chile/6/2010(H1N1)) |
| AEJ37860 | PA | H1N1 | 2010/05/11 | (A/Chile/6/2010(H1N1)) |
| AEJ37861 | PB1 | H1N1 | 2010/05/11 | (A/Chile/6/2010(H1N1)) |
| AEJ37862 | PB2 | H1N1 | 2010/05/11 | (A/Chile/6/2010(H1N1)) |
| AEJ37863 | HA | H1N1 | 2010/06/22 | (A/Chile/15/2010(H1N1)) |
| AEJ37864 | M1 | H1N1 | 2010/06/22 | (A/Chile/15/2010(H1N1)) |
| AEJ37865 | M2 | H1N1 | 2010/06/22 | (A/Chile/15/2010(H1N1)) |
| AEJ37866 | NA | H1N1 | 2010/06/22 | (A/Chile/15/2010(H1N1)) |
| AEJ37867 | NP | H1N1 | 2010/06/22 | (A/Chile/15/2010(H1N1)) |
| AEJ37868 | NS1 | H1N1 | 2010/06/22 | (A/Chile/15/2010(H1N1)) |
| AEJ37869 | NS2 | H1N1 | 2010/06/22 | (A/Chile/15/2010(H1N1)) |
| AEJ37870 | PA | H1N1 | 2010/06/22 | (A/Chile/15/2010(H1N1)) |
| AEJ37871 | PB1 | H1N1 | 2010/06/22 | (A/Chile/15/2010(H1N1)) |
| AEJ37872 | PB2 | H1N1 | 2010/06/22 | (A/Chile/15/2010(H1N1)) |
| AEJ37873 | HA | H1N1 | 2010/07/01 | (A/Chile/19/2010(H1N1)) |
| AEJ37874 | M1 | H1N1 | 2010/07/01 | (A/Chile/19/2010(H1N1)) |
| AEJ37875 | M2 | H1N1 | 2010/07/01 | (A/Chile/19/2010(H1N1)) |
| AEJ37876 | NA | H1N1 | 2010/07/01 | (A/Chile/19/2010(H1N1)) |
| AEJ37877 | NP | H1N1 | 2010/07/01 | (A/Chile/19/2010(H1N1)) |
| AEJ37878 | NS1 | H1N1 | 2010/07/01 | (A/Chile/19/2010(H1N1)) |
| AEJ37879 | NS2 | H1N1 | 2010/07/01 | (A/Chile/19/2010(H1N1)) |
| AEJ37880 | PA | H1N1 | 2010/07/01 | (A/Chile/19/2010(H1N1)) |
| AEJ37881 | PB1 | H1N1 | 2010/07/01 | (A/Chile/19/2010(H1N1)) |
| AEJ37882 | PB2 | H1N1 | 2010/07/01 | (A/Chile/19/2010(H1N1)) |
| AEJ37883 | HA | H1N1 | 2010/07/17 | (A/Chile/23/2010(H1N1)) |
| AEJ37884 | M1 | H1N1 | 2010/07/17 | (A/Chile/23/2010(H1N1)) |
| AEJ37885 | M2 | H1N1 | 2010/07/17 | (A/Chile/23/2010(H1N1)) |
| AEJ37886 | NA | H1N1 | 2010/07/17 | (A/Chile/23/2010(H1N1)) |
| AEJ37887 | NP | H1N1 | 2010/07/17 | (A/Chile/23/2010(H1N1)) |
| AEJ37888 | NS1 | H1N1 | 2010/07/17 | (A/Chile/23/2010(H1N1)) |
| AEJ37889 | NS2 | H1N1 | 2010/07/17 | (A/Chile/23/2010(H1N1)) |
| AEJ37890 | PA | H1N1 | 2010/07/17 | (A/Chile/23/2010(H1N1)) |
| AEJ37891 | PB1 | H1N1 | 2010/07/17 | (A/Chile/23/2010(H1N1)) |
| AEJ37892 | PB2 | H1N1 | 2010/07/17 | (A/Chile/23/2010(H1N1)) |
| AEJ37893 | HA | H1N1 | 2010/07/26 | (A/Chile/28/2010(H1N1)) |
| AEJ37894 | M1 | H1N1 | 2010/07/26 | (A/Chile/28/2010(H1N1)) |
| AEJ37895 | M2 | H1N1 | 2010/07/26 | (A/Chile/28/2010(H1N1)) |
| AEJ37896 | NA | H1N1 | 2010/07/26 | (A/Chile/28/2010(H1N1)) |
| AEJ37897 | NP | H1N1 | 2010/07/26 | (A/Chile/28/2010(H1N1)) |
| AEJ37898 | NS1 | H1N1 | 2010/07/26 | (A/Chile/28/2010(H1N1)) |
| AEJ37899 | NS2 | H1N1 | 2010/07/26 | (A/Chile/28/2010(H1N1)) |
| AEJ37900 | PA | H1N1 | 2010/07/26 | (A/Chile/28/2010(H1N1)) |
| AEJ37901 | PB1 | H1N1 | 2010/07/26 | (A/Chile/28/2010(H1N1)) |
| AEJ37902 | PB2 | H1N1 | 2010/07/26 | (A/Chile/28/2010(H1N1)) |
| AEJ37903 | HA | H1N1 | 2010/07/26 | (A/Chile/29/2010(H1N1)) |
| AEJ37904 | M1 | H1N1 | 2010/07/26 | (A/Chile/29/2010(H1N1)) |
| AEJ37905 | M2 | H1N1 | 2010/07/26 | (A/Chile/29/2010(H1N1)) |
| AEJ37906 | NA | H1N1 | 2010/07/26 | (A/Chile/29/2010(H1N1)) |
| AEJ37907 | NP | H1N1 | 2010/07/26 | (A/Chile/29/2010(H1N1)) |
| AEJ37908 | NS1 | H1N1 | 2010/07/26 | (A/Chile/29/2010(H1N1)) |
| AEJ37909 | NS2 | H1N1 | 2010/07/26 | (A/Chile/29/2010(H1N1)) |
| AEJ37910 | PA | H1N1 | 2010/07/26 | (A/Chile/29/2010(H1N1)) |
| AEJ37911 | PB1 | H1N1 | 2010/07/26 | (A/Chile/29/2010(H1N1)) |
| AEJ37912 | PB2 | H1N1 | 2010/07/26 | (A/Chile/29/2010(H1N1)) |
| AEJ37913 | HA | H1N1 | 2010/07/28 | (A/Chile/34/2010(H1N1)) |
| AEJ37914 | M1 | H1N1 | 2010/07/28 | (A/Chile/34/2010(H1N1)) |
| AEJ37915 | M2 | H1N1 | 2010/07/28 | (A/Chile/34/2010(H1N1)) |
| AEJ37916 | NA | H1N1 | 2010/07/28 | (A/Chile/34/2010(H1N1)) |
| AEJ37917 | NP | H1N1 | 2010/07/28 | (A/Chile/34/2010(H1N1)) |
| AEJ37918 | NS1 | H1N1 | 2010/07/28 | (A/Chile/34/2010(H1N1)) |
| AEJ37919 | NS2 | H1N1 | 2010/07/28 | (A/Chile/34/2010(H1N1)) |
| AEJ37920 | PA | H1N1 | 2010/07/28 | (A/Chile/34/2010(H1N1)) |
| AEJ37921 | PB1 | H1N1 | 2010/07/28 | (A/Chile/34/2010(H1N1)) |
| AEJ37922 | PB2 | H1N1 | 2010/07/28 | (A/Chile/34/2010(H1N1)) |
| AEJ37923 | HA | H1N1 | 2010/07/28 | (A/Chile/37/2010(H1N1)) |
| AEJ37924 | M1 | H1N1 | 2010/07/28 | (A/Chile/37/2010(H1N1)) |
| AEJ37925 | M2 | H1N1 | 2010/07/28 | (A/Chile/37/2010(H1N1)) |
| AEJ37926 | NA | H1N1 | 2010/07/28 | (A/Chile/37/2010(H1N1)) |
| AEJ37927 | NP | H1N1 | 2010/07/28 | (A/Chile/37/2010(H1N1)) |
| AEJ37928 | NS1 | H1N1 | 2010/07/28 | (A/Chile/37/2010(H1N1)) |
| AEJ37929 | NS2 | H1N1 | 2010/07/28 | (A/Chile/37/2010(H1N1)) |
| AEJ37930 | PA | H1N1 | 2010/07/28 | (A/Chile/37/2010(H1N1)) |
| AEJ37931 | PB1 | H1N1 | 2010/07/28 | (A/Chile/37/2010(H1N1)) |
| AEJ37932 | PB2 | H1N1 | 2010/07/28 | (A/Chile/37/2010(H1N1)) |
| AEJ37933 | HA | H1N1 | 2010/08/09 | (A/Chile/54/2010(H1N1)) |
| AEJ37934 | M1 | H1N1 | 2010/08/09 | (A/Chile/54/2010(H1N1)) |
| AEJ37935 | M2 | H1N1 | 2010/08/09 | (A/Chile/54/2010(H1N1)) |
| AEJ37936 | NA | H1N1 | 2010/08/09 | (A/Chile/54/2010(H1N1)) |
| AEJ37937 | NP | H1N1 | 2010/08/09 | (A/Chile/54/2010(H1N1)) |
| AEJ37938 | NS1 | H1N1 | 2010/08/09 | (A/Chile/54/2010(H1N1)) |
| AEJ37939 | NS2 | H1N1 | 2010/08/09 | (A/Chile/54/2010(H1N1)) |
| AEJ37940 | PA | H1N1 | 2010/08/09 | (A/Chile/54/2010(H1N1)) |
| AEJ37941 | PB1 | H1N1 | 2010/08/09 | (A/Chile/54/2010(H1N1)) |
| AEJ37942 | PB2 | H1N1 | 2010/08/09 | (A/Chile/54/2010(H1N1)) |
| AEJ37943 | HA | H1N1 | 2010/08/09 | (A/Chile/52/2010(H1N1)) |
| AEJ37944 | M1 | H1N1 | 2010/08/09 | (A/Chile/52/2010(H1N1)) |
| AEJ37945 | M2 | H1N1 | 2010/08/09 | (A/Chile/52/2010(H1N1)) |
| AEJ37946 | NA | H1N1 | 2010/08/09 | (A/Chile/52/2010(H1N1)) |
| AEJ37947 | NP | H1N1 | 2010/08/09 | (A/Chile/52/2010(H1N1)) |
| AEJ37948 | NS1 | H1N1 | 2010/08/09 | (A/Chile/52/2010(H1N1)) |
| AEJ37949 | NS2 | H1N1 | 2010/08/09 | (A/Chile/52/2010(H1N1)) |
| AEJ37950 | PA | H1N1 | 2010/08/09 | (A/Chile/52/2010(H1N1)) |
| AEJ37951 | PB1 | H1N1 | 2010/08/09 | (A/Chile/52/2010(H1N1)) |
| AEJ37952 | PB2 | H1N1 | 2010/08/09 | (A/Chile/52/2010(H1N1)) |
| AEJ37953 | HA | H1N1 | 2010/08/10 | (A/Chile/58/2010(H1N1)) |
| AEJ37954 | M1 | H1N1 | 2010/08/10 | (A/Chile/58/2010(H1N1)) |
| AEJ37955 | M2 | H1N1 | 2010/08/10 | (A/Chile/58/2010(H1N1)) |
| AEJ37956 | NA | H1N1 | 2010/08/10 | (A/Chile/58/2010(H1N1)) |
| AEJ37957 | NP | H1N1 | 2010/08/10 | (A/Chile/58/2010(H1N1)) |
| AEJ37958 | NS1 | H1N1 | 2010/08/10 | (A/Chile/58/2010(H1N1)) |
| AEJ37959 | NS2 | H1N1 | 2010/08/10 | (A/Chile/58/2010(H1N1)) |
| AEJ37960 | PA | H1N1 | 2010/08/10 | (A/Chile/58/2010(H1N1)) |
| AEJ37961 | PB1 | H1N1 | 2010/08/10 | (A/Chile/58/2010(H1N1)) |
| AEJ37962 | PB2 | H1N1 | 2010/08/10 | (A/Chile/58/2010(H1N1)) |
| AEJ37963 | HA | H1N1 | 2010/08/16 | (A/Chile/71/2010(H1N1)) |
| AEJ37964 | M1 | H1N1 | 2010/08/16 | (A/Chile/71/2010(H1N1)) |
| AEJ37965 | M2 | H1N1 | 2010/08/16 | (A/Chile/71/2010(H1N1)) |
| AEJ37966 | NA | H1N1 | 2010/08/16 | (A/Chile/71/2010(H1N1)) |
| AEJ37967 | NP | H1N1 | 2010/08/16 | (A/Chile/71/2010(H1N1)) |
| AEJ37968 | NS1 | H1N1 | 2010/08/16 | (A/Chile/71/2010(H1N1)) |
| AEJ37969 | NS2 | H1N1 | 2010/08/16 | (A/Chile/71/2010(H1N1)) |
| AEJ37970 | PA | H1N1 | 2010/08/16 | (A/Chile/71/2010(H1N1)) |
| AEJ37971 | PB1 | H1N1 | 2010/08/16 | (A/Chile/71/2010(H1N1)) |
| AEJ37972 | PB2 | H1N1 | 2010/08/16 | (A/Chile/71/2010(H1N1)) |
| AEJ37973 | HA | H1N1 | 2010/08/16 | (A/Chile/72/2010(H1N1)) |
| AEJ37974 | M1 | H1N1 | 2010/08/16 | (A/Chile/72/2010(H1N1)) |
| AEJ37975 | M2 | H1N1 | 2010/08/16 | (A/Chile/72/2010(H1N1)) |
| AEJ37976 | NA | H1N1 | 2010/08/16 | (A/Chile/72/2010(H1N1)) |
| AEJ37977 | NP | H1N1 | 2010/08/16 | (A/Chile/72/2010(H1N1)) |
| AEJ37978 | NS1 | H1N1 | 2010/08/16 | (A/Chile/72/2010(H1N1)) |
| AEJ37979 | NS2 | H1N1 | 2010/08/16 | (A/Chile/72/2010(H1N1)) |
| AEJ37980 | PA | H1N1 | 2010/08/16 | (A/Chile/72/2010(H1N1)) |
| AEJ37981 | PB1 | H1N1 | 2010/08/16 | (A/Chile/72/2010(H1N1)) |
| AEJ37982 | PB2 | H1N1 | 2010/08/16 | (A/Chile/72/2010(H1N1)) |
| AEJ37983 | HA | H1N1 | 2010/08/17 | (A/Chile/78/2010(H1N1)) |
| AEJ37984 | M1 | H1N1 | 2010/08/17 | (A/Chile/78/2010(H1N1)) |
| AEJ37985 | M2 | H1N1 | 2010/08/17 | (A/Chile/78/2010(H1N1)) |
| AEJ37986 | NA | H1N1 | 2010/08/17 | (A/Chile/78/2010(H1N1)) |
| AEJ37987 | NP | H1N1 | 2010/08/17 | (A/Chile/78/2010(H1N1)) |
| AEJ37988 | NS1 | H1N1 | 2010/08/17 | (A/Chile/78/2010(H1N1)) |
| AEJ37989 | NS2 | H1N1 | 2010/08/17 | (A/Chile/78/2010(H1N1)) |
| AEJ37990 | PA | H1N1 | 2010/08/17 | (A/Chile/78/2010(H1N1)) |
| AEJ37991 | PB1 | H1N1 | 2010/08/17 | (A/Chile/78/2010(H1N1)) |
| AEJ37992 | PB2 | H1N1 | 2010/08/17 | (A/Chile/78/2010(H1N1)) |
| AEK21426 | HA | H1N1 | 2010/08/11 | (A/Chile/61/2010(H1N1)) |
| AEK21427 | M1 | H1N1 | 2010/08/11 | (A/Chile/61/2010(H1N1)) |
| AEK21428 | M2 | H1N1 | 2010/08/11 | (A/Chile/61/2010(H1N1)) |
| AEK21429 | NA | H1N1 | 2010/08/11 | (A/Chile/61/2010(H1N1)) |
| AEK21430 | NP | H1N1 | 2010/08/11 | (A/Chile/61/2010(H1N1)) |
| AEK21431 | NS1 | H1N1 | 2010/08/11 | (A/Chile/61/2010(H1N1)) |
| AEK21432 | NS2 | H1N1 | 2010/08/11 | (A/Chile/61/2010(H1N1)) |
| AEK21433 | PA | H1N1 | 2010/08/11 | (A/Chile/61/2010(H1N1)) |
| AEK21434 | PB1 | H1N1 | 2010/08/11 | (A/Chile/61/2010(H1N1)) |
| AEK21435 | PB2 | H1N1 | 2010/08/11 | (A/Chile/61/2010(H1N1)) |
| AEK21436 | HA | H1N1 | 2010/08/11 | (A/Chile/64/2010(H1N1)) |
| AEK21437 | M1 | H1N1 | 2010/08/11 | (A/Chile/64/2010(H1N1)) |
| AEK21438 | M2 | H1N1 | 2010/08/11 | (A/Chile/64/2010(H1N1)) |
| AEK21439 | NA | H1N1 | 2010/08/11 | (A/Chile/64/2010(H1N1)) |
| AEK21440 | NP | H1N1 | 2010/08/11 | (A/Chile/64/2010(H1N1)) |
| AEK21441 | NS1 | H1N1 | 2010/08/11 | (A/Chile/64/2010(H1N1)) |
| AEK21442 | NS2 | H1N1 | 2010/08/11 | (A/Chile/64/2010(H1N1)) |
| AEK21443 | PA | H1N1 | 2010/08/11 | (A/Chile/64/2010(H1N1)) |
| AEK21444 | PB1 | H1N1 | 2010/08/11 | (A/Chile/64/2010(H1N1)) |
| AEK21445 | PB2 | H1N1 | 2010/08/11 | (A/Chile/64/2010(H1N1)) |
| AEK21446 | HA | H1N1 | 2010/08/19 | (A/Chile/87/2010(H1N1)) |
| AEK21447 | M1 | H1N1 | 2010/08/19 | (A/Chile/87/2010(H1N1)) |
| AEK21448 | M2 | H1N1 | 2010/08/19 | (A/Chile/87/2010(H1N1)) |
| AEK21449 | NA | H1N1 | 2010/08/19 | (A/Chile/87/2010(H1N1)) |
| AEK21450 | NP | H1N1 | 2010/08/19 | (A/Chile/87/2010(H1N1)) |
| AEK21451 | NS1 | H1N1 | 2010/08/19 | (A/Chile/87/2010(H1N1)) |
| AEK21452 | NS2 | H1N1 | 2010/08/19 | (A/Chile/87/2010(H1N1)) |
| AEK21453 | PA | H1N1 | 2010/08/19 | (A/Chile/87/2010(H1N1)) |
| AEK21454 | PB1 | H1N1 | 2010/08/19 | (A/Chile/87/2010(H1N1)) |
| AEK21455 | PB2 | H1N1 | 2010/08/19 | (A/Chile/87/2010(H1N1)) |
| AEK21456 | HA | H1N1 | 2010/08/20 | (A/Chile/94/2010(H1N1)) |
| AEK21457 | M1 | H1N1 | 2010/08/20 | (A/Chile/94/2010(H1N1)) |
| AEK21458 | M2 | H1N1 | 2010/08/20 | (A/Chile/94/2010(H1N1)) |
| AEK21459 | NA | H1N1 | 2010/08/20 | (A/Chile/94/2010(H1N1)) |
| AEK21460 | NP | H1N1 | 2010/08/20 | (A/Chile/94/2010(H1N1)) |
| AEK21461 | NS1 | H1N1 | 2010/08/20 | (A/Chile/94/2010(H1N1)) |
| AEK21462 | NS2 | H1N1 | 2010/08/20 | (A/Chile/94/2010(H1N1)) |
| AEK21463 | PA | H1N1 | 2010/08/20 | (A/Chile/94/2010(H1N1)) |
| AEK21464 | PB1 | H1N1 | 2010/08/20 | (A/Chile/94/2010(H1N1)) |
| AEK21465 | PB2 | H1N1 | 2010/08/20 | (A/Chile/94/2010(H1N1)) |
| AEK21466 | HA | H1N1 | 2010/08/24 | (A/Chile/106/2010(H1N1)) |
| AEK21467 | M1 | H1N1 | 2010/08/24 | (A/Chile/106/2010(H1N1)) |
| AEK21468 | M2 | H1N1 | 2010/08/24 | (A/Chile/106/2010(H1N1)) |
| AEK21469 | NA | H1N1 | 2010/08/24 | (A/Chile/106/2010(H1N1)) |
| AEK21470 | NP | H1N1 | 2010/08/24 | (A/Chile/106/2010(H1N1)) |
| AEK21471 | NS1 | H1N1 | 2010/08/24 | (A/Chile/106/2010(H1N1)) |
| AEK21472 | NS2 | H1N1 | 2010/08/24 | (A/Chile/106/2010(H1N1)) |
| AEK21473 | PA | H1N1 | 2010/08/24 | (A/Chile/106/2010(H1N1)) |
| AEK21474 | PB1 | H1N1 | 2010/08/24 | (A/Chile/106/2010(H1N1)) |
| AEK21475 | PB2 | H1N1 | 2010/08/24 | (A/Chile/106/2010(H1N1)) |
| AEK21476 | HA | H1N1 | 2010/08/25 | (A/Chile/109/2010(H1N1)) |
| AEK21477 | M1 | H1N1 | 2010/08/25 | (A/Chile/109/2010(H1N1)) |
| AEK21478 | M2 | H1N1 | 2010/08/25 | (A/Chile/109/2010(H1N1)) |
| AEK21479 | NA | H1N1 | 2010/08/25 | (A/Chile/109/2010(H1N1)) |
| AEK21480 | NP | H1N1 | 2010/08/25 | (A/Chile/109/2010(H1N1)) |
| AEK21481 | NS1 | H1N1 | 2010/08/25 | (A/Chile/109/2010(H1N1)) |
| AEK21482 | NS2 | H1N1 | 2010/08/25 | (A/Chile/109/2010(H1N1)) |
| AEK21483 | PA | H1N1 | 2010/08/25 | (A/Chile/109/2010(H1N1)) |
| AEK21484 | PB1 | H1N1 | 2010/08/25 | (A/Chile/109/2010(H1N1)) |
| AEK21485 | PB2 | H1N1 | 2010/08/25 | (A/Chile/109/2010(H1N1)) |
| AEK21486 | HA | H1N1 | 2010 | (A/Chile/115/2010(H1N1)) |
| AEK21487 | M1 | H1N1 | 2010 | (A/Chile/115/2010(H1N1)) |
| AEK21488 | M2 | H1N1 | 2010 | (A/Chile/115/2010(H1N1)) |
| AEK21489 | NA | H1N1 | 2010 | (A/Chile/115/2010(H1N1)) |
| AEK21490 | NP | H1N1 | 2010 | (A/Chile/115/2010(H1N1)) |
| AEK21491 | NS1 | H1N1 | 2010 | (A/Chile/115/2010(H1N1)) |
| AEK21492 | NS2 | H1N1 | 2010 | (A/Chile/115/2010(H1N1)) |
| AEK21493 | PA | H1N1 | 2010 | (A/Chile/115/2010(H1N1)) |
| AEK21494 | PB1 | H1N1 | 2010 | (A/Chile/115/2010(H1N1)) |
| AEK21495 | PB2 | H1N1 | 2010 | (A/Chile/115/2010(H1N1)) |
| AEN55398 | HA | H1N1 | 2010/08/19 | (A/Chile/89/2010(H1N1)) |
| AEN55399 | M1 | H1N1 | 2010/08/19 | (A/Chile/89/2010(H1N1)) |
| AEN55400 | M2 | H1N1 | 2010/08/19 | (A/Chile/89/2010(H1N1)) |
| AEN55401 | NA | H1N1 | 2010/08/19 | (A/Chile/89/2010(H1N1)) |
| AEN55402 | NP | H1N1 | 2010/08/19 | (A/Chile/89/2010(H1N1)) |
| AEN55403 | NS1 | H1N1 | 2010/08/19 | (A/Chile/89/2010(H1N1)) |
| AEN55404 | NS2 | H1N1 | 2010/08/19 | (A/Chile/89/2010(H1N1)) |
| AEN55405 | PA | H1N1 | 2010/08/19 | (A/Chile/89/2010(H1N1)) |
| AEN55406 | PB1 | H1N1 | 2010/08/19 | (A/Chile/89/2010(H1N1)) |
| AEN55407 | PB2 | H1N1 | 2010/08/19 | (A/Chile/89/2010(H1N1)) |
| AEN69426 | HA | H1N1 | 2010/08/16 | (A/Chile/70/2010(H1N1)) |
| AEN69427 | M1 | H1N1 | 2010/08/16 | (A/Chile/70/2010(H1N1)) |
| AEN69428 | M2 | H1N1 | 2010/08/16 | (A/Chile/70/2010(H1N1)) |
| AEN69429 | NA | H1N1 | 2010/08/16 | (A/Chile/70/2010(H1N1)) |
| AEN69430 | NP | H1N1 | 2010/08/16 | (A/Chile/70/2010(H1N1)) |
| AEN69431 | NS1 | H1N1 | 2010/08/16 | (A/Chile/70/2010(H1N1)) |
| AEN69432 | NS2 | H1N1 | 2010/08/16 | (A/Chile/70/2010(H1N1)) |
| AEN69433 | PB1 | H1N1 | 2010/08/16 | (A/Chile/70/2010(H1N1)) |
| AEN69434 | PB2 | H1N1 | 2010/08/16 | (A/Chile/70/2010(H1N1)) |
| AIE52823 | M1 | H1N1 | 2013/06/11 | (A/Santiago/p13d0/2013(H1N1)) |
| AIE52824 | M2 | H1N1 | 2013/06/11 | (A/Santiago/p13d0/2013(H1N1)) |
| AIE52826 | NP | H1N1 | 2013/06/11 | (A/Santiago/p13d0/2013(H1N1)) |
| AIE52827 | NS1 | H1N1 | 2013/06/11 | (A/Santiago/p13d0/2013(H1N1)) |
| AIE52828 | NS2 | H1N1 | 2013/06/11 | (A/Santiago/p13d0/2013(H1N1)) |
| AIE52829 | PA-X | H1N1 | 2013/06/11 | (A/Santiago/p13d0/2013(H1N1)) |
| AIE52830 | PA | H1N1 | 2013/06/11 | (A/Santiago/p13d0/2013(H1N1)) |
| AIE52831 | PB2 | H1N1 | 2013/06/11 | (A/Santiago/p13d0/2013(H1N1)) |
| AIE52832 | HA | H3N2 | 2013/08/09 | (A/Santiago/p34d2/2013(H3N2)) |
| AIE52833 | M1 | H3N2 | 2013/08/09 | (A/Santiago/p34d2/2013(H3N2)) |
| AIE52834 | M2 | H3N2 | 2013/08/09 | (A/Santiago/p34d2/2013(H3N2)) |
| AIE52836 | NP | H3N2 | 2013/08/09 | (A/Santiago/p34d2/2013(H3N2)) |
| AIE52837 | PA-X | H3N2 | 2013/08/09 | (A/Santiago/p34d2/2013(H3N2)) |
| AIE52838 | PA | H3N2 | 2013/08/09 | (A/Santiago/p34d2/2013(H3N2)) |
| AIE52839 | PB1 | H3N2 | 2013/08/09 | (A/Santiago/p34d2/2013(H3N2)) |
| AIE52840 | PB1-F2 | H3N2 | 2013/08/09 | (A/Santiago/p34d2/2013(H3N2)) |
| AIE52842 | HA | H1N1 | 2013/07/09 | (A/Santiago/p25d3/2013(H1N1)) |
| AIE52845 | NA | H1N1 | 2013/07/09 | (A/Santiago/p25d3/2013(H1N1)) |
| AIE52846 | NP | H1N1 | 2013/07/09 | (A/Santiago/p25d3/2013(H1N1)) |
| AIE52847 | NS1 | H1N1 | 2013/07/09 | (A/Santiago/p25d3/2013(H1N1)) |
| AIE52848 | NS2 | H1N1 | 2013/07/09 | (A/Santiago/p25d3/2013(H1N1)) |
| AIE52849 | PA-X | H1N1 | 2013/07/09 | (A/Santiago/p25d3/2013(H1N1)) |
| AIE52850 | PA | H1N1 | 2013/07/09 | (A/Santiago/p25d3/2013(H1N1)) |
| AIE52851 | PB1 | H1N1 | 2013/07/09 | (A/Santiago/p25d3/2013(H1N1)) |
| AIE52852 | PB2 | H1N1 | 2013/07/09 | (A/Santiago/p25d3/2013(H1N1)) |
| AIE52853 | HA | H3N2 | 2012/06/22 | (A/Santiago/p5d2/2012(H3N2)) |
| AIE52854 | M1 | H3N2 | 2012/06/22 | (A/Santiago/p5d2/2012(H3N2)) |
| AIE52855 | M2 | H3N2 | 2012/06/22 | (A/Santiago/p5d2/2012(H3N2)) |
| AIE52856 | NA | H3N2 | 2012/06/22 | (A/Santiago/p5d2/2012(H3N2)) |
| AIE52857 | NP | H3N2 | 2012/06/22 | (A/Santiago/p5d2/2012(H3N2)) |
| AIE52858 | NS1 | H3N2 | 2012/06/22 | (A/Santiago/p5d2/2012(H3N2)) |
| AIE52859 | NS2 | H3N2 | 2012/06/22 | (A/Santiago/p5d2/2012(H3N2)) |
| AIE52860 | PA-X | H3N2 | 2012/06/22 | (A/Santiago/p5d2/2012(H3N2)) |
| AIE52861 | PA | H3N2 | 2012/06/22 | (A/Santiago/p5d2/2012(H3N2)) |
| AIE52862 | PB1 | H3N2 | 2012/06/22 | (A/Santiago/p5d2/2012(H3N2)) |
| AIE52863 | PB2 | H3N2 | 2012/06/22 | (A/Santiago/p5d2/2012(H3N2)) |
| AIE52864 | HA | H3N2 | 2012/06/28 | (A/Santiago/op5d1/2012(H3N2)) |
| AIE52865 | M1 | H3N2 | 2012/06/28 | (A/Santiago/op5d1/2012(H3N2)) |
| AIE52866 | M2 | H3N2 | 2012/06/28 | (A/Santiago/op5d1/2012(H3N2)) |
| AIE52867 | NA | H3N2 | 2012/06/28 | (A/Santiago/op5d1/2012(H3N2)) |
| AIE52868 | NP | H3N2 | 2012/06/28 | (A/Santiago/op5d1/2012(H3N2)) |
| AIE52869 | NS1 | H3N2 | 2012/06/28 | (A/Santiago/op5d1/2012(H3N2)) |
| AIE52870 | NS2 | H3N2 | 2012/06/28 | (A/Santiago/op5d1/2012(H3N2)) |
| AIE52871 | PA-X | H3N2 | 2012/06/28 | (A/Santiago/op5d1/2012(H3N2)) |
| AIE52872 | PA | H3N2 | 2012/06/28 | (A/Santiago/op5d1/2012(H3N2)) |
| AIE52873 | PB1 | H3N2 | 2012/06/28 | (A/Santiago/op5d1/2012(H3N2)) |
| AIE52874 | PB2 | H3N2 | 2012/06/28 | (A/Santiago/op5d1/2012(H3N2)) |
| AIE52875 | M1 |  | 2013/10/08 | (A/Santiago/p38d21/2013) |
| AIE52876 | M2 |  | 2013/10/08 | (A/Santiago/p38d21/2013) |
| AIE52878 | M1 | H3N2 | 2012/06/04 | (A/Santiago/op17d5/2012(H3N2)) |
| AIE52879 | M2 | H3N2 | 2012/06/04 | (A/Santiago/op17d5/2012(H3N2)) |
| AIE52885 | HA | H3N2 | 2013/08/30 | (A/Santiago/p36d1/2013(H3N2)) |
| AIE52886 | M1 | H3N2 | 2013/08/30 | (A/Santiago/p36d1/2013(H3N2)) |
| AIE52887 | M2 | H3N2 | 2013/08/30 | (A/Santiago/p36d1/2013(H3N2)) |
| AIE52888 | NA | H3N2 | 2013/08/30 | (A/Santiago/p36d1/2013(H3N2)) |
| AIE52889 | NP | H3N2 | 2013/08/30 | (A/Santiago/p36d1/2013(H3N2)) |
| AIE52890 | NS1 | H3N2 | 2013/08/30 | (A/Santiago/p36d1/2013(H3N2)) |
| AIE52891 | NS2 | H3N2 | 2013/08/30 | (A/Santiago/p36d1/2013(H3N2)) |
| AIE52892 | PA-X | H3N2 | 2013/08/30 | (A/Santiago/p36d1/2013(H3N2)) |
| AIE52893 | PA | H3N2 | 2013/08/30 | (A/Santiago/p36d1/2013(H3N2)) |
| AIE52894 | PB1 | H3N2 | 2013/08/30 | (A/Santiago/p36d1/2013(H3N2)) |
| AIE52895 | PB1-F2 | H3N2 | 2013/08/30 | (A/Santiago/p36d1/2013(H3N2)) |
| AIE52896 | PB2 | H3N2 | 2013/08/30 | (A/Santiago/p36d1/2013(H3N2)) |
| AIE52897 | HA | H3N2 | 2013/08/29 | (A/Santiago/p36d0/2013(H3N2)) |
| AIE52898 | M1 | H3N2 | 2013/08/29 | (A/Santiago/p36d0/2013(H3N2)) |
| AIE52899 | M2 | H3N2 | 2013/08/29 | (A/Santiago/p36d0/2013(H3N2)) |
| AIE52900 | NA | H3N2 | 2013/08/29 | (A/Santiago/p36d0/2013(H3N2)) |
| AIE52901 | NP | H3N2 | 2013/08/29 | (A/Santiago/p36d0/2013(H3N2)) |
| AIE52902 | NS1 | H3N2 | 2013/08/29 | (A/Santiago/p36d0/2013(H3N2)) |
| AIE52903 | NS2 | H3N2 | 2013/08/29 | (A/Santiago/p36d0/2013(H3N2)) |
| AIE52904 | PA-X | H3N2 | 2013/08/29 | (A/Santiago/p36d0/2013(H3N2)) |
| AIE52905 | PA | H3N2 | 2013/08/29 | (A/Santiago/p36d0/2013(H3N2)) |
| AIE52906 | PB1 | H3N2 | 2013/08/29 | (A/Santiago/p36d0/2013(H3N2)) |
| AIE52907 | PB1-F2 | H3N2 | 2013/08/29 | (A/Santiago/p36d0/2013(H3N2)) |
| AIE52908 | PB2 | H3N2 | 2013/08/29 | (A/Santiago/p36d0/2013(H3N2)) |
| AIE52909 | HA | H3N2 | 2012/07/06 | (A/Santiago/p19d2/2012(H3N2)) |
| AIE52910 | M1 | H3N2 | 2012/07/06 | (A/Santiago/p19d2/2012(H3N2)) |
| AIE52911 | M2 | H3N2 | 2012/07/06 | (A/Santiago/p19d2/2012(H3N2)) |
| AIE52912 | NA | H3N2 | 2012/07/06 | (A/Santiago/p19d2/2012(H3N2)) |
| AIE52913 | NP | H3N2 | 2012/07/06 | (A/Santiago/p19d2/2012(H3N2)) |
| AIE52914 | NS1 | H3N2 | 2012/07/06 | (A/Santiago/p19d2/2012(H3N2)) |
| AIE52915 | NS2 | H3N2 | 2012/07/06 | (A/Santiago/p19d2/2012(H3N2)) |
| AIE52916 | PA-X | H3N2 | 2012/07/06 | (A/Santiago/p19d2/2012(H3N2)) |
| AIE52917 | PA | H3N2 | 2012/07/06 | (A/Santiago/p19d2/2012(H3N2)) |
| AIE52918 | PB1 | H3N2 | 2012/07/06 | (A/Santiago/p19d2/2012(H3N2)) |
| AIE52919 | PB2 | H3N2 | 2012/07/06 | (A/Santiago/p19d2/2012(H3N2)) |
| AIE52920 | HA | H3N2 | 2012/10/31 | (A/Santiago/p37d1/2012(H3N2)) |
| AIE52921 | M1 | H3N2 | 2012/10/31 | (A/Santiago/p37d1/2012(H3N2)) |
| AIE52922 | M2 | H3N2 | 2012/10/31 | (A/Santiago/p37d1/2012(H3N2)) |
| AIE52923 | NA | H3N2 | 2012/10/31 | (A/Santiago/p37d1/2012(H3N2)) |
| AIE52924 | NP | H3N2 | 2012/10/31 | (A/Santiago/p37d1/2012(H3N2)) |
| AIE52925 | NS1 | H3N2 | 2012/10/31 | (A/Santiago/p37d1/2012(H3N2)) |
| AIE52926 | NS2 | H3N2 | 2012/10/31 | (A/Santiago/p37d1/2012(H3N2)) |
| AIE52927 | PA-X | H3N2 | 2012/10/31 | (A/Santiago/p37d1/2012(H3N2)) |
| AIE52928 | PA | H3N2 | 2012/10/31 | (A/Santiago/p37d1/2012(H3N2)) |
| AIE52929 | PB1 | H3N2 | 2012/10/31 | (A/Santiago/p37d1/2012(H3N2)) |
| AIE52930 | PB1-F2 | H3N2 | 2012/10/31 | (A/Santiago/p37d1/2012(H3N2)) |
| AIE52931 | PB2 | H3N2 | 2012/10/31 | (A/Santiago/p37d1/2012(H3N2)) |
| AIE52932 | HA | H1N1 | 2013/06/19 | (A/Santiago/p18d0/2013(H1N1)) |
| AIE52933 | M1 | H1N1 | 2013/06/19 | (A/Santiago/p18d0/2013(H1N1)) |
| AIE52934 | M2 | H1N1 | 2013/06/19 | (A/Santiago/p18d0/2013(H1N1)) |
| AIE52935 | NA | H1N1 | 2013/06/19 | (A/Santiago/p18d0/2013(H1N1)) |
| AIE52936 | NP | H1N1 | 2013/06/19 | (A/Santiago/p18d0/2013(H1N1)) |
| AIE52937 | NS1 | H1N1 | 2013/06/19 | (A/Santiago/p18d0/2013(H1N1)) |
| AIE52938 | NS2 | H1N1 | 2013/06/19 | (A/Santiago/p18d0/2013(H1N1)) |
| AIE52939 | PA-X | H1N1 | 2013/06/19 | (A/Santiago/p18d0/2013(H1N1)) |
| AIE52940 | PA | H1N1 | 2013/06/19 | (A/Santiago/p18d0/2013(H1N1)) |
| AIE52941 | PB1 | H1N1 | 2013/06/19 | (A/Santiago/p18d0/2013(H1N1)) |
| AIE52942 | PB2 | H1N1 | 2013/06/19 | (A/Santiago/p18d0/2013(H1N1)) |
| AIE52943 | HA | H3N2 | 2012/07/05 | (A/Santiago/p19d1/2012(H3N2)) |
| AIE52944 | M1 | H3N2 | 2012/07/05 | (A/Santiago/p19d1/2012(H3N2)) |
| AIE52945 | M2 | H3N2 | 2012/07/05 | (A/Santiago/p19d1/2012(H3N2)) |
| AIE52946 | NA | H3N2 | 2012/07/05 | (A/Santiago/p19d1/2012(H3N2)) |
| AIE52947 | NP | H3N2 | 2012/07/05 | (A/Santiago/p19d1/2012(H3N2)) |
| AIE52948 | NS1 | H3N2 | 2012/07/05 | (A/Santiago/p19d1/2012(H3N2)) |
| AIE52949 | NS2 | H3N2 | 2012/07/05 | (A/Santiago/p19d1/2012(H3N2)) |
| AIE52950 | PA-X | H3N2 | 2012/07/05 | (A/Santiago/p19d1/2012(H3N2)) |
| AIE52951 | PA | H3N2 | 2012/07/05 | (A/Santiago/p19d1/2012(H3N2)) |
| AIE52952 | PB1 | H3N2 | 2012/07/05 | (A/Santiago/p19d1/2012(H3N2)) |
| AIE52953 | PB2 | H3N2 | 2012/07/05 | (A/Santiago/p19d1/2012(H3N2)) |
| AIE52954 | HA | H3N2 | 2012/06/02 | (A/Santiago/op17d1/2012(H3N2)) |
| AIE52955 | M1 | H3N2 | 2012/06/02 | (A/Santiago/op17d1/2012(H3N2)) |
| AIE52956 | M2 | H3N2 | 2012/06/02 | (A/Santiago/op17d1/2012(H3N2)) |
| AIE52958 | NP | H3N2 | 2012/06/02 | (A/Santiago/op17d1/2012(H3N2)) |
| AIE52961 | PA-X | H3N2 | 2012/06/02 | (A/Santiago/op17d1/2012(H3N2)) |
| AIE52962 | PA | H3N2 | 2012/06/02 | (A/Santiago/op17d1/2012(H3N2)) |
| AIE52963 | PB1 | H3N2 | 2012/06/02 | (A/Santiago/op17d1/2012(H3N2)) |
| AIE52964 | PB2 | H3N2 | 2012/06/02 | (A/Santiago/op17d1/2012(H3N2)) |
| AIE52965 | HA | H3N2 | 2012/10/30 | (A/Santiago/p37d0/2012(H3N2)) |
| AIE52966 | M1 | H3N2 | 2012/10/30 | (A/Santiago/p37d0/2012(H3N2)) |
| AIE52967 | M2 | H3N2 | 2012/10/30 | (A/Santiago/p37d0/2012(H3N2)) |
| AIE52968 | NA | H3N2 | 2012/10/30 | (A/Santiago/p37d0/2012(H3N2)) |
| AIE52969 | NP | H3N2 | 2012/10/30 | (A/Santiago/p37d0/2012(H3N2)) |
| AIE52970 | NS1 | H3N2 | 2012/10/30 | (A/Santiago/p37d0/2012(H3N2)) |
| AIE52971 | NS2 | H3N2 | 2012/10/30 | (A/Santiago/p37d0/2012(H3N2)) |
| AIE52972 | PA-X | H3N2 | 2012/10/30 | (A/Santiago/p37d0/2012(H3N2)) |
| AIE52973 | PA | H3N2 | 2012/10/30 | (A/Santiago/p37d0/2012(H3N2)) |
| AIE52974 | PB1 | H3N2 | 2012/10/30 | (A/Santiago/p37d0/2012(H3N2)) |
| AIE52975 | PB1-F2 | H3N2 | 2012/10/30 | (A/Santiago/p37d0/2012(H3N2)) |
| AIE52976 | PB2 | H3N2 | 2012/10/30 | (A/Santiago/p37d0/2012(H3N2)) |
| AIE52977 | HA | H1N1 | 2013/07/08 | (A/Santiago/p25d0/2013(H1N1)) |
| AIE52978 | M1 | H1N1 | 2013/07/08 | (A/Santiago/p25d0/2013(H1N1)) |
| AIE52979 | M2 | H1N1 | 2013/07/08 | (A/Santiago/p25d0/2013(H1N1)) |
| AIE52980 | NA | H1N1 | 2013/07/08 | (A/Santiago/p25d0/2013(H1N1)) |
| AIE52981 | NP | H1N1 | 2013/07/08 | (A/Santiago/p25d0/2013(H1N1)) |
| AIE52982 | NS1 | H1N1 | 2013/07/08 | (A/Santiago/p25d0/2013(H1N1)) |
| AIE52983 | NS2 | H1N1 | 2013/07/08 | (A/Santiago/p25d0/2013(H1N1)) |
| AIE52984 | PA-X | H1N1 | 2013/07/08 | (A/Santiago/p25d0/2013(H1N1)) |
| AIE52985 | PA | H1N1 | 2013/07/08 | (A/Santiago/p25d0/2013(H1N1)) |
| AIE52986 | PB1 | H1N1 | 2013/07/08 | (A/Santiago/p25d0/2013(H1N1)) |
| AIE52987 | PB2 | H1N1 | 2013/07/08 | (A/Santiago/p25d0/2013(H1N1)) |
| AIE52988 | HA | H3N2 | 2012/11/01 | (A/Santiago/p37d2/2012(H3N2)) |
| AIE52989 | M1 | H3N2 | 2012/11/01 | (A/Santiago/p37d2/2012(H3N2)) |
| AIE52990 | M2 | H3N2 | 2012/11/01 | (A/Santiago/p37d2/2012(H3N2)) |
| AIE52992 | NP | H3N2 | 2012/11/01 | (A/Santiago/p37d2/2012(H3N2)) |
| AIE52993 | NS1 | H3N2 | 2012/11/01 | (A/Santiago/p37d2/2012(H3N2)) |
| AIE52995 | PA-X | H3N2 | 2012/11/01 | (A/Santiago/p37d2/2012(H3N2)) |
| AIE52996 | PA | H3N2 | 2012/11/01 | (A/Santiago/p37d2/2012(H3N2)) |
| AIE52997 | PB1 | H3N2 | 2012/11/01 | (A/Santiago/p37d2/2012(H3N2)) |
| AIE52998 | PB1-F2 | H3N2 | 2012/11/01 | (A/Santiago/p37d2/2012(H3N2)) |
| AIE52999 | PB2 | H3N2 | 2012/11/01 | (A/Santiago/p37d2/2012(H3N2)) |
| AIE53000 | HA | H3 | 2012/12/31 | (A/Santiago/p38-2012d5/2012(H3)) |
| AIE53001 | PB2 | H3 | 2012/12/31 | (A/Santiago/p38-2012d5/2012(H3)) |
| AIE53002 | HA | H3N2 | 2012/07/24 | (A/Santiago/op3d1/2012(H3N2)) |
| AIE53003 | M1 | H3N2 | 2012/07/24 | (A/Santiago/op3d1/2012(H3N2)) |
| AIE53004 | M2 | H3N2 | 2012/07/24 | (A/Santiago/op3d1/2012(H3N2)) |
| AIE53005 | NA | H3N2 | 2012/07/24 | (A/Santiago/op3d1/2012(H3N2)) |
| AIE53006 | NP | H3N2 | 2012/07/24 | (A/Santiago/op3d1/2012(H3N2)) |
| AIE53007 | NS1 | H3N2 | 2012/07/24 | (A/Santiago/op3d1/2012(H3N2)) |
| AIE53008 | NS2 | H3N2 | 2012/07/24 | (A/Santiago/op3d1/2012(H3N2)) |
| AIE53009 | PA-X | H3N2 | 2012/07/24 | (A/Santiago/op3d1/2012(H3N2)) |
| AIE53010 | PA | H3N2 | 2012/07/24 | (A/Santiago/op3d1/2012(H3N2)) |
| AIE53011 | PB1 | H3N2 | 2012/07/24 | (A/Santiago/op3d1/2012(H3N2)) |
| AIE53012 | PB2 | H3N2 | 2012/07/24 | (A/Santiago/op3d1/2012(H3N2)) |
| AIE53013 | HA | H3N2 | 2013/08/07 | (A/Santiago/p34d0/2013(H3N2)) |
| AIE53014 | M1 | H3N2 | 2013/08/07 | (A/Santiago/p34d0/2013(H3N2)) |
| AIE53015 | M2 | H3N2 | 2013/08/07 | (A/Santiago/p34d0/2013(H3N2)) |
| AIE53016 | NA | H3N2 | 2013/08/07 | (A/Santiago/p34d0/2013(H3N2)) |
| AIE53017 | NP | H3N2 | 2013/08/07 | (A/Santiago/p34d0/2013(H3N2)) |
| AIE53018 | NS1 | H3N2 | 2013/08/07 | (A/Santiago/p34d0/2013(H3N2)) |
| AIE53019 | NS2 | H3N2 | 2013/08/07 | (A/Santiago/p34d0/2013(H3N2)) |
| AIE53020 | PA-X | H3N2 | 2013/08/07 | (A/Santiago/p34d0/2013(H3N2)) |
| AIE53021 | PA | H3N2 | 2013/08/07 | (A/Santiago/p34d0/2013(H3N2)) |
| AIE53022 | PB1 | H3N2 | 2013/08/07 | (A/Santiago/p34d0/2013(H3N2)) |
| AIE53023 | PB1-F2 | H3N2 | 2013/08/07 | (A/Santiago/p34d0/2013(H3N2)) |
| AIE53024 | PB2 | H3N2 | 2013/08/07 | (A/Santiago/p34d0/2013(H3N2)) |
| AIE53025 | HA | H1N1 | 2013/06/20 | (A/Santiago/p20d0/2013(H1N1)) |
| AIE53026 | M1 | H1N1 | 2013/06/20 | (A/Santiago/p20d0/2013(H1N1)) |
| AIE53027 | M2 | H1N1 | 2013/06/20 | (A/Santiago/p20d0/2013(H1N1)) |
| AIE53028 | NA | H1N1 | 2013/06/20 | (A/Santiago/p20d0/2013(H1N1)) |
| AIE53029 | NP | H1N1 | 2013/06/20 | (A/Santiago/p20d0/2013(H1N1)) |
| AIE53030 | NS1 | H1N1 | 2013/06/20 | (A/Santiago/p20d0/2013(H1N1)) |
| AIE53031 | NS2 | H1N1 | 2013/06/20 | (A/Santiago/p20d0/2013(H1N1)) |
| AIE53032 | PA-X | H1N1 | 2013/06/20 | (A/Santiago/p20d0/2013(H1N1)) |
| AIE53033 | PA | H1N1 | 2013/06/20 | (A/Santiago/p20d0/2013(H1N1)) |
| AIE53034 | PB1 | H1N1 | 2013/06/20 | (A/Santiago/p20d0/2013(H1N1)) |
| AIE53035 | PB2 | H1N1 | 2013/06/20 | (A/Santiago/p20d0/2013(H1N1)) |
| AIE53036 | HA | H1N1 | 2013/06/20 | (A/Santiago/p18d1/2013(H1N1)) |
| AIE53037 | M1 | H1N1 | 2013/06/20 | (A/Santiago/p18d1/2013(H1N1)) |
| AIE53038 | M2 | H1N1 | 2013/06/20 | (A/Santiago/p18d1/2013(H1N1)) |
| AIE53039 | NA | H1N1 | 2013/06/20 | (A/Santiago/p18d1/2013(H1N1)) |
| AIE53040 | NP | H1N1 | 2013/06/20 | (A/Santiago/p18d1/2013(H1N1)) |
| AIE53041 | NS1 | H1N1 | 2013/06/20 | (A/Santiago/p18d1/2013(H1N1)) |
| AIE53042 | NS2 | H1N1 | 2013/06/20 | (A/Santiago/p18d1/2013(H1N1)) |
| AIE53043 | PA-X | H1N1 | 2013/06/20 | (A/Santiago/p18d1/2013(H1N1)) |
| AIE53044 | PA | H1N1 | 2013/06/20 | (A/Santiago/p18d1/2013(H1N1)) |
| AIE53045 | PB1 | H1N1 | 2013/06/20 | (A/Santiago/p18d1/2013(H1N1)) |
| AIE53046 | PB2 | H1N1 | 2013/06/20 | (A/Santiago/p18d1/2013(H1N1)) |
| AIE53047 | HA | H1N1 | 2013/06/12 | (A/Santiago/p13d1/2013(H1N1)) |
| AIE53048 | M1 | H1N1 | 2013/06/12 | (A/Santiago/p13d1/2013(H1N1)) |
| AIE53049 | M2 | H1N1 | 2013/06/12 | (A/Santiago/p13d1/2013(H1N1)) |
| AIE53051 | NP | H1N1 | 2013/06/12 | (A/Santiago/p13d1/2013(H1N1)) |
| AIE53052 | NS1 | H1N1 | 2013/06/12 | (A/Santiago/p13d1/2013(H1N1)) |
| AIE53053 | NS2 | H1N1 | 2013/06/12 | (A/Santiago/p13d1/2013(H1N1)) |
| AIE53054 | PA-X | H1N1 | 2013/06/12 | (A/Santiago/p13d1/2013(H1N1)) |
| AIE53055 | PA | H1N1 | 2013/06/12 | (A/Santiago/p13d1/2013(H1N1)) |
| AIE53056 | PB1 | H1N1 | 2013/06/12 | (A/Santiago/p13d1/2013(H1N1)) |
| AIE53057 | PB2 | H1N1 | 2013/06/12 | (A/Santiago/p13d1/2013(H1N1)) |
| AIE53058 | HA | H3N2 | 2013/07/12 | (A/Santiago/p27d0/2013(H3N2)) |
| AIE53059 | M1 | H3N2 | 2013/07/12 | (A/Santiago/p27d0/2013(H3N2)) |
| AIE53060 | M2 | H3N2 | 2013/07/12 | (A/Santiago/p27d0/2013(H3N2)) |
| AIE53061 | NA | H3N2 | 2013/07/12 | (A/Santiago/p27d0/2013(H3N2)) |
| AIE53062 | NP | H3N2 | 2013/07/12 | (A/Santiago/p27d0/2013(H3N2)) |
| AIE53063 | NS1 | H3N2 | 2013/07/12 | (A/Santiago/p27d0/2013(H3N2)) |
| AIE53064 | NS2 | H3N2 | 2013/07/12 | (A/Santiago/p27d0/2013(H3N2)) |
| AIE53065 | PA-X | H3N2 | 2013/07/12 | (A/Santiago/p27d0/2013(H3N2)) |
| AIE53066 | PA | H3N2 | 2013/07/12 | (A/Santiago/p27d0/2013(H3N2)) |
| AIE53067 | PB1 | H3N2 | 2013/07/12 | (A/Santiago/p27d0/2013(H3N2)) |
| AIE53068 | PB1-F2 | H3N2 | 2013/07/12 | (A/Santiago/p27d0/2013(H3N2)) |
| AIE53069 | PB2 | H3N2 | 2013/07/12 | (A/Santiago/p27d0/2013(H3N2)) |
| AIE53070 | HA | H3N2 | 2012/06/29 | (A/Santiago/p19d0/2012(H3N2)) |
| AIE53071 | M1 | H3N2 | 2012/06/29 | (A/Santiago/p19d0/2012(H3N2)) |
| AIE53072 | M2 | H3N2 | 2012/06/29 | (A/Santiago/p19d0/2012(H3N2)) |
| AIE53073 | NA | H3N2 | 2012/06/29 | (A/Santiago/p19d0/2012(H3N2)) |
| AIE53074 | NP | H3N2 | 2012/06/29 | (A/Santiago/p19d0/2012(H3N2)) |
| AIE53075 | NS1 | H3N2 | 2012/06/29 | (A/Santiago/p19d0/2012(H3N2)) |
| AIE53076 | NS2 | H3N2 | 2012/06/29 | (A/Santiago/p19d0/2012(H3N2)) |
| AIE53077 | PA-X | H3N2 | 2012/06/29 | (A/Santiago/p19d0/2012(H3N2)) |
| AIE53078 | PA | H3N2 | 2012/06/29 | (A/Santiago/p19d0/2012(H3N2)) |
| AIE53079 | PB1 | H3N2 | 2012/06/29 | (A/Santiago/p19d0/2012(H3N2)) |
| AIE53080 | PB2 | H3N2 | 2012/06/29 | (A/Santiago/p19d0/2012(H3N2)) |
| AIE53081 | HA | H3N2 | 2013/08/31 | (A/Santiago/p36d2/2013(H3N2)) |
| AIE53082 | M1 | H3N2 | 2013/08/31 | (A/Santiago/p36d2/2013(H3N2)) |
| AIE53083 | M2 | H3N2 | 2013/08/31 | (A/Santiago/p36d2/2013(H3N2)) |
| AIE53084 | NA | H3N2 | 2013/08/31 | (A/Santiago/p36d2/2013(H3N2)) |
| AIE53085 | NP | H3N2 | 2013/08/31 | (A/Santiago/p36d2/2013(H3N2)) |
| AIE53086 | NS1 | H3N2 | 2013/08/31 | (A/Santiago/p36d2/2013(H3N2)) |
| AIE53087 | NS2 | H3N2 | 2013/08/31 | (A/Santiago/p36d2/2013(H3N2)) |
| AIE53088 | PA-X | H3N2 | 2013/08/31 | (A/Santiago/p36d2/2013(H3N2)) |
| AIE53089 | PA | H3N2 | 2013/08/31 | (A/Santiago/p36d2/2013(H3N2)) |
| AIE53090 | PB1 | H3N2 | 2013/08/31 | (A/Santiago/p36d2/2013(H3N2)) |
| AIE53091 | PB1-F2 | H3N2 | 2013/08/31 | (A/Santiago/p36d2/2013(H3N2)) |
| AIE53092 | PB2 | H3N2 | 2013/08/31 | (A/Santiago/p36d2/2013(H3N2)) |
| AIE53093 | HA | H1N1 | 2013/06/21 | (A/Santiago/p18d2/2013(H1N1)) |
| AIE53094 | M1 | H1N1 | 2013/06/21 | (A/Santiago/p18d2/2013(H1N1)) |
| AIE53095 | M2 | H1N1 | 2013/06/21 | (A/Santiago/p18d2/2013(H1N1)) |
| AIE53096 | NA | H1N1 | 2013/06/21 | (A/Santiago/p18d2/2013(H1N1)) |
| AIE53097 | NP | H1N1 | 2013/06/21 | (A/Santiago/p18d2/2013(H1N1)) |
| AIE53098 | NS1 | H1N1 | 2013/06/21 | (A/Santiago/p18d2/2013(H1N1)) |
| AIE53099 | NS2 | H1N1 | 2013/06/21 | (A/Santiago/p18d2/2013(H1N1)) |
| AIE53100 | PA-X | H1N1 | 2013/06/21 | (A/Santiago/p18d2/2013(H1N1)) |
| AIE53101 | PA | H1N1 | 2013/06/21 | (A/Santiago/p18d2/2013(H1N1)) |
| AIE53102 | PB1 | H1N1 | 2013/06/21 | (A/Santiago/p18d2/2013(H1N1)) |
| AIE53103 | PB2 | H1N1 | 2013/06/21 | (A/Santiago/p18d2/2013(H1N1)) |
| AIE53104 | NA | N2 | 2013/06/27 | (A/Santiago/p20d7/2013(N2)) |
| AIE53105 | NP | N2 | 2013/06/27 | (A/Santiago/p20d7/2013(N2)) |
| AIE53106 | PA-X | N2 | 2013/06/27 | (A/Santiago/p20d7/2013(N2)) |
| AIE53107 | PA | N2 | 2013/06/27 | (A/Santiago/p20d7/2013(N2)) |
| AIE53108 | HA | H3N2 | 2012/12/27 | (A/Santiago/p38-2012d1/2012(H3N2)) |
| AIE53109 | M1 | H3N2 | 2012/12/27 | (A/Santiago/p38-2012d1/2012(H3N2)) |
| AIE53110 | M2 | H3N2 | 2012/12/27 | (A/Santiago/p38-2012d1/2012(H3N2)) |
| AIE53111 | NA | H3N2 | 2012/12/27 | (A/Santiago/p38-2012d1/2012(H3N2)) |
| AIE53112 | NP | H3N2 | 2012/12/27 | (A/Santiago/p38-2012d1/2012(H3N2)) |
| AIE53113 | NS1 | H3N2 | 2012/12/27 | (A/Santiago/p38-2012d1/2012(H3N2)) |
| AIE53114 | NS2 | H3N2 | 2012/12/27 | (A/Santiago/p38-2012d1/2012(H3N2)) |
| AIE53115 | PA-X | H3N2 | 2012/12/27 | (A/Santiago/p38-2012d1/2012(H3N2)) |
| AIE53116 | PA | H3N2 | 2012/12/27 | (A/Santiago/p38-2012d1/2012(H3N2)) |
| AIE53117 | PB1 | H3N2 | 2012/12/27 | (A/Santiago/p38-2012d1/2012(H3N2)) |
| AIE53118 | PB1-F2 | H3N2 | 2012/12/27 | (A/Santiago/p38-2012d1/2012(H3N2)) |
| AIE53119 | PB2 | H3N2 | 2012/12/27 | (A/Santiago/p38-2012d1/2012(H3N2)) |
| AIE53120 | HA | H3N2 | 2012/11/08 | (A/Santiago/op20d1/2012(H3N2)) |
| AIE53121 | M1 | H3N2 | 2012/11/08 | (A/Santiago/op20d1/2012(H3N2)) |
| AIE53122 | M2 | H3N2 | 2012/11/08 | (A/Santiago/op20d1/2012(H3N2)) |
| AIE53123 | NA | H3N2 | 2012/11/08 | (A/Santiago/op20d1/2012(H3N2)) |
| AIE53124 | NP | H3N2 | 2012/11/08 | (A/Santiago/op20d1/2012(H3N2)) |
| AIE53125 | NS1 | H3N2 | 2012/11/08 | (A/Santiago/op20d1/2012(H3N2)) |
| AIE53126 | NS2 | H3N2 | 2012/11/08 | (A/Santiago/op20d1/2012(H3N2)) |
| AIE53127 | PA-X | H3N2 | 2012/11/08 | (A/Santiago/op20d1/2012(H3N2)) |
| AIE53128 | PA | H3N2 | 2012/11/08 | (A/Santiago/op20d1/2012(H3N2)) |
| AIE53129 | PB1 | H3N2 | 2012/11/08 | (A/Santiago/op20d1/2012(H3N2)) |
| AIE53130 | PB1-F2 | H3N2 | 2012/11/08 | (A/Santiago/op20d1/2012(H3N2)) |
| AIE53131 | PB2 | H3N2 | 2012/11/08 | (A/Santiago/op20d1/2012(H3N2)) |
| AIE53132 | HA | H3N2 | 2012/06/23 | (A/Santiago/p5d3/2012(H3N2)) |
| AIE53133 | M1 | H3N2 | 2012/06/23 | (A/Santiago/p5d3/2012(H3N2)) |
| AIE53134 | M2 | H3N2 | 2012/06/23 | (A/Santiago/p5d3/2012(H3N2)) |
| AIE53135 | NA | H3N2 | 2012/06/23 | (A/Santiago/p5d3/2012(H3N2)) |
| AIE53136 | NP | H3N2 | 2012/06/23 | (A/Santiago/p5d3/2012(H3N2)) |
| AIE53137 | NS1 | H3N2 | 2012/06/23 | (A/Santiago/p5d3/2012(H3N2)) |
| AIE53138 | NS2 | H3N2 | 2012/06/23 | (A/Santiago/p5d3/2012(H3N2)) |
| AIE53139 | PA-X | H3N2 | 2012/06/23 | (A/Santiago/p5d3/2012(H3N2)) |
| AIE53140 | PA | H3N2 | 2012/06/23 | (A/Santiago/p5d3/2012(H3N2)) |
| AIE53141 | PB1 | H3N2 | 2012/06/23 | (A/Santiago/p5d3/2012(H3N2)) |
| AIE53142 | PB2 | H3N2 | 2012/06/23 | (A/Santiago/p5d3/2012(H3N2)) |
| AIE53143 | HA | H1N1 | 2011/08/01 | (A/Santiago/p21d1/2011(H1N1)) |
| AIE53144 | M1 | H1N1 | 2011/08/01 | (A/Santiago/p21d1/2011(H1N1)) |
| AIE53145 | M2 | H1N1 | 2011/08/01 | (A/Santiago/p21d1/2011(H1N1)) |
| AIE53146 | NA | H1N1 | 2011/08/01 | (A/Santiago/p21d1/2011(H1N1)) |
| AIE53147 | NP | H1N1 | 2011/08/01 | (A/Santiago/p21d1/2011(H1N1)) |
| AIE53148 | NS1 | H1N1 | 2011/08/01 | (A/Santiago/p21d1/2011(H1N1)) |
| AIE53149 | NS2 | H1N1 | 2011/08/01 | (A/Santiago/p21d1/2011(H1N1)) |
| AIE53150 | PA-X | H1N1 | 2011/08/01 | (A/Santiago/p21d1/2011(H1N1)) |
| AIE53151 | PA | H1N1 | 2011/08/01 | (A/Santiago/p21d1/2011(H1N1)) |
| AIE53152 | PB1 | H1N1 | 2011/08/01 | (A/Santiago/p21d1/2011(H1N1)) |
| AIE53153 | PB2 | H1N1 | 2011/08/01 | (A/Santiago/p21d1/2011(H1N1)) |
| AIE53154 | HA | H3N2 | 2013/08/14 | (A/Santiago/p34d7/2013(H3N2)) |
| AIE53155 | M1 | H3N2 | 2013/08/14 | (A/Santiago/p34d7/2013(H3N2)) |
| AIE53156 | M2 | H3N2 | 2013/08/14 | (A/Santiago/p34d7/2013(H3N2)) |
| AIE53158 | NP | H3N2 | 2013/08/14 | (A/Santiago/p34d7/2013(H3N2)) |
| AIE53159 | NS1 | H3N2 | 2013/08/14 | (A/Santiago/p34d7/2013(H3N2)) |
| AIE53160 | NS2 | H3N2 | 2013/08/14 | (A/Santiago/p34d7/2013(H3N2)) |
| AIE53161 | PA-X | H3N2 | 2013/08/14 | (A/Santiago/p34d7/2013(H3N2)) |
| AIE53162 | PA | H3N2 | 2013/08/14 | (A/Santiago/p34d7/2013(H3N2)) |
| AIE53163 | PB1 | H3N2 | 2013/08/14 | (A/Santiago/p34d7/2013(H3N2)) |
| AIE53164 | PB1-F2 | H3N2 | 2013/08/14 | (A/Santiago/p34d7/2013(H3N2)) |
| AIE53165 | PB2 | H3N2 | 2013/08/14 | (A/Santiago/p34d7/2013(H3N2)) |
| AIE53166 | HA | H3 | 2012/11/06 | (A/Santiago/p37d7/2012(H3)) |
| AIE53167 | M1 | H3 | 2012/11/06 | (A/Santiago/p37d7/2012(H3)) |
| AIE53168 | M2 | H3 | 2012/11/06 | (A/Santiago/p37d7/2012(H3)) |
| AIE53169 | M1 |  | 2013/07/19 | (A/Santiago/p27d7/2013) |
| AIE53170 | M2 |  | 2013/07/19 | (A/Santiago/p27d7/2013) |
| AIE53171 | NP |  | 2013/07/19 | (A/Santiago/p27d7/2013) |
| AIE53172 | PB1 |  | 2013/07/19 | (A/Santiago/p27d7/2013) |
| AIE53173 | PB1-F2 |  | 2013/07/19 | (A/Santiago/p27d7/2013) |
| AIE53174 | HA | H3N2 | 2012/06/20 | (A/Santiago/p5d0/2012(H3N2)) |
| AIE53175 | M1 | H3N2 | 2012/06/20 | (A/Santiago/p5d0/2012(H3N2)) |
| AIE53176 | M2 | H3N2 | 2012/06/20 | (A/Santiago/p5d0/2012(H3N2)) |
| AIE53177 | NA | H3N2 | 2012/06/20 | (A/Santiago/p5d0/2012(H3N2)) |
| AIE53178 | NP | H3N2 | 2012/06/20 | (A/Santiago/p5d0/2012(H3N2)) |
| AIE53179 | NS1 | H3N2 | 2012/06/20 | (A/Santiago/p5d0/2012(H3N2)) |
| AIE53180 | NS2 | H3N2 | 2012/06/20 | (A/Santiago/p5d0/2012(H3N2)) |
| AIE53181 | PA-X | H3N2 | 2012/06/20 | (A/Santiago/p5d0/2012(H3N2)) |
| AIE53182 | PA | H3N2 | 2012/06/20 | (A/Santiago/p5d0/2012(H3N2)) |
| AIE53183 | PB1 | H3N2 | 2012/06/20 | (A/Santiago/p5d0/2012(H3N2)) |
| AIE53184 | PB2 | H3N2 | 2012/06/20 | (A/Santiago/p5d0/2012(H3N2)) |
| AIE53185 | HA | H3N2 | 2012/06/21 | (A/Santiago/p5d1/2012(H3N2)) |
| AIE53186 | M1 | H3N2 | 2012/06/21 | (A/Santiago/p5d1/2012(H3N2)) |
| AIE53187 | M2 | H3N2 | 2012/06/21 | (A/Santiago/p5d1/2012(H3N2)) |
| AIE53188 | NA | H3N2 | 2012/06/21 | (A/Santiago/p5d1/2012(H3N2)) |
| AIE53189 | NP | H3N2 | 2012/06/21 | (A/Santiago/p5d1/2012(H3N2)) |
| AIE53190 | NS1 | H3N2 | 2012/06/21 | (A/Santiago/p5d1/2012(H3N2)) |
| AIE53191 | NS2 | H3N2 | 2012/06/21 | (A/Santiago/p5d1/2012(H3N2)) |
| AIE53192 | PA-X | H3N2 | 2012/06/21 | (A/Santiago/p5d1/2012(H3N2)) |
| AIE53193 | PA | H3N2 | 2012/06/21 | (A/Santiago/p5d1/2012(H3N2)) |
| AIE53194 | PB1 | H3N2 | 2012/06/21 | (A/Santiago/p5d1/2012(H3N2)) |
| AIE53195 | PB2 | H3N2 | 2012/06/21 | (A/Santiago/p5d1/2012(H3N2)) |
| AIE53196 | HA | H3N2 | 2012/11/02 | (A/Santiago/p37d3/2012(H3N2)) |
| AIE53197 | M1 | H3N2 | 2012/11/02 | (A/Santiago/p37d3/2012(H3N2)) |
| AIE53198 | M2 | H3N2 | 2012/11/02 | (A/Santiago/p37d3/2012(H3N2)) |
| AIE53199 | NA | H3N2 | 2012/11/02 | (A/Santiago/p37d3/2012(H3N2)) |
| AIE53200 | NP | H3N2 | 2012/11/02 | (A/Santiago/p37d3/2012(H3N2)) |
| AIE53201 | NS1 | H3N2 | 2012/11/02 | (A/Santiago/p37d3/2012(H3N2)) |
| AIE53202 | NS2 | H3N2 | 2012/11/02 | (A/Santiago/p37d3/2012(H3N2)) |
| AIE53203 | PA-X | H3N2 | 2012/11/02 | (A/Santiago/p37d3/2012(H3N2)) |
| AIE53204 | PA | H3N2 | 2012/11/02 | (A/Santiago/p37d3/2012(H3N2)) |
| AIE53205 | PB1 | H3N2 | 2012/11/02 | (A/Santiago/p37d3/2012(H3N2)) |
| AIE53206 | PB1-F2 | H3N2 | 2012/11/02 | (A/Santiago/p37d3/2012(H3N2)) |
| AIE53207 | PB2 | H3N2 | 2012/11/02 | (A/Santiago/p37d3/2012(H3N2)) |
| AIE53208 | HA | H3N2 | 2013/07/15 | (A/Santiago/p27d3/2013(H3N2)) |
| AIE53209 | M1 | H3N2 | 2013/07/15 | (A/Santiago/p27d3/2013(H3N2)) |
| AIE53210 | M2 | H3N2 | 2013/07/15 | (A/Santiago/p27d3/2013(H3N2)) |
| AIE53211 | NA | H3N2 | 2013/07/15 | (A/Santiago/p27d3/2013(H3N2)) |
| AIE53212 | NP | H3N2 | 2013/07/15 | (A/Santiago/p27d3/2013(H3N2)) |
| AIE53213 | NS1 | H3N2 | 2013/07/15 | (A/Santiago/p27d3/2013(H3N2)) |
| AIE53214 | NS2 | H3N2 | 2013/07/15 | (A/Santiago/p27d3/2013(H3N2)) |
| AIE53215 | PA-X | H3N2 | 2013/07/15 | (A/Santiago/p27d3/2013(H3N2)) |
| AIE53216 | PA | H3N2 | 2013/07/15 | (A/Santiago/p27d3/2013(H3N2)) |
| AIE53217 | PB1 | H3N2 | 2013/07/15 | (A/Santiago/p27d3/2013(H3N2)) |
| AIE53218 | PB1-F2 | H3N2 | 2013/07/15 | (A/Santiago/p27d3/2013(H3N2)) |
| AIE53219 | PB2 | H3N2 | 2013/07/15 | (A/Santiago/p27d3/2013(H3N2)) |
| AIE53220 | HA | H1N1 | 2011/08/02 | (A/Santiago/p21d2/2011(H1N1)) |
| AIE53221 | M1 | H1N1 | 2011/08/02 | (A/Santiago/p21d2/2011(H1N1)) |
| AIE53222 | M2 | H1N1 | 2011/08/02 | (A/Santiago/p21d2/2011(H1N1)) |
| AIE53223 | NA | H1N1 | 2011/08/02 | (A/Santiago/p21d2/2011(H1N1)) |
| AIE53224 | NP | H1N1 | 2011/08/02 | (A/Santiago/p21d2/2011(H1N1)) |
| AIE53225 | NS1 | H1N1 | 2011/08/02 | (A/Santiago/p21d2/2011(H1N1)) |
| AIE53226 | NS2 | H1N1 | 2011/08/02 | (A/Santiago/p21d2/2011(H1N1)) |
| AIE53227 | PA-X | H1N1 | 2011/08/02 | (A/Santiago/p21d2/2011(H1N1)) |
| AIE53228 | PA | H1N1 | 2011/08/02 | (A/Santiago/p21d2/2011(H1N1)) |
| AIE53229 | PB1 | H1N1 | 2011/08/02 | (A/Santiago/p21d2/2011(H1N1)) |
| AIE53230 | PB2 | H1N1 | 2011/08/02 | (A/Santiago/p21d2/2011(H1N1)) |
| AIE53232 | M1 | H3N2 | 2012/05/17 | (A/Santiago/op18d7/2012(H3N2)) |
| AIE53233 | M2 | H3N2 | 2012/05/17 | (A/Santiago/op18d7/2012(H3N2)) |
| AIE53236 | HA | H3N2 | 2012/06/28 | (A/Santiago/op7d1/2012(H3N2)) |
| AIE53237 | M1 | H3N2 | 2012/06/28 | (A/Santiago/op7d1/2012(H3N2)) |
| AIE53238 | M2 | H3N2 | 2012/06/28 | (A/Santiago/op7d1/2012(H3N2)) |
| AIE53239 | NA | H3N2 | 2012/06/28 | (A/Santiago/op7d1/2012(H3N2)) |
| AIE53240 | NP | H3N2 | 2012/06/28 | (A/Santiago/op7d1/2012(H3N2)) |
| AIE53241 | NS1 | H3N2 | 2012/06/28 | (A/Santiago/op7d1/2012(H3N2)) |
| AIE53242 | NS2 | H3N2 | 2012/06/28 | (A/Santiago/op7d1/2012(H3N2)) |
| AIE53243 | PA-X | H3N2 | 2012/06/28 | (A/Santiago/op7d1/2012(H3N2)) |
| AIE53244 | PA | H3N2 | 2012/06/28 | (A/Santiago/op7d1/2012(H3N2)) |
| AIE53245 | PB1 | H3N2 | 2012/06/28 | (A/Santiago/op7d1/2012(H3N2)) |
| AIE53246 | PB2 | H3N2 | 2012/06/28 | (A/Santiago/op7d1/2012(H3N2)) |
| AIE53247 | HA | H3N2 | 2013/08/08 | (A/Santiago/p34d1/2013(H3N2)) |
| AIE53248 | M1 | H3N2 | 2013/08/08 | (A/Santiago/p34d1/2013(H3N2)) |
| AIE53249 | M2 | H3N2 | 2013/08/08 | (A/Santiago/p34d1/2013(H3N2)) |
| AIE53250 | NA | H3N2 | 2013/08/08 | (A/Santiago/p34d1/2013(H3N2)) |
| AIE53251 | NP | H3N2 | 2013/08/08 | (A/Santiago/p34d1/2013(H3N2)) |
| AIE53252 | NS1 | H3N2 | 2013/08/08 | (A/Santiago/p34d1/2013(H3N2)) |
| AIE53253 | NS2 | H3N2 | 2013/08/08 | (A/Santiago/p34d1/2013(H3N2)) |
| AIE53254 | PA-X | H3N2 | 2013/08/08 | (A/Santiago/p34d1/2013(H3N2)) |
| AIE53255 | PA | H3N2 | 2013/08/08 | (A/Santiago/p34d1/2013(H3N2)) |
| AIE53256 | PB1 | H3N2 | 2013/08/08 | (A/Santiago/p34d1/2013(H3N2)) |
| AIE53257 | PB1-F2 | H3N2 | 2013/08/08 | (A/Santiago/p34d1/2013(H3N2)) |
| AIE53258 | PB2 | H3N2 | 2013/08/08 | (A/Santiago/p34d1/2013(H3N2)) |
| AIE53259 | M1 |  | 2013/03/22 | (A/Santiago/p7d1/2013) |
| AIE53260 | M2 |  | 2013/03/22 | (A/Santiago/p7d1/2013) |
| AIE53261 | HA | H1N1 | 2013/06/13 | (A/Santiago/p13d2/2013(H1N1)) |
| AIE53262 | M1 | H1N1 | 2013/06/13 | (A/Santiago/p13d2/2013(H1N1)) |
| AIE53263 | M2 | H1N1 | 2013/06/13 | (A/Santiago/p13d2/2013(H1N1)) |
| AIE53264 | NA | H1N1 | 2013/06/13 | (A/Santiago/p13d2/2013(H1N1)) |
| AIE53265 | NP | H1N1 | 2013/06/13 | (A/Santiago/p13d2/2013(H1N1)) |
| AIE53266 | NS1 | H1N1 | 2013/06/13 | (A/Santiago/p13d2/2013(H1N1)) |
| AIE53267 | NS2 | H1N1 | 2013/06/13 | (A/Santiago/p13d2/2013(H1N1)) |
| AIE53268 | PA-X | H1N1 | 2013/06/13 | (A/Santiago/p13d2/2013(H1N1)) |
| AIE53269 | PA | H1N1 | 2013/06/13 | (A/Santiago/p13d2/2013(H1N1)) |
| AIE53270 | PB1 | H1N1 | 2013/06/13 | (A/Santiago/p13d2/2013(H1N1)) |
| AIE53271 | PB2 | H1N1 | 2013/06/13 | (A/Santiago/p13d2/2013(H1N1)) |
| AIE53272 | HA | H1N1 | 2011/08/03 | (A/Santiago/p21d3/2011(H1N1)) |
| AIE53273 | M1 | H1N1 | 2011/08/03 | (A/Santiago/p21d3/2011(H1N1)) |
| AIE53274 | M2 | H1N1 | 2011/08/03 | (A/Santiago/p21d3/2011(H1N1)) |
| AIE53275 | NA | H1N1 | 2011/08/03 | (A/Santiago/p21d3/2011(H1N1)) |
| AIE53276 | NP | H1N1 | 2011/08/03 | (A/Santiago/p21d3/2011(H1N1)) |
| AIE53277 | NS1 | H1N1 | 2011/08/03 | (A/Santiago/p21d3/2011(H1N1)) |
| AIE53278 | NS2 | H1N1 | 2011/08/03 | (A/Santiago/p21d3/2011(H1N1)) |
| AIE53279 | PA-X | H1N1 | 2011/08/03 | (A/Santiago/p21d3/2011(H1N1)) |
| AIE53280 | PA | H1N1 | 2011/08/03 | (A/Santiago/p21d3/2011(H1N1)) |
| AIE53281 | PB1 | H1N1 | 2011/08/03 | (A/Santiago/p21d3/2011(H1N1)) |
| AIE53282 | PB2 | H1N1 | 2011/08/03 | (A/Santiago/p21d3/2011(H1N1)) |
| AIE53283 | HA | H3N2 | 2013/09/02 | (A/Santiago/p36d5/2013(H3N2)) |
| AIE53284 | M1 | H3N2 | 2013/09/02 | (A/Santiago/p36d5/2013(H3N2)) |
| AIE53285 | M2 | H3N2 | 2013/09/02 | (A/Santiago/p36d5/2013(H3N2)) |
| AIE53286 | NA | H3N2 | 2013/09/02 | (A/Santiago/p36d5/2013(H3N2)) |
| AIE53287 | NP | H3N2 | 2013/09/02 | (A/Santiago/p36d5/2013(H3N2)) |
| AIE53288 | NS1 | H3N2 | 2013/09/02 | (A/Santiago/p36d5/2013(H3N2)) |
| AIE53289 | NS2 | H3N2 | 2013/09/02 | (A/Santiago/p36d5/2013(H3N2)) |
| AIE53290 | PA-X | H3N2 | 2013/09/02 | (A/Santiago/p36d5/2013(H3N2)) |
| AIE53291 | PA | H3N2 | 2013/09/02 | (A/Santiago/p36d5/2013(H3N2)) |
| AIE53292 | PB1 | H3N2 | 2013/09/02 | (A/Santiago/p36d5/2013(H3N2)) |
| AIE53293 | PB1-F2 | H3N2 | 2013/09/02 | (A/Santiago/p36d5/2013(H3N2)) |
| AIE53294 | PB2 | H3N2 | 2013/09/02 | (A/Santiago/p36d5/2013(H3N2)) |
| AIE53295 | HA | H1N1 | 2013/06/22 | (A/Santiago/p20d2/2013(H1N1)) |
| AIE53296 | M1 | H1N1 | 2013/06/22 | (A/Santiago/p20d2/2013(H1N1)) |
| AIE53297 | M2 | H1N1 | 2013/06/22 | (A/Santiago/p20d2/2013(H1N1)) |
| AIE53298 | NA | H1N1 | 2013/06/22 | (A/Santiago/p20d2/2013(H1N1)) |
| AIE53299 | NP | H1N1 | 2013/06/22 | (A/Santiago/p20d2/2013(H1N1)) |
| AIE53300 | NS1 | H1N1 | 2013/06/22 | (A/Santiago/p20d2/2013(H1N1)) |
| AIE53301 | NS2 | H1N1 | 2013/06/22 | (A/Santiago/p20d2/2013(H1N1)) |
| AIE53302 | PA-X | H1N1 | 2013/06/22 | (A/Santiago/p20d2/2013(H1N1)) |
| AIE53303 | PA | H1N1 | 2013/06/22 | (A/Santiago/p20d2/2013(H1N1)) |
| AIE53304 | PB1 | H1N1 | 2013/06/22 | (A/Santiago/p20d2/2013(H1N1)) |
| AIE53305 | PB2 | H1N1 | 2013/06/22 | (A/Santiago/p20d2/2013(H1N1)) |
| AIE53306 | HA | H1N1 | 2013/06/21 | (A/Santiago/p20d1/2013(H1N1)) |
| AIE53307 | M1 | H1N1 | 2013/06/21 | (A/Santiago/p20d1/2013(H1N1)) |
| AIE53308 | M2 | H1N1 | 2013/06/21 | (A/Santiago/p20d1/2013(H1N1)) |
| AIE53309 | NA | H1N1 | 2013/06/21 | (A/Santiago/p20d1/2013(H1N1)) |
| AIE53310 | NP | H1N1 | 2013/06/21 | (A/Santiago/p20d1/2013(H1N1)) |
| AIE53311 | NS1 | H1N1 | 2013/06/21 | (A/Santiago/p20d1/2013(H1N1)) |
| AIE53312 | NS2 | H1N1 | 2013/06/21 | (A/Santiago/p20d1/2013(H1N1)) |
| AIE53313 | PA-X | H1N1 | 2013/06/21 | (A/Santiago/p20d1/2013(H1N1)) |
| AIE53314 | PA | H1N1 | 2013/06/21 | (A/Santiago/p20d1/2013(H1N1)) |
| AIE53315 | PB1 | H1N1 | 2013/06/21 | (A/Santiago/p20d1/2013(H1N1)) |
| AIE53316 | PB2 | H1N1 | 2013/06/21 | (A/Santiago/p20d1/2013(H1N1)) |
| AIE53317 | HA | H3N2 | 2012/12/29 | (A/Santiago/p38-2012d3/2012(H3N2)) |
| AIE53318 | M1 | H3N2 | 2012/12/29 | (A/Santiago/p38-2012d3/2012(H3N2)) |
| AIE53319 | M2 | H3N2 | 2012/12/29 | (A/Santiago/p38-2012d3/2012(H3N2)) |
| AIE53320 | NA | H3N2 | 2012/12/29 | (A/Santiago/p38-2012d3/2012(H3N2)) |
| AIE53321 | NP | H3N2 | 2012/12/29 | (A/Santiago/p38-2012d3/2012(H3N2)) |
| AIE53322 | NS1 | H3N2 | 2012/12/29 | (A/Santiago/p38-2012d3/2012(H3N2)) |
| AIE53323 | NS2 | H3N2 | 2012/12/29 | (A/Santiago/p38-2012d3/2012(H3N2)) |
| AIE53324 | PA-X | H3N2 | 2012/12/29 | (A/Santiago/p38-2012d3/2012(H3N2)) |
| AIE53325 | PA | H3N2 | 2012/12/29 | (A/Santiago/p38-2012d3/2012(H3N2)) |
| AIE53326 | PB1 | H3N2 | 2012/12/29 | (A/Santiago/p38-2012d3/2012(H3N2)) |
| AIE53327 | PB1-F2 | H3N2 | 2012/12/29 | (A/Santiago/p38-2012d3/2012(H3N2)) |
| AIE53328 | PB2 | H3N2 | 2012/12/29 | (A/Santiago/p38-2012d3/2012(H3N2)) |
| AIE53329 | HA | H3N2 | 2012/06/01 | (A/Santiago/op17d0/2012(H3N2)) |
| AIE53330 | M1 | H3N2 | 2012/06/01 | (A/Santiago/op17d0/2012(H3N2)) |
| AIE53331 | M2 | H3N2 | 2012/06/01 | (A/Santiago/op17d0/2012(H3N2)) |
| AIE53332 | NA | H3N2 | 2012/06/01 | (A/Santiago/op17d0/2012(H3N2)) |
| AIE53333 | NP | H3N2 | 2012/06/01 | (A/Santiago/op17d0/2012(H3N2)) |
| AIE53334 | NS1 | H3N2 | 2012/06/01 | (A/Santiago/op17d0/2012(H3N2)) |
| AIE53335 | NS2 | H3N2 | 2012/06/01 | (A/Santiago/op17d0/2012(H3N2)) |
| AIE53336 | PA-X | H3N2 | 2012/06/01 | (A/Santiago/op17d0/2012(H3N2)) |
| AIE53337 | PA | H3N2 | 2012/06/01 | (A/Santiago/op17d0/2012(H3N2)) |
| AIE53338 | PB1 | H3N2 | 2012/06/01 | (A/Santiago/op17d0/2012(H3N2)) |
| AIE53339 | PB2 | H3N2 | 2012/06/01 | (A/Santiago/op17d0/2012(H3N2)) |
| AIE53341 | M1 | H1N1 | 2011/08/03 | (A/Santiago/p20d3/2011(H1N1)) |
| AIE53342 | M2 | H1N1 | 2011/08/03 | (A/Santiago/p20d3/2011(H1N1)) |
| AIE53343 | NA | H1N1 | 2011/08/03 | (A/Santiago/p20d3/2011(H1N1)) |
| AIE53344 | NP | H1N1 | 2011/08/03 | (A/Santiago/p20d3/2011(H1N1)) |
| AIE53345 | NS1 | H1N1 | 2011/08/03 | (A/Santiago/p20d3/2011(H1N1)) |
| AIE53346 | NS2 | H1N1 | 2011/08/03 | (A/Santiago/p20d3/2011(H1N1)) |
| AIE53347 | PA-X | H1N1 | 2011/08/03 | (A/Santiago/p20d3/2011(H1N1)) |
| AIE53348 | PA | H1N1 | 2011/08/03 | (A/Santiago/p20d3/2011(H1N1)) |
| AIE53349 | PB1 | H1N1 | 2011/08/03 | (A/Santiago/p20d3/2011(H1N1)) |
| AIE53350 | PB2 | H1N1 | 2011/08/03 | (A/Santiago/p20d3/2011(H1N1)) |
| AIE53351 | HA | H1N1 | 2011/08/02 | (A/Santiago/p20d2/2011(H1N1)) |
| AIE53352 | M1 | H1N1 | 2011/08/02 | (A/Santiago/p20d2/2011(H1N1)) |
| AIE53353 | M2 | H1N1 | 2011/08/02 | (A/Santiago/p20d2/2011(H1N1)) |
| AIE53354 | NA | H1N1 | 2011/08/02 | (A/Santiago/p20d2/2011(H1N1)) |
| AIE53355 | NP | H1N1 | 2011/08/02 | (A/Santiago/p20d2/2011(H1N1)) |
| AIE53356 | NS1 | H1N1 | 2011/08/02 | (A/Santiago/p20d2/2011(H1N1)) |
| AIE53357 | NS2 | H1N1 | 2011/08/02 | (A/Santiago/p20d2/2011(H1N1)) |
| AIE53358 | PA-X | H1N1 | 2011/08/02 | (A/Santiago/p20d2/2011(H1N1)) |
| AIE53359 | PA | H1N1 | 2011/08/02 | (A/Santiago/p20d2/2011(H1N1)) |
| AIE53360 | PB1 | H1N1 | 2011/08/02 | (A/Santiago/p20d2/2011(H1N1)) |
| AIE53361 | PB2 | H1N1 | 2011/08/02 | (A/Santiago/p20d2/2011(H1N1)) |
| AIE53364 | NA | H1N1 | 2011/08/18 | (A/Santiago/p3d3/2011(H1N1)) |
| AIE53365 | NP | H1N1 | 2011/08/18 | (A/Santiago/p3d3/2011(H1N1)) |
| AIE53366 | NS1 | H1N1 | 2011/08/18 | (A/Santiago/p3d3/2011(H1N1)) |
| AIE53367 | NS2 | H1N1 | 2011/08/18 | (A/Santiago/p3d3/2011(H1N1)) |
| AIE53368 | HA | H1N1 | 2011/07/12 | (A/Santiago/p1d1/2011(H1N1)) |
| AIE53369 | M1 | H1N1 | 2011/07/12 | (A/Santiago/p1d1/2011(H1N1)) |
| AIE53370 | M2 | H1N1 | 2011/07/12 | (A/Santiago/p1d1/2011(H1N1)) |
| AIE53371 | NA | H1N1 | 2011/07/12 | (A/Santiago/p1d1/2011(H1N1)) |
| AIE53372 | NP | H1N1 | 2011/07/12 | (A/Santiago/p1d1/2011(H1N1)) |
| AIE53373 | NS1 | H1N1 | 2011/07/12 | (A/Santiago/p1d1/2011(H1N1)) |
| AIE53374 | NS2 | H1N1 | 2011/07/12 | (A/Santiago/p1d1/2011(H1N1)) |
| AIE53375 | PA-X | H1N1 | 2011/07/12 | (A/Santiago/p1d1/2011(H1N1)) |
| AIE53376 | PA | H1N1 | 2011/07/12 | (A/Santiago/p1d1/2011(H1N1)) |
| AIE53377 | PB1 | H1N1 | 2011/07/12 | (A/Santiago/p1d1/2011(H1N1)) |
| AIE53378 | PB2 | H1N1 | 2011/07/12 | (A/Santiago/p1d1/2011(H1N1)) |
| AIE53379 | M1 | N1 | 2011/08/22 | (A/Santiago/p12d3/2011(N1)) |
| AIE53380 | M2 | N1 | 2011/08/22 | (A/Santiago/p12d3/2011(N1)) |
| AIE53383 | NS1 | N1 | 2011/08/22 | (A/Santiago/p12d3/2011(N1)) |
| AIE53384 | NS2 | N1 | 2011/08/22 | (A/Santiago/p12d3/2011(N1)) |
| AIE53385 | PB2 | N1 | 2011/08/22 | (A/Santiago/p12d3/2011(N1)) |
| AIE53386 | M1 | H1N1 | 2011/08/04 | (A/Santiago/p19d5/2011(H1N1)) |
| AIE53387 | M2 | H1N1 | 2011/08/04 | (A/Santiago/p19d5/2011(H1N1)) |
| AIE53389 | NP | H1N1 | 2011/08/04 | (A/Santiago/p19d5/2011(H1N1)) |
| AIE53390 | NS1 | H1N1 | 2011/08/04 | (A/Santiago/p19d5/2011(H1N1)) |
| AIE53391 | NS2 | H1N1 | 2011/08/04 | (A/Santiago/p19d5/2011(H1N1)) |
| AIE53394 | NS1 | H1 | 2011/08/02 | (A/Santiago/p18d1/2011(H1)) |
| AIE53395 | NS2 | H1 | 2011/08/02 | (A/Santiago/p18d1/2011(H1)) |
| AIE53396 | M1 |  | 2011/08/03 | (A/Santiago/p2d3/2011) |
| AIE53397 | M2 |  | 2011/08/03 | (A/Santiago/p2d3/2011) |
| AIE53398 | NS1 |  | 2011/08/03 | (A/Santiago/p2d3/2011) |
| AIE53399 | NS2 |  | 2011/08/03 | (A/Santiago/p2d3/2011) |
| AIE53400 | HA | H1N1 | 2011/08/22 | (A/Santiago/p9d1/2011(H1N1)) |
| AIE53401 | M1 | H1N1 | 2011/08/22 | (A/Santiago/p9d1/2011(H1N1)) |
| AIE53402 | M2 | H1N1 | 2011/08/22 | (A/Santiago/p9d1/2011(H1N1)) |
| AIE53403 | NA | H1N1 | 2011/08/22 | (A/Santiago/p9d1/2011(H1N1)) |
| AIE53404 | NP | H1N1 | 2011/08/22 | (A/Santiago/p9d1/2011(H1N1)) |
| AIE53405 | NS1 | H1N1 | 2011/08/22 | (A/Santiago/p9d1/2011(H1N1)) |
| AIE53406 | NS2 | H1N1 | 2011/08/22 | (A/Santiago/p9d1/2011(H1N1)) |
| AIE53407 | PA-X | H1N1 | 2011/08/22 | (A/Santiago/p9d1/2011(H1N1)) |
| AIE53408 | PA | H1N1 | 2011/08/22 | (A/Santiago/p9d1/2011(H1N1)) |
| AIE53409 | PB1 | H1N1 | 2011/08/22 | (A/Santiago/p9d1/2011(H1N1)) |
| AIE53410 | PB2 | H1N1 | 2011/08/22 | (A/Santiago/p9d1/2011(H1N1)) |
| AIE53411 | HA | H1N1 | 2011/08/25 | (A/Santiago/p9d3/2011(H1N1)) |
| AIE53412 | M1 | H1N1 | 2011/08/25 | (A/Santiago/p9d3/2011(H1N1)) |
| AIE53413 | M2 | H1N1 | 2011/08/25 | (A/Santiago/p9d3/2011(H1N1)) |
| AIE53414 | NA | H1N1 | 2011/08/25 | (A/Santiago/p9d3/2011(H1N1)) |
| AIE53415 | NP | H1N1 | 2011/08/25 | (A/Santiago/p9d3/2011(H1N1)) |
| AIE53416 | NS1 | H1N1 | 2011/08/25 | (A/Santiago/p9d3/2011(H1N1)) |
| AIE53417 | NS2 | H1N1 | 2011/08/25 | (A/Santiago/p9d3/2011(H1N1)) |
| AIE53418 | PA-X | H1N1 | 2011/08/25 | (A/Santiago/p9d3/2011(H1N1)) |
| AIE53419 | PA | H1N1 | 2011/08/25 | (A/Santiago/p9d3/2011(H1N1)) |
| AIE53420 | PB1 | H1N1 | 2011/08/25 | (A/Santiago/p9d3/2011(H1N1)) |
| AIE53421 | PB2 | H1N1 | 2011/08/25 | (A/Santiago/p9d3/2011(H1N1)) |
| AIE53422 | HA | H1N1 | 2011/07/31 | (A/Santiago/p19d1/2011(H1N1)) |
| AIE53423 | M1 | H1N1 | 2011/07/31 | (A/Santiago/p19d1/2011(H1N1)) |
| AIE53424 | M2 | H1N1 | 2011/07/31 | (A/Santiago/p19d1/2011(H1N1)) |
| AIE53425 | NA | H1N1 | 2011/07/31 | (A/Santiago/p19d1/2011(H1N1)) |
| AIE53426 | NP | H1N1 | 2011/07/31 | (A/Santiago/p19d1/2011(H1N1)) |
| AIE53427 | NS1 | H1N1 | 2011/07/31 | (A/Santiago/p19d1/2011(H1N1)) |
| AIE53428 | NS2 | H1N1 | 2011/07/31 | (A/Santiago/p19d1/2011(H1N1)) |
| AIE53429 | PA-X | H1N1 | 2011/07/31 | (A/Santiago/p19d1/2011(H1N1)) |
| AIE53430 | PA | H1N1 | 2011/07/31 | (A/Santiago/p19d1/2011(H1N1)) |
| AIE53431 | PB1 | H1N1 | 2011/07/31 | (A/Santiago/p19d1/2011(H1N1)) |
| AIE53432 | PB2 | H1N1 | 2011/07/31 | (A/Santiago/p19d1/2011(H1N1)) |
| AIE53435 | NS1 | H1N1 | 2011/08/29 | (A/Santiago/p10d1/2011(H1N1)) |
| AIE53436 | NS2 | H1N1 | 2011/08/29 | (A/Santiago/p10d1/2011(H1N1)) |
| AIE53437 | M1 | H1N1 | 2011/08/17 | (A/Santiago/p15d2/2011(H1N1)) |
| AIE53438 | M2 | H1N1 | 2011/08/17 | (A/Santiago/p15d2/2011(H1N1)) |
| AIE53440 | PB2 | H1N1 | 2011/08/17 | (A/Santiago/p15d2/2011(H1N1)) |
| AIE53441 | M1 | N1 | 2011/08/08 | (A/Santiago/p22d1/2011(N1)) |
| AIE53442 | M2 | N1 | 2011/08/08 | (A/Santiago/p22d1/2011(N1)) |
| AIE53443 | NA | N1 | 2011/08/08 | (A/Santiago/p22d1/2011(N1)) |
| AIE53444 | NS1 | N1 | 2011/08/08 | (A/Santiago/p22d1/2011(N1)) |
| AIE53445 | NS2 | N1 | 2011/08/08 | (A/Santiago/p22d1/2011(N1)) |
| AIE53446 | HA | H1 | 2011/08/05 | (A/Santiago/p2d5/2011(H1)) |
| AIE53447 | M1 | H1 | 2011/08/05 | (A/Santiago/p2d5/2011(H1)) |
| AIE53448 | M2 | H1 | 2011/08/05 | (A/Santiago/p2d5/2011(H1)) |
| AIE53449 | PB2 | H1 | 2011/08/05 | (A/Santiago/p2d5/2011(H1)) |
| AIE53450 | HA | H1N1 | 2011/08/22 | (A/Santiago/p13d1/2011(H1N1)) |
| AIE53451 | M1 | H1N1 | 2011/08/22 | (A/Santiago/p13d1/2011(H1N1)) |
| AIE53452 | M2 | H1N1 | 2011/08/22 | (A/Santiago/p13d1/2011(H1N1)) |
| AIE53453 | NA | H1N1 | 2011/08/22 | (A/Santiago/p13d1/2011(H1N1)) |
| AIE53454 | NP | H1N1 | 2011/08/22 | (A/Santiago/p13d1/2011(H1N1)) |
| AIE53455 | NS1 | H1N1 | 2011/08/22 | (A/Santiago/p13d1/2011(H1N1)) |
| AIE53456 | NS2 | H1N1 | 2011/08/22 | (A/Santiago/p13d1/2011(H1N1)) |
| AIE53457 | PA-X | H1N1 | 2011/08/22 | (A/Santiago/p13d1/2011(H1N1)) |
| AIE53458 | PA | H1N1 | 2011/08/22 | (A/Santiago/p13d1/2011(H1N1)) |
| AIE53459 | PB1 | H1N1 | 2011/08/22 | (A/Santiago/p13d1/2011(H1N1)) |
| AIE53460 | PB2 | H1N1 | 2011/08/22 | (A/Santiago/p13d1/2011(H1N1)) |
| AIE53461 | HA | H1N1 | 2011/08/27 | (A/Santiago/p9d5/2011(H1N1)) |
| AIE53462 | M1 | H1N1 | 2011/08/27 | (A/Santiago/p9d5/2011(H1N1)) |
| AIE53463 | M2 | H1N1 | 2011/08/27 | (A/Santiago/p9d5/2011(H1N1)) |
| AIE53464 | NA | H1N1 | 2011/08/27 | (A/Santiago/p9d5/2011(H1N1)) |
| AIE53465 | NP | H1N1 | 2011/08/27 | (A/Santiago/p9d5/2011(H1N1)) |
| AIE53466 | NS1 | H1N1 | 2011/08/27 | (A/Santiago/p9d5/2011(H1N1)) |
| AIE53467 | NS2 | H1N1 | 2011/08/27 | (A/Santiago/p9d5/2011(H1N1)) |
| AIE53468 | PA-X | H1N1 | 2011/08/27 | (A/Santiago/p9d5/2011(H1N1)) |
| AIE53469 | PA | H1N1 | 2011/08/27 | (A/Santiago/p9d5/2011(H1N1)) |
| AIE53470 | PB1 | H1N1 | 2011/08/27 | (A/Santiago/p9d5/2011(H1N1)) |
| AIE53471 | PB2 | H1N1 | 2011/08/27 | (A/Santiago/p9d5/2011(H1N1)) |
| AIE53472 | M1 | H1N1 | 2011/08/02 | (A/Santiago/p19d3/2011(H1N1)) |
| AIE53473 | M2 | H1N1 | 2011/08/02 | (A/Santiago/p19d3/2011(H1N1)) |
| AIE53474 | NA | H1N1 | 2011/08/02 | (A/Santiago/p19d3/2011(H1N1)) |
| AIE53475 | NP | H1N1 | 2011/08/02 | (A/Santiago/p19d3/2011(H1N1)) |
| AIE53476 | NS1 | H1N1 | 2011/08/02 | (A/Santiago/p19d3/2011(H1N1)) |
| AIE53477 | NS2 | H1N1 | 2011/08/02 | (A/Santiago/p19d3/2011(H1N1)) |
| AIE53478 | HA | H1N1 | 2011/08/23 | (A/Santiago/p13d2/2011(H1N1)) |
| AIE53479 | M1 | H1N1 | 2011/08/23 | (A/Santiago/p13d2/2011(H1N1)) |
| AIE53480 | M2 | H1N1 | 2011/08/23 | (A/Santiago/p13d2/2011(H1N1)) |
| AIE53481 | NA | H1N1 | 2011/08/23 | (A/Santiago/p13d2/2011(H1N1)) |
| AIE53482 | NP | H1N1 | 2011/08/23 | (A/Santiago/p13d2/2011(H1N1)) |
| AIE53483 | NS1 | H1N1 | 2011/08/23 | (A/Santiago/p13d2/2011(H1N1)) |
| AIE53484 | NS2 | H1N1 | 2011/08/23 | (A/Santiago/p13d2/2011(H1N1)) |
| AIE53485 | PA-X | H1N1 | 2011/08/23 | (A/Santiago/p13d2/2011(H1N1)) |
| AIE53486 | PA | H1N1 | 2011/08/23 | (A/Santiago/p13d2/2011(H1N1)) |
| AIE53487 | PB1 | H1N1 | 2011/08/23 | (A/Santiago/p13d2/2011(H1N1)) |
| AIE53488 | PB2 | H1N1 | 2011/08/23 | (A/Santiago/p13d2/2011(H1N1)) |
| AIE53489 | HA | H1N1 | 2011/08/16 | (A/Santiago/p15d1/2011(H1N1)) |
| AIE53490 | M1 | H1N1 | 2011/08/16 | (A/Santiago/p15d1/2011(H1N1)) |
| AIE53491 | M2 | H1N1 | 2011/08/16 | (A/Santiago/p15d1/2011(H1N1)) |
| AIE53492 | NA | H1N1 | 2011/08/16 | (A/Santiago/p15d1/2011(H1N1)) |
| AIE53493 | NP | H1N1 | 2011/08/16 | (A/Santiago/p15d1/2011(H1N1)) |
| AIE53494 | NS1 | H1N1 | 2011/08/16 | (A/Santiago/p15d1/2011(H1N1)) |
| AIE53495 | NS2 | H1N1 | 2011/08/16 | (A/Santiago/p15d1/2011(H1N1)) |
| AIE53496 | PA-X | H1N1 | 2011/08/16 | (A/Santiago/p15d1/2011(H1N1)) |
| AIE53497 | PA | H1N1 | 2011/08/16 | (A/Santiago/p15d1/2011(H1N1)) |
| AIE53498 | PB1 | H1N1 | 2011/08/16 | (A/Santiago/p15d1/2011(H1N1)) |
| AIE53499 | PB2 | H1N1 | 2011/08/16 | (A/Santiago/p15d1/2011(H1N1)) |
| AIE53501 | M1 | H1N1 | 2011/08/22 | (A/Santiago/p3d7/2011(H1N1)) |
| AIE53502 | M2 | H1N1 | 2011/08/22 | (A/Santiago/p3d7/2011(H1N1)) |
| AIE53508 | M1 | H1N1 | 2011/08/26 | (A/Santiago/p13d5/2011(H1N1)) |
| AIE53509 | M2 | H1N1 | 2011/08/26 | (A/Santiago/p13d5/2011(H1N1)) |
| AIE53510 | NA | H1N1 | 2011/08/26 | (A/Santiago/p13d5/2011(H1N1)) |
| AIE53511 | NP | H1N1 | 2011/08/26 | (A/Santiago/p13d5/2011(H1N1)) |
| AIE53512 | NS1 | H1N1 | 2011/08/26 | (A/Santiago/p13d5/2011(H1N1)) |
| AIE53513 | NS2 | H1N1 | 2011/08/26 | (A/Santiago/p13d5/2011(H1N1)) |
| AIE53514 | PA-X | H1N1 | 2011/08/26 | (A/Santiago/p13d5/2011(H1N1)) |
| AIE53515 | PA | H1N1 | 2011/08/26 | (A/Santiago/p13d5/2011(H1N1)) |
| AIE53516 | PB2 | H1N1 | 2011/08/26 | (A/Santiago/p13d5/2011(H1N1)) |
| AIE53517 | NS1 | N1 | 2011/08/06 | (A/Santiago/p19d7/2011(N1)) |
| AIE53518 | NS2 | N1 | 2011/08/06 | (A/Santiago/p19d7/2011(N1)) |
| AIE53519 | HA | H1N1 | 2011/08/20 | (A/Santiago/p12d1/2011(H1N1)) |
| AIE53520 | M1 | H1N1 | 2011/08/20 | (A/Santiago/p12d1/2011(H1N1)) |
| AIE53521 | M2 | H1N1 | 2011/08/20 | (A/Santiago/p12d1/2011(H1N1)) |
| AIE53522 | NA | H1N1 | 2011/08/20 | (A/Santiago/p12d1/2011(H1N1)) |
| AIE53523 | NP | H1N1 | 2011/08/20 | (A/Santiago/p12d1/2011(H1N1)) |
| AIE53524 | NS1 | H1N1 | 2011/08/20 | (A/Santiago/p12d1/2011(H1N1)) |
| AIE53525 | NS2 | H1N1 | 2011/08/20 | (A/Santiago/p12d1/2011(H1N1)) |
| AIE53526 | PB1 | H1N1 | 2011/08/20 | (A/Santiago/p12d1/2011(H1N1)) |
| AIE53527 | PB2 | H1N1 | 2011/08/20 | (A/Santiago/p12d1/2011(H1N1)) |
| AIE53528 | PB1 |  | 2011/08/18 | (A/Santiago/p15d3/2011) |
| AIE53529 | HA | H1N1 | 2011/08/05 | (A/Santiago/p20d5/2011(H1N1)) |
| AIE53530 | M1 | H1N1 | 2011/08/05 | (A/Santiago/p20d5/2011(H1N1)) |
| AIE53531 | M2 | H1N1 | 2011/08/05 | (A/Santiago/p20d5/2011(H1N1)) |
| AIE53532 | NA | H1N1 | 2011/08/05 | (A/Santiago/p20d5/2011(H1N1)) |
| AIE53533 | NP | H1N1 | 2011/08/05 | (A/Santiago/p20d5/2011(H1N1)) |
| AIE53534 | NS1 | H1N1 | 2011/08/05 | (A/Santiago/p20d5/2011(H1N1)) |
| AIE53535 | NS2 | H1N1 | 2011/08/05 | (A/Santiago/p20d5/2011(H1N1)) |
| AIE53536 | PA-X | H1N1 | 2011/08/05 | (A/Santiago/p20d5/2011(H1N1)) |
| AIE53537 | PA | H1N1 | 2011/08/05 | (A/Santiago/p20d5/2011(H1N1)) |
| AIE53538 | PB1 | H1N1 | 2011/08/05 | (A/Santiago/p20d5/2011(H1N1)) |
| AIE53539 | PB2 | H1N1 | 2011/08/05 | (A/Santiago/p20d5/2011(H1N1)) |
| AIE53540 | M1 | N1 | 2011/08/23 | (A/Santiago/p12d4/2011(N1)) |
| AIE53541 | M2 | N1 | 2011/08/23 | (A/Santiago/p12d4/2011(N1)) |
| AIE53543 | NS1 | N1 | 2011/08/23 | (A/Santiago/p12d4/2011(N1)) |
| AIE53544 | NS2 | N1 | 2011/08/23 | (A/Santiago/p12d4/2011(N1)) |
| AIE53547 | HA | H3N2 | 2012/07/21 | (A/Santiago/p24d2/2012(H3N2)) |
| AIE53548 | M1 | H3N2 | 2012/07/21 | (A/Santiago/p24d2/2012(H3N2)) |
| AIE53549 | M2 | H3N2 | 2012/07/21 | (A/Santiago/p24d2/2012(H3N2)) |
| AIE53550 | NA | H3N2 | 2012/07/21 | (A/Santiago/p24d2/2012(H3N2)) |
| AIE53551 | NP | H3N2 | 2012/07/21 | (A/Santiago/p24d2/2012(H3N2)) |
| AIE53552 | NS1 | H3N2 | 2012/07/21 | (A/Santiago/p24d2/2012(H3N2)) |
| AIE53553 | NS2 | H3N2 | 2012/07/21 | (A/Santiago/p24d2/2012(H3N2)) |
| AIE53554 | PA-X | H3N2 | 2012/07/21 | (A/Santiago/p24d2/2012(H3N2)) |
| AIE53555 | PA | H3N2 | 2012/07/21 | (A/Santiago/p24d2/2012(H3N2)) |
| AIE53556 | PB1 | H3N2 | 2012/07/21 | (A/Santiago/p24d2/2012(H3N2)) |
| AIE53557 | PB2 | H3N2 | 2012/07/21 | (A/Santiago/p24d2/2012(H3N2)) |
| AIE53558 | HA | H3N2 | 2012/06/14 | (A/Santiago/p2d2/2012(H3N2)) |
| AIE53559 | M1 | H3N2 | 2012/06/14 | (A/Santiago/p2d2/2012(H3N2)) |
| AIE53560 | M2 | H3N2 | 2012/06/14 | (A/Santiago/p2d2/2012(H3N2)) |
| AIE53561 | NA | H3N2 | 2012/06/14 | (A/Santiago/p2d2/2012(H3N2)) |
| AIE53562 | NP | H3N2 | 2012/06/14 | (A/Santiago/p2d2/2012(H3N2)) |
| AIE53563 | NS1 | H3N2 | 2012/06/14 | (A/Santiago/p2d2/2012(H3N2)) |
| AIE53564 | NS2 | H3N2 | 2012/06/14 | (A/Santiago/p2d2/2012(H3N2)) |
| AIE53565 | PA-X | H3N2 | 2012/06/14 | (A/Santiago/p2d2/2012(H3N2)) |
| AIE53566 | PA | H3N2 | 2012/06/14 | (A/Santiago/p2d2/2012(H3N2)) |
| AIE53567 | PB1 | H3N2 | 2012/06/14 | (A/Santiago/p2d2/2012(H3N2)) |
| AIE53568 | PB1-F2 | H3N2 | 2012/06/14 | (A/Santiago/p2d2/2012(H3N2)) |
| AIE53569 | PB2 | H3N2 | 2012/06/14 | (A/Santiago/p2d2/2012(H3N2)) |
| AIE53570 | HA | H3N2 | 2012/06/14 | (A/Santiago/p3d0/2012(H3N2)) |
| AIE53571 | M1 | H3N2 | 2012/06/14 | (A/Santiago/p3d0/2012(H3N2)) |
| AIE53572 | M2 | H3N2 | 2012/06/14 | (A/Santiago/p3d0/2012(H3N2)) |
| AIE53573 | NA | H3N2 | 2012/06/14 | (A/Santiago/p3d0/2012(H3N2)) |
| AIE53574 | NP | H3N2 | 2012/06/14 | (A/Santiago/p3d0/2012(H3N2)) |
| AIE53575 | NS1 | H3N2 | 2012/06/14 | (A/Santiago/p3d0/2012(H3N2)) |
| AIE53576 | NS2 | H3N2 | 2012/06/14 | (A/Santiago/p3d0/2012(H3N2)) |
| AIE53577 | PA-X | H3N2 | 2012/06/14 | (A/Santiago/p3d0/2012(H3N2)) |
| AIE53578 | PA | H3N2 | 2012/06/14 | (A/Santiago/p3d0/2012(H3N2)) |
| AIE53579 | PB1 | H3N2 | 2012/06/14 | (A/Santiago/p3d0/2012(H3N2)) |
| AIE53580 | PB1-F2 | H3N2 | 2012/06/14 | (A/Santiago/p3d0/2012(H3N2)) |
| AIE53581 | PB2 | H3N2 | 2012/06/14 | (A/Santiago/p3d0/2012(H3N2)) |
| AIE53582 | HA | H3N2 | 2012/06/25 | (A/Santiago/p9d3/2012(H3N2)) |
| AIE53583 | M1 | H3N2 | 2012/06/25 | (A/Santiago/p9d3/2012(H3N2)) |
| AIE53584 | M2 | H3N2 | 2012/06/25 | (A/Santiago/p9d3/2012(H3N2)) |
| AIE53585 | NA | H3N2 | 2012/06/25 | (A/Santiago/p9d3/2012(H3N2)) |
| AIE53586 | NP | H3N2 | 2012/06/25 | (A/Santiago/p9d3/2012(H3N2)) |
| AIE53587 | NS1 | H3N2 | 2012/06/25 | (A/Santiago/p9d3/2012(H3N2)) |
| AIE53588 | NS2 | H3N2 | 2012/06/25 | (A/Santiago/p9d3/2012(H3N2)) |
| AIE53589 | PA-X | H3N2 | 2012/06/25 | (A/Santiago/p9d3/2012(H3N2)) |
| AIE53590 | PA | H3N2 | 2012/06/25 | (A/Santiago/p9d3/2012(H3N2)) |
| AIE53591 | PB1 | H3N2 | 2012/06/25 | (A/Santiago/p9d3/2012(H3N2)) |
| AIE53592 | PB2 | H3N2 | 2012/06/25 | (A/Santiago/p9d3/2012(H3N2)) |
| AIE53593 | HA | H3N2 | 2012/06/23 | (A/Santiago/p9d1/2012(H3N2)) |
| AIE53594 | M1 | H3N2 | 2012/06/23 | (A/Santiago/p9d1/2012(H3N2)) |
| AIE53595 | M2 | H3N2 | 2012/06/23 | (A/Santiago/p9d1/2012(H3N2)) |
| AIE53596 | NA | H3N2 | 2012/06/23 | (A/Santiago/p9d1/2012(H3N2)) |
| AIE53597 | NP | H3N2 | 2012/06/23 | (A/Santiago/p9d1/2012(H3N2)) |
| AIE53598 | NS1 | H3N2 | 2012/06/23 | (A/Santiago/p9d1/2012(H3N2)) |
| AIE53599 | NS2 | H3N2 | 2012/06/23 | (A/Santiago/p9d1/2012(H3N2)) |
| AIE53600 | PA-X | H3N2 | 2012/06/23 | (A/Santiago/p9d1/2012(H3N2)) |
| AIE53601 | PA | H3N2 | 2012/06/23 | (A/Santiago/p9d1/2012(H3N2)) |
| AIE53602 | PB1 | H3N2 | 2012/06/23 | (A/Santiago/p9d1/2012(H3N2)) |
| AIE53603 | PB2 | H3N2 | 2012/06/23 | (A/Santiago/p9d1/2012(H3N2)) |
| AIE53604 | HA | H3N2 | 2012/06/21 | (A/Santiago/p6d1/2012(H3N2)) |
| AIE53605 | M1 | H3N2 | 2012/06/21 | (A/Santiago/p6d1/2012(H3N2)) |
| AIE53606 | M2 | H3N2 | 2012/06/21 | (A/Santiago/p6d1/2012(H3N2)) |
| AIE53607 | NA | H3N2 | 2012/06/21 | (A/Santiago/p6d1/2012(H3N2)) |
| AIE53608 | NP | H3N2 | 2012/06/21 | (A/Santiago/p6d1/2012(H3N2)) |
| AIE53609 | NS1 | H3N2 | 2012/06/21 | (A/Santiago/p6d1/2012(H3N2)) |
| AIE53610 | NS2 | H3N2 | 2012/06/21 | (A/Santiago/p6d1/2012(H3N2)) |
| AIE53611 | PA-X | H3N2 | 2012/06/21 | (A/Santiago/p6d1/2012(H3N2)) |
| AIE53612 | PA | H3N2 | 2012/06/21 | (A/Santiago/p6d1/2012(H3N2)) |
| AIE53613 | PB1 | H3N2 | 2012/06/21 | (A/Santiago/p6d1/2012(H3N2)) |
| AIE53614 | PB2 | H3N2 | 2012/06/21 | (A/Santiago/p6d1/2012(H3N2)) |
| AIE53615 | HA | H3 | 2012/06/22 | (A/Santiago/p4d5/2012(H3)) |
| AIE53616 | M1 | H3 | 2012/06/22 | (A/Santiago/p4d5/2012(H3)) |
| AIE53617 | M2 | H3 | 2012/06/22 | (A/Santiago/p4d5/2012(H3)) |
| AIE53618 | PB1 | H3 | 2012/06/22 | (A/Santiago/p4d5/2012(H3)) |
| AIE53619 | PB2 | H3 | 2012/06/22 | (A/Santiago/p4d5/2012(H3)) |
| AIE53620 | HA | H3N2 | 2012/06/06 | (A/Santiago/p1d2/2012(H3N2)) |
| AIE53621 | M1 | H3N2 | 2012/06/06 | (A/Santiago/p1d2/2012(H3N2)) |
| AIE53622 | M2 | H3N2 | 2012/06/06 | (A/Santiago/p1d2/2012(H3N2)) |
| AIE53623 | NA | H3N2 | 2012/06/06 | (A/Santiago/p1d2/2012(H3N2)) |
| AIE53624 | NP | H3N2 | 2012/06/06 | (A/Santiago/p1d2/2012(H3N2)) |
| AIE53625 | NS1 | H3N2 | 2012/06/06 | (A/Santiago/p1d2/2012(H3N2)) |
| AIE53626 | NS2 | H3N2 | 2012/06/06 | (A/Santiago/p1d2/2012(H3N2)) |
| AIE53627 | PA-X | H3N2 | 2012/06/06 | (A/Santiago/p1d2/2012(H3N2)) |
| AIE53628 | PA | H3N2 | 2012/06/06 | (A/Santiago/p1d2/2012(H3N2)) |
| AIE53629 | PB1 | H3N2 | 2012/06/06 | (A/Santiago/p1d2/2012(H3N2)) |
| AIE53630 | PB2 | H3N2 | 2012/06/06 | (A/Santiago/p1d2/2012(H3N2)) |
| AIE53631 | NS1 |  | 2012/07/13 | (A/Santiago/p9d21/2012) |
| AIE53632 | NS2 |  | 2012/07/13 | (A/Santiago/p9d21/2012) |
| AIE53633 | HA | H3N2 | 2012/07/20 | (A/Santiago/p24d1/2012(H3N2)) |
| AIE53634 | M1 | H3N2 | 2012/07/20 | (A/Santiago/p24d1/2012(H3N2)) |
| AIE53635 | M2 | H3N2 | 2012/07/20 | (A/Santiago/p24d1/2012(H3N2)) |
| AIE53636 | NA | H3N2 | 2012/07/20 | (A/Santiago/p24d1/2012(H3N2)) |
| AIE53637 | NP | H3N2 | 2012/07/20 | (A/Santiago/p24d1/2012(H3N2)) |
| AIE53638 | NS1 | H3N2 | 2012/07/20 | (A/Santiago/p24d1/2012(H3N2)) |
| AIE53639 | NS2 | H3N2 | 2012/07/20 | (A/Santiago/p24d1/2012(H3N2)) |
| AIE53640 | PA-X | H3N2 | 2012/07/20 | (A/Santiago/p24d1/2012(H3N2)) |
| AIE53641 | PA | H3N2 | 2012/07/20 | (A/Santiago/p24d1/2012(H3N2)) |
| AIE53642 | PB1 | H3N2 | 2012/07/20 | (A/Santiago/p24d1/2012(H3N2)) |
| AIE53643 | PB2 | H3N2 | 2012/07/20 | (A/Santiago/p24d1/2012(H3N2)) |
| AIE53644 | HA | H3N2 | 2012/07/05 | (A/Santiago/p18d00/2012(H3N2)) |
| AIE53645 | M1 | H3N2 | 2012/07/05 | (A/Santiago/p18d00/2012(H3N2)) |
| AIE53646 | M2 | H3N2 | 2012/07/05 | (A/Santiago/p18d00/2012(H3N2)) |
| AIE53647 | NA | H3N2 | 2012/07/05 | (A/Santiago/p18d00/2012(H3N2)) |
| AIE53648 | NP | H3N2 | 2012/07/05 | (A/Santiago/p18d00/2012(H3N2)) |
| AIE53649 | NS1 | H3N2 | 2012/07/05 | (A/Santiago/p18d00/2012(H3N2)) |
| AIE53650 | NS2 | H3N2 | 2012/07/05 | (A/Santiago/p18d00/2012(H3N2)) |
| AIE53651 | PA-X | H3N2 | 2012/07/05 | (A/Santiago/p18d00/2012(H3N2)) |
| AIE53652 | PA | H3N2 | 2012/07/05 | (A/Santiago/p18d00/2012(H3N2)) |
| AIE53653 | PB1 | H3N2 | 2012/07/05 | (A/Santiago/p18d00/2012(H3N2)) |
| AIE53654 | PB1-F2 | H3N2 | 2012/07/05 | (A/Santiago/p18d00/2012(H3N2)) |
| AIE53655 | PB2 | H3N2 | 2012/07/05 | (A/Santiago/p18d00/2012(H3N2)) |
| AIE53656 | HA | H3N2 | 2012/06/20 | (A/Santiago/p4d3/2012(H3N2)) |
| AIE53657 | M1 | H3N2 | 2012/06/20 | (A/Santiago/p4d3/2012(H3N2)) |
| AIE53658 | M2 | H3N2 | 2012/06/20 | (A/Santiago/p4d3/2012(H3N2)) |
| AIE53659 | NA | H3N2 | 2012/06/20 | (A/Santiago/p4d3/2012(H3N2)) |
| AIE53660 | NP | H3N2 | 2012/06/20 | (A/Santiago/p4d3/2012(H3N2)) |
| AIE53661 | NS1 | H3N2 | 2012/06/20 | (A/Santiago/p4d3/2012(H3N2)) |
| AIE53662 | NS2 | H3N2 | 2012/06/20 | (A/Santiago/p4d3/2012(H3N2)) |
| AIE53663 | PA-X | H3N2 | 2012/06/20 | (A/Santiago/p4d3/2012(H3N2)) |
| AIE53664 | PA | H3N2 | 2012/06/20 | (A/Santiago/p4d3/2012(H3N2)) |
| AIE53665 | PB1 | H3N2 | 2012/06/20 | (A/Santiago/p4d3/2012(H3N2)) |
| AIE53666 | PB2 | H3N2 | 2012/06/20 | (A/Santiago/p4d3/2012(H3N2)) |
| AIE53667 | HA | H3N2 | 2012/06/22 | (A/Santiago/p9d0/2012(H3N2)) |
| AIE53668 | M1 | H3N2 | 2012/06/22 | (A/Santiago/p9d0/2012(H3N2)) |
| AIE53669 | M2 | H3N2 | 2012/06/22 | (A/Santiago/p9d0/2012(H3N2)) |
| AIE53670 | NA | H3N2 | 2012/06/22 | (A/Santiago/p9d0/2012(H3N2)) |
| AIE53671 | NP | H3N2 | 2012/06/22 | (A/Santiago/p9d0/2012(H3N2)) |
| AIE53672 | NS1 | H3N2 | 2012/06/22 | (A/Santiago/p9d0/2012(H3N2)) |
| AIE53673 | NS2 | H3N2 | 2012/06/22 | (A/Santiago/p9d0/2012(H3N2)) |
| AIE53674 | PA-X | H3N2 | 2012/06/22 | (A/Santiago/p9d0/2012(H3N2)) |
| AIE53675 | PA | H3N2 | 2012/06/22 | (A/Santiago/p9d0/2012(H3N2)) |
| AIE53676 | PB1 | H3N2 | 2012/06/22 | (A/Santiago/p9d0/2012(H3N2)) |
| AIE53677 | PB2 | H3N2 | 2012/06/22 | (A/Santiago/p9d0/2012(H3N2)) |
| AIE53678 | HA | H3N2 | 2012/07/24 | (A/Santiago/p24d5/2012(H3N2)) |
| AIE53679 | M1 | H3N2 | 2012/07/24 | (A/Santiago/p24d5/2012(H3N2)) |
| AIE53680 | M2 | H3N2 | 2012/07/24 | (A/Santiago/p24d5/2012(H3N2)) |
| AIE53682 | NP | H3N2 | 2012/07/24 | (A/Santiago/p24d5/2012(H3N2)) |
| AIE53683 | NS1 | H3N2 | 2012/07/24 | (A/Santiago/p24d5/2012(H3N2)) |
| AIE53684 | NS2 | H3N2 | 2012/07/24 | (A/Santiago/p24d5/2012(H3N2)) |
| AIE53685 | PA-X | H3N2 | 2012/07/24 | (A/Santiago/p24d5/2012(H3N2)) |
| AIE53686 | PA | H3N2 | 2012/07/24 | (A/Santiago/p24d5/2012(H3N2)) |
| AIE53687 | PB1 | H3N2 | 2012/07/24 | (A/Santiago/p24d5/2012(H3N2)) |
| AIE53688 | PB1-F2 | H3N2 | 2012/07/24 | (A/Santiago/p24d5/2012(H3N2)) |
| AIE53689 | PB2 | H3N2 | 2012/07/24 | (A/Santiago/p24d5/2012(H3N2)) |
| AIE53690 | HA | H3N2 | 2012/06/16 | (A/Santiago/p3d2/2012(H3N2)) |
| AIE53694 | NP | H3N2 | 2012/06/16 | (A/Santiago/p3d2/2012(H3N2)) |
| AIE53695 | NS1 | H3N2 | 2012/06/16 | (A/Santiago/p3d2/2012(H3N2)) |
| AIE53696 | NS2 | H3N2 | 2012/06/16 | (A/Santiago/p3d2/2012(H3N2)) |
| AIE53697 | PA-X | H3N2 | 2012/06/16 | (A/Santiago/p3d2/2012(H3N2)) |
| AIE53698 | PA | H3N2 | 2012/06/16 | (A/Santiago/p3d2/2012(H3N2)) |
| AIE53699 | HA | H3N2 | 2012/06/24 | (A/Santiago/p9d2/2012(H3N2)) |
| AIE53700 | M1 | H3N2 | 2012/06/24 | (A/Santiago/p9d2/2012(H3N2)) |
| AIE53701 | M2 | H3N2 | 2012/06/24 | (A/Santiago/p9d2/2012(H3N2)) |
| AIE53702 | NA | H3N2 | 2012/06/24 | (A/Santiago/p9d2/2012(H3N2)) |
| AIE53703 | NP | H3N2 | 2012/06/24 | (A/Santiago/p9d2/2012(H3N2)) |
| AIE53704 | NS1 | H3N2 | 2012/06/24 | (A/Santiago/p9d2/2012(H3N2)) |
| AIE53705 | NS2 | H3N2 | 2012/06/24 | (A/Santiago/p9d2/2012(H3N2)) |
| AIE53706 | PA-X | H3N2 | 2012/06/24 | (A/Santiago/p9d2/2012(H3N2)) |
| AIE53707 | PA | H3N2 | 2012/06/24 | (A/Santiago/p9d2/2012(H3N2)) |
| AIE53708 | PB1 | H3N2 | 2012/06/24 | (A/Santiago/p9d2/2012(H3N2)) |
| AIE53709 | PB2 | H3N2 | 2012/06/24 | (A/Santiago/p9d2/2012(H3N2)) |
| AIE53710 | HA | H3N2 | 2012/07/26 | (A/Santiago/p25d7/2012(H3N2)) |
| AIE53711 | M1 | H3N2 | 2012/07/26 | (A/Santiago/p25d7/2012(H3N2)) |
| AIE53712 | M2 | H3N2 | 2012/07/26 | (A/Santiago/p25d7/2012(H3N2)) |
| AIE53713 | NA | H3N2 | 2012/07/26 | (A/Santiago/p25d7/2012(H3N2)) |
| AIE53714 | NP | H3N2 | 2012/07/26 | (A/Santiago/p25d7/2012(H3N2)) |
| AIE53715 | NS1 | H3N2 | 2012/07/26 | (A/Santiago/p25d7/2012(H3N2)) |
| AIE53716 | NS2 | H3N2 | 2012/07/26 | (A/Santiago/p25d7/2012(H3N2)) |
| AIE53717 | PA-X | H3N2 | 2012/07/26 | (A/Santiago/p25d7/2012(H3N2)) |
| AIE53718 | PA | H3N2 | 2012/07/26 | (A/Santiago/p25d7/2012(H3N2)) |
| AIE53719 | HA | H3N2 | 2012/07/07 | (A/Santiago/p20d0/2012(H3N2)) |
| AIE53720 | M1 | H3N2 | 2012/07/07 | (A/Santiago/p20d0/2012(H3N2)) |
| AIE53721 | M2 | H3N2 | 2012/07/07 | (A/Santiago/p20d0/2012(H3N2)) |
| AIE53722 | NA | H3N2 | 2012/07/07 | (A/Santiago/p20d0/2012(H3N2)) |
| AIE53723 | NP | H3N2 | 2012/07/07 | (A/Santiago/p20d0/2012(H3N2)) |
| AIE53724 | NS1 | H3N2 | 2012/07/07 | (A/Santiago/p20d0/2012(H3N2)) |
| AIE53725 | NS2 | H3N2 | 2012/07/07 | (A/Santiago/p20d0/2012(H3N2)) |
| AIE53726 | PA-X | H3N2 | 2012/07/07 | (A/Santiago/p20d0/2012(H3N2)) |
| AIE53727 | PA | H3N2 | 2012/07/07 | (A/Santiago/p20d0/2012(H3N2)) |
| AIE53728 | PB1 | H3N2 | 2012/07/07 | (A/Santiago/p20d0/2012(H3N2)) |
| AIE53729 | PB2 | H3N2 | 2012/07/07 | (A/Santiago/p20d0/2012(H3N2)) |
| AIE53730 | HA | H3N2 | 2012/06/05 | (A/Santiago/p1d1/2012(H3N2)) |
| AIE53731 | M1 | H3N2 | 2012/06/05 | (A/Santiago/p1d1/2012(H3N2)) |
| AIE53732 | M2 | H3N2 | 2012/06/05 | (A/Santiago/p1d1/2012(H3N2)) |
| AIE53733 | NA | H3N2 | 2012/06/05 | (A/Santiago/p1d1/2012(H3N2)) |
| AIE53734 | NP | H3N2 | 2012/06/05 | (A/Santiago/p1d1/2012(H3N2)) |
| AIE53735 | NS1 | H3N2 | 2012/06/05 | (A/Santiago/p1d1/2012(H3N2)) |
| AIE53736 | NS2 | H3N2 | 2012/06/05 | (A/Santiago/p1d1/2012(H3N2)) |
| AIE53737 | PA-X | H3N2 | 2012/06/05 | (A/Santiago/p1d1/2012(H3N2)) |
| AIE53738 | PA | H3N2 | 2012/06/05 | (A/Santiago/p1d1/2012(H3N2)) |
| AIE53739 | PB1 | H3N2 | 2012/06/05 | (A/Santiago/p1d1/2012(H3N2)) |
| AIE53740 | PB2 | H3N2 | 2012/06/05 | (A/Santiago/p1d1/2012(H3N2)) |
| AIE53742 | M1 | H3N2 | 2012/07/24 | (A/Santiago/p25d5/2012(H3N2)) |
| AIE53743 | M2 | H3N2 | 2012/07/24 | (A/Santiago/p25d5/2012(H3N2)) |
| AIE53745 | NS1 | H3N2 | 2012/07/24 | (A/Santiago/p25d5/2012(H3N2)) |
| AIE53746 | NS2 | H3N2 | 2012/07/24 | (A/Santiago/p25d5/2012(H3N2)) |
| AIE53747 | PB2 | H3N2 | 2012/07/24 | (A/Santiago/p25d5/2012(H3N2)) |
| AIE53748 | HA | H3N2 | 2012/06/26 | (A/Santiago/p9d5/2012(H3N2)) |
| AIE53749 | M1 | H3N2 | 2012/06/26 | (A/Santiago/p9d5/2012(H3N2)) |
| AIE53750 | M2 | H3N2 | 2012/06/26 | (A/Santiago/p9d5/2012(H3N2)) |
| AIE53751 | NA | H3N2 | 2012/06/26 | (A/Santiago/p9d5/2012(H3N2)) |
| AIE53752 | NP | H3N2 | 2012/06/26 | (A/Santiago/p9d5/2012(H3N2)) |
| AIE53753 | NS1 | H3N2 | 2012/06/26 | (A/Santiago/p9d5/2012(H3N2)) |
| AIE53754 | NS2 | H3N2 | 2012/06/26 | (A/Santiago/p9d5/2012(H3N2)) |
| AIE53755 | PA-X | H3N2 | 2012/06/26 | (A/Santiago/p9d5/2012(H3N2)) |
| AIE53756 | PA | H3N2 | 2012/06/26 | (A/Santiago/p9d5/2012(H3N2)) |
| AIE53757 | PB1 | H3N2 | 2012/06/26 | (A/Santiago/p9d5/2012(H3N2)) |
| AIE53758 | PB2 | H3N2 | 2012/06/26 | (A/Santiago/p9d5/2012(H3N2)) |
| AIE53759 | M1 | N2 | 2012/06/29 | (A/Santiago/p9d7/2012(N2)) |
| AIE53760 | M2 | N2 | 2012/06/29 | (A/Santiago/p9d7/2012(N2)) |
| AIE53761 | NA | N2 | 2012/06/29 | (A/Santiago/p9d7/2012(N2)) |
| AIE53762 | NS1 | N2 | 2012/06/29 | (A/Santiago/p9d7/2012(N2)) |
| AIE53764 | PB2 | N2 | 2012/06/29 | (A/Santiago/p9d7/2012(N2)) |
| AIE53765 | NA | N2 | 2012/06/19 | (A/Santiago/p3d5/2012(N2)) |
| AIE53766 | HA | H3N2 | 2012/06/19 | (A/Santiago/p4d2/2012(H3N2)) |
| AIE53767 | M1 | H3N2 | 2012/06/19 | (A/Santiago/p4d2/2012(H3N2)) |
| AIE53768 | M2 | H3N2 | 2012/06/19 | (A/Santiago/p4d2/2012(H3N2)) |
| AIE53769 | NA | H3N2 | 2012/06/19 | (A/Santiago/p4d2/2012(H3N2)) |
| AIE53770 | NP | H3N2 | 2012/06/19 | (A/Santiago/p4d2/2012(H3N2)) |
| AIE53771 | NS1 | H3N2 | 2012/06/19 | (A/Santiago/p4d2/2012(H3N2)) |
| AIE53772 | NS2 | H3N2 | 2012/06/19 | (A/Santiago/p4d2/2012(H3N2)) |
| AIE53773 | PA-X | H3N2 | 2012/06/19 | (A/Santiago/p4d2/2012(H3N2)) |
| AIE53774 | PA | H3N2 | 2012/06/19 | (A/Santiago/p4d2/2012(H3N2)) |
| AIE53775 | PB1 | H3N2 | 2012/06/19 | (A/Santiago/p4d2/2012(H3N2)) |
| AIE53776 | PB2 | H3N2 | 2012/06/19 | (A/Santiago/p4d2/2012(H3N2)) |
| AIE53777 | HA | H3N2 | 2012/07/06 | (A/Santiago/p18d0/2012(H3N2)) |
| AIE53778 | M1 | H3N2 | 2012/07/06 | (A/Santiago/p18d0/2012(H3N2)) |
| AIE53779 | M2 | H3N2 | 2012/07/06 | (A/Santiago/p18d0/2012(H3N2)) |
| AIE53780 | NA | H3N2 | 2012/07/06 | (A/Santiago/p18d0/2012(H3N2)) |
| AIE53781 | NP | H3N2 | 2012/07/06 | (A/Santiago/p18d0/2012(H3N2)) |
| AIE53782 | NS1 | H3N2 | 2012/07/06 | (A/Santiago/p18d0/2012(H3N2)) |
| AIE53783 | NS2 | H3N2 | 2012/07/06 | (A/Santiago/p18d0/2012(H3N2)) |
| AIE53784 | PA-X | H3N2 | 2012/07/06 | (A/Santiago/p18d0/2012(H3N2)) |
| AIE53785 | PA | H3N2 | 2012/07/06 | (A/Santiago/p18d0/2012(H3N2)) |
| AIE53786 | PB1 | H3N2 | 2012/07/06 | (A/Santiago/p18d0/2012(H3N2)) |
| AIE53787 | PB1-F2 | H3N2 | 2012/07/06 | (A/Santiago/p18d0/2012(H3N2)) |
| AIE53788 | PB2 | H3N2 | 2012/07/06 | (A/Santiago/p18d0/2012(H3N2)) |
| AIE53789 | NP |  | 2012/06/21 | (A/Santiago/p3d7/2012) |
| AIE53790 | HA | H3N2 | 2012/06/18 | (A/Santiago/p4d1/2012(H3N2)) |
| AIE53791 | M1 | H3N2 | 2012/06/18 | (A/Santiago/p4d1/2012(H3N2)) |
| AIE53792 | M2 | H3N2 | 2012/06/18 | (A/Santiago/p4d1/2012(H3N2)) |
| AIE53793 | NA | H3N2 | 2012/06/18 | (A/Santiago/p4d1/2012(H3N2)) |
| AIE53794 | NP | H3N2 | 2012/06/18 | (A/Santiago/p4d1/2012(H3N2)) |
| AIE53795 | NS1 | H3N2 | 2012/06/18 | (A/Santiago/p4d1/2012(H3N2)) |
| AIE53796 | NS2 | H3N2 | 2012/06/18 | (A/Santiago/p4d1/2012(H3N2)) |
| AIE53797 | PA-X | H3N2 | 2012/06/18 | (A/Santiago/p4d1/2012(H3N2)) |
| AIE53798 | PA | H3N2 | 2012/06/18 | (A/Santiago/p4d1/2012(H3N2)) |
| AIE53799 | PB1 | H3N2 | 2012/06/18 | (A/Santiago/p4d1/2012(H3N2)) |
| AIE53800 | PB2 | H3N2 | 2012/06/18 | (A/Santiago/p4d1/2012(H3N2)) |
| AIE53801 | HA | H3N2 | 2012/06/22 | (A/Santiago/p6d2/2012(H3N2)) |
| AIE53802 | M1 | H3N2 | 2012/06/22 | (A/Santiago/p6d2/2012(H3N2)) |
| AIE53803 | M2 | H3N2 | 2012/06/22 | (A/Santiago/p6d2/2012(H3N2)) |
| AIE53804 | NA | H3N2 | 2012/06/22 | (A/Santiago/p6d2/2012(H3N2)) |
| AIE53805 | NP | H3N2 | 2012/06/22 | (A/Santiago/p6d2/2012(H3N2)) |
| AIE53806 | NS1 | H3N2 | 2012/06/22 | (A/Santiago/p6d2/2012(H3N2)) |
| AIE53807 | NS2 | H3N2 | 2012/06/22 | (A/Santiago/p6d2/2012(H3N2)) |
| AIE53808 | PA-X | H3N2 | 2012/06/22 | (A/Santiago/p6d2/2012(H3N2)) |
| AIE53809 | PA | H3N2 | 2012/06/22 | (A/Santiago/p6d2/2012(H3N2)) |
| AIE53810 | PB1 | H3N2 | 2012/06/22 | (A/Santiago/p6d2/2012(H3N2)) |
| AIE53811 | PB2 | H3N2 | 2012/06/22 | (A/Santiago/p6d2/2012(H3N2)) |
| AIE53812 | M1 | N2 | 2012/06/19 | (A/Santiago/p2d7/2012(N2)) |
| AIE53813 | M2 | N2 | 2012/06/19 | (A/Santiago/p2d7/2012(N2)) |
| AIE53814 | NA | N2 | 2012/06/19 | (A/Santiago/p2d7/2012(N2)) |
| AIE53815 | NP | N2 | 2012/06/19 | (A/Santiago/p2d7/2012(N2)) |
| AIE53816 | NS1 | N2 | 2012/06/19 | (A/Santiago/p2d7/2012(N2)) |
| AIE53817 | NS2 | N2 | 2012/06/19 | (A/Santiago/p2d7/2012(N2)) |
| AIE53818 | PA-X | N2 | 2012/06/19 | (A/Santiago/p2d7/2012(N2)) |
| AIE53819 | PA | N2 | 2012/06/19 | (A/Santiago/p2d7/2012(N2)) |
| AIE53821 | HA | H3N2 | 2012/07/21 | (A/Santiago/p25d2/2012(H3N2)) |
| AIE53822 | M1 | H3N2 | 2012/07/21 | (A/Santiago/p25d2/2012(H3N2)) |
| AIE53823 | M2 | H3N2 | 2012/07/21 | (A/Santiago/p25d2/2012(H3N2)) |
| AIE53824 | NA | H3N2 | 2012/07/21 | (A/Santiago/p25d2/2012(H3N2)) |
| AIE53825 | NP | H3N2 | 2012/07/21 | (A/Santiago/p25d2/2012(H3N2)) |
| AIE53826 | NS1 | H3N2 | 2012/07/21 | (A/Santiago/p25d2/2012(H3N2)) |
| AIE53827 | NS2 | H3N2 | 2012/07/21 | (A/Santiago/p25d2/2012(H3N2)) |
| AIE53828 | PA-X | H3N2 | 2012/07/21 | (A/Santiago/p25d2/2012(H3N2)) |
| AIE53829 | PA | H3N2 | 2012/07/21 | (A/Santiago/p25d2/2012(H3N2)) |
| AIE53830 | PB1 | H3N2 | 2012/07/21 | (A/Santiago/p25d2/2012(H3N2)) |
| AIE53831 | PB1-F2 | H3N2 | 2012/07/21 | (A/Santiago/p25d2/2012(H3N2)) |
| AIE53832 | PB2 | H3N2 | 2012/07/21 | (A/Santiago/p25d2/2012(H3N2)) |
| AIE53833 | HA | H3N2 | 2012/06/23 | (A/Santiago/p6d3/2012(H3N2)) |
| AIE53834 | M1 | H3N2 | 2012/06/23 | (A/Santiago/p6d3/2012(H3N2)) |
| AIE53835 | M2 | H3N2 | 2012/06/23 | (A/Santiago/p6d3/2012(H3N2)) |
| AIE53836 | NA | H3N2 | 2012/06/23 | (A/Santiago/p6d3/2012(H3N2)) |
| AIE53837 | NP | H3N2 | 2012/06/23 | (A/Santiago/p6d3/2012(H3N2)) |
| AIE53838 | NS1 | H3N2 | 2012/06/23 | (A/Santiago/p6d3/2012(H3N2)) |
| AIE53839 | NS2 | H3N2 | 2012/06/23 | (A/Santiago/p6d3/2012(H3N2)) |
| AIE53840 | PA-X | H3N2 | 2012/06/23 | (A/Santiago/p6d3/2012(H3N2)) |
| AIE53841 | PA | H3N2 | 2012/06/23 | (A/Santiago/p6d3/2012(H3N2)) |
| AIE53842 | PB1 | H3N2 | 2012/06/23 | (A/Santiago/p6d3/2012(H3N2)) |
| AIE53843 | PB2 | H3N2 | 2012/06/23 | (A/Santiago/p6d3/2012(H3N2)) |
| AIE53844 | HA | H3N2 | 2012/06/13 | (A/Santiago/p2d1/2012(H3N2)) |
| AIE53845 | M1 | H3N2 | 2012/06/13 | (A/Santiago/p2d1/2012(H3N2)) |
| AIE53846 | M2 | H3N2 | 2012/06/13 | (A/Santiago/p2d1/2012(H3N2)) |
| AIE53847 | NA | H3N2 | 2012/06/13 | (A/Santiago/p2d1/2012(H3N2)) |
| AIE53848 | NP | H3N2 | 2012/06/13 | (A/Santiago/p2d1/2012(H3N2)) |
| AIE53849 | NS1 | H3N2 | 2012/06/13 | (A/Santiago/p2d1/2012(H3N2)) |
| AIE53850 | NS2 | H3N2 | 2012/06/13 | (A/Santiago/p2d1/2012(H3N2)) |
| AIE53851 | PA-X | H3N2 | 2012/06/13 | (A/Santiago/p2d1/2012(H3N2)) |
| AIE53852 | PA | H3N2 | 2012/06/13 | (A/Santiago/p2d1/2012(H3N2)) |
| AIE53853 | PB1 | H3N2 | 2012/06/13 | (A/Santiago/p2d1/2012(H3N2)) |
| AIE53854 | PB1-F2 | H3N2 | 2012/06/13 | (A/Santiago/p2d1/2012(H3N2)) |
| AIE53855 | PB2 | H3N2 | 2012/06/13 | (A/Santiago/p2d1/2012(H3N2)) |
| AIE53856 | HA | H3N2 | 2012/06/25 | (A/Santiago/p6d5/2012(H3N2)) |
| AIE53857 | M1 | H3N2 | 2012/06/25 | (A/Santiago/p6d5/2012(H3N2)) |
| AIE53858 | M2 | H3N2 | 2012/06/25 | (A/Santiago/p6d5/2012(H3N2)) |
| AIE53859 | NA | H3N2 | 2012/06/25 | (A/Santiago/p6d5/2012(H3N2)) |
| AIE53860 | NP | H3N2 | 2012/06/25 | (A/Santiago/p6d5/2012(H3N2)) |
| AIE53861 | NS1 | H3N2 | 2012/06/25 | (A/Santiago/p6d5/2012(H3N2)) |
| AIE53862 | NS2 | H3N2 | 2012/06/25 | (A/Santiago/p6d5/2012(H3N2)) |
| AIE53863 | PA-X | H3N2 | 2012/06/25 | (A/Santiago/p6d5/2012(H3N2)) |
| AIE53864 | PA | H3N2 | 2012/06/25 | (A/Santiago/p6d5/2012(H3N2)) |
| AIE53865 | PB1 | H3N2 | 2012/06/25 | (A/Santiago/p6d5/2012(H3N2)) |
| AIE53866 | PB2 | H3N2 | 2012/06/25 | (A/Santiago/p6d5/2012(H3N2)) |
| AIE53867 | HA | H3N2 | 2012/06/16 | (A/Santiago/p4d0/2012(H3N2)) |
| AIE53868 | M1 | H3N2 | 2012/06/16 | (A/Santiago/p4d0/2012(H3N2)) |
| AIE53869 | M2 | H3N2 | 2012/06/16 | (A/Santiago/p4d0/2012(H3N2)) |
| AIE53870 | NA | H3N2 | 2012/06/16 | (A/Santiago/p4d0/2012(H3N2)) |
| AIE53871 | NP | H3N2 | 2012/06/16 | (A/Santiago/p4d0/2012(H3N2)) |
| AIE53872 | NS1 | H3N2 | 2012/06/16 | (A/Santiago/p4d0/2012(H3N2)) |
| AIE53873 | NS2 | H3N2 | 2012/06/16 | (A/Santiago/p4d0/2012(H3N2)) |
| AIE53874 | PA-X | H3N2 | 2012/06/16 | (A/Santiago/p4d0/2012(H3N2)) |
| AIE53875 | PA | H3N2 | 2012/06/16 | (A/Santiago/p4d0/2012(H3N2)) |
| AIE53876 | PB1 | H3N2 | 2012/06/16 | (A/Santiago/p4d0/2012(H3N2)) |
| AIE53877 | PB2 | H3N2 | 2012/06/16 | (A/Santiago/p4d0/2012(H3N2)) |
